# TLR3 serves as a novel diagnostic and prognostic biomarker and is closely correlated with immune microenvironment in three types of cancer

**DOI:** 10.3389/fgene.2022.905988

**Published:** 2022-11-07

**Authors:** Xiong Zou, Yi Guo, Zengnan Mo

**Affiliations:** ^1^ Department of Urology, The First Affiliated Hospital of Guangxi Medical University, Nanning, China; ^2^ Center for Genomic and Personalized Medicine, Guangxi Medical University, Nanning, China; ^3^ Guangxi Collaborative Innovation Center for Genomic and Personalized Medicine, Nanning, China; ^4^ Guangxi Key Laboratory for Genomic and Personalized Medicine, Guangxi Key Laboratory of Colleges and Universities, Nanning, China; ^5^ Department of Gynecology and Obstetrics, The First Affiliated Hospital of Guangxi Medical University, Nanning, China

**Keywords:** TLR3, KIRC, LGG, PAAD, biomarker

## Abstract

**Background:** Toll-like receptor 3 (TLR3) plays an important role in both innate and adaptive immunity, but the prognostic value of TLR3 in heterogeneous tumors and the correlations between TLR3 expression and immune infiltration of heterogeneous tumors remain unclear.

**Methods:** We investigated the expression of TLR3 in a variety of tumors and focused on the diagnostic and prognostic values of TLR3 in kidney renal clear cell carcinoma (KIRC), pancreatic adenocarcinoma (PAAD) and brain lower grade glioma (LGG) by GEPIA, DriverDBv3, UALCAN, TIMER, LinkedOmics, STRING, GeneMANIA and FunRich, as well as the possible mechanisms of TLR3 affecting tumor prognosis were discussed. Additionally, real-time fluorescence quantitative polymerase chain reaction (RT-qPCR) was used to validate TLR3 expression in early KIRC. We also compared the expression of TLR3 in the plasma of early KIRC patients and normal controls by enzyme linked immunosorbent assay (ELISA).

**Results:** TLR3 expression was significantly different in multiple tumors compared with paracancerous nontumor tissues. Elevated expression of TLR3 contributed to the prolonged survival outcome in KIRC patients. Suppressed expression of TLR3 contributed to the prolonged survival outcome in LGG and PAAD patients. Moreover, TLR3 was significantly elevated in stage1, grade1 and N0 of KIRC. The expression and function of TLR3 in KIRC, LGG and PAAD were closely related to tumor immune microenvironment. TRAF6 was a key gene in the interactions between TLR3 and its interacting genes. Finally, the results of RT-qPCR and ELISA indicated that TLR3 expression levels were significantly raised in renal tissue and plasma of early KIRC patients.

**Conclusion:** TLR3 has the potential to be a diagnostic biomarker of KIRC, LGG and PAAD as well as a biomarker for evaluating the prognosis of KIRC, LGG and PAAD, particularly for the early diagnosis of KIRC. TLR3 affects tumors mainly by acting on the immune microenvironment of KIRC, LGG and PAAD. These findings could lead to new insights into the immunotherapeutic targets for KIRC, LGG, and PAAD.

## 1 Introduction

Toll-like receptors (TLRs) are the key to identifying pathogenic microorganisms and controlling immune response (Marin-Acevedo et al., 2018). Some studies have shown that the release of damage associated molecular patterns (DAMPs) by dead cancer cells can exert an effective and persistent anti-cancer immune response by activating TLRs on host immune cells (Galluzzi et al., 2017; Garg and Agostinis, 2017; Galluzzi et al., 2020). Toll-like receptor 3 (TLR3), a crucial member of TLRs (Bianchi et al., 2017), whose absence can lead to autoimmune diseases, septicemia, chronic inflammation and cancer, among other pathological conditions (Le Naour et al., 2020). TLR3 is expressed on the endosome membrane and is a key molecule for the recognition of viral double-stranded RNA (dsRNA) (Wang et al., 2020). TLR3 is not only a target for antiviral therapy, but also a potential target for antitumor therapy, because TLR3 agonists can help initiate adaptive immunity (Cheng and Xu, 2010). Previous studies on TLR3 were mainly focused on inflammation and infection, but there were few studies on the expression of TLR3 in tumors, the effect of TLR3 on tumor prognosis and the related biological functions of TLR3.

In this study, GEPIA, DriverDBv3, UALCAN and TIMER databases were used to analyze the relationships between the expression of TLR3 in different tumor types and tumor prognosis. Moreover, we investigated the associations of TLR3 expression and somatic copy number alterations (SCNA) with the immune cells infiltration levels in kidney renal clear cell carcinoma (KIRC), brain lower grade glioma (LGG) and pancreatic adenocarcinoma (PAAD). Gene set enrichment analysis (GSEA) was performed on TLR3 of KIRC, LGG and PAAD using the LinkedOmics database. What’s more, we constructed the protein-protein interaction (PPI) network and analyzed the functions of TLR3 and its interacting genes. At the same time, we also obtained the key gene connecting TLR3 and its interacting genes by FunRich. Finally, we employed real-time fluorescence quantitative polymerase chain reaction (RT-qPCR) and enzyme linked immunosorbent assay (ELISA) to verify TLR3 expression in early KIRC patients.

## 2 Materials and methods

### 2.1 GEPIA

The functions of the GEPIA website are varied, such as analyzing RNA expression in tumor and paracancer tissues and assessing cancer prognosis by gene expression (Tang et al., 2017). Patient data in GEPIA are obtained from TCGA and GTEx. We studied the expression levels of TLR3 in 33 cancers relative to normal tissues by GEPIA, and plotted the survival curve of tumors with differential expression of TLR3 (Log-rank test).

### 2.2 DriverDBv3

DriverDBv3, a cancer multi-omics database, contains clinical and gene-level data such as survival curve, RNA expression, miRNA expression level, somatic mutation and methylation (Liu et al., 2020). Using DriverDBv3 database, we studied the effect of TLR3 expression on the prognosis of three types of cancers (Log-rank test).

### 2.3 UALCAN

UALCAN is a website for analyzing the TCGA database. Users can verify the levels of gene expression in different cancer types, chart patient survival and gene expression information, and evaluate the expression of specific genes in different pathological states of cancer (Chandrashekar et al., 2017). In this study, TLR3 expression in different grades, stages and lymph node metastases of KIRC, LGG and PAAD was determined by UALCAN.

### 2.4 TIMER

TIMER is an immune-related cancer web server that allows users to evaluate tumor characteristics based on specific functional parameters (Li et al., 2017). By means of TIMER, we investigated the expression of TLR3 in different tumors and the correlations of TLR3 expression with immune cells infiltration levels of LGG, KIRC and PAAD (Spearman’s correlation). In addition, we also studied the relationship between the somatic copy number alterations of TLR3 and the levels of tumor immune infiltration through the “SCNA” module of TIMER (Wilcoxon rank-sum test).

### 2.5 LinkedOmics

Linkedomics is a tool for exploring multi-omics data from multiple cancer types in the TCGA (Vasaikar et al., 2018). Using the “Gene Set Enrichment Analysis” of LindkeOmics, “KEGG Pathway enrichment analysis” and “GO analysis” of TLR3 were performed. The “Rank Criteria”, “Minimum Number of Genes (Size)” and “Simulations” were set as “meta *p*-value”, “3” and “500”, respectively.

### 2.6 STRING

STRING is an online software that can conduct direct or indirect comprehensive analysis of related genes of selected genes (Szklarczyk et al., 2019). The interacting genes of TLR3 were obtained by STRING.

### 2.7 GeneMANIA

GeneMANIA can be used to analyze co-expression, PPI and related functions between genes (Warde-Farley et al., 2010). In our study, the main functions of TLR3 and its interacting genes were understood through GeneMANIA.

### 2.8 FunRich

FunRich (3.1.3 exe), an independent software tool, is mainly used for functional enrichment and interaction network analysis of proteins and genes (Fonseka et al., 2021). We identified the most critical genes related to TLR3 and its interacting genes using FunRich. Then we made an in-depth analysis of the most critical gene in order to further understand TLR3 and its i interacting genes.

### 2.9 Real-time fluorescence quantitative polymerase chain reaction analysis

The First Affiliated Hospital of Guangxi Medical University provided KIRC and paracancerous tissues, which were preserved at -80°C. Total RNA Kit I (R6834, Omega) was used to extract Total RNA. RNA was reverse transcribed into cDNA in line with the instructions using the PrimeScript RT reagent kit (RR036a, Takara, Kyoto, Japan), and then we detected the cDNA using LightCycler® 96 Instrument (06924204001, Roche) and FastStart Essential DNA Green Master (Roche). Each sample was set up to repeat the determination three times. The internal reference was glyceraldehyde-3-phosphate dehydrogenase (GADPH), and the relative expression of TLR3 mRNA was determined using the 2^−ΔΔCt^ algorithm. (TLR3 forward primer: 5′-TTG​CCT​TGT​ATC​TAC​TTT​TGG​GG-3'; TLR3 reverse primer: 5′-TCA​ACA​CTG​TTA​TGT​TTG​TGG​GT-3′).

### 2.10 Enzyme linked immunosorbent assay

Blood samples were collected from 7 patients with early KIRC before and 3 days after operation by EDTA anticoagulant tube in the First Affiliated Hospital of Guangxi Medical University. Blood samples from 7 healthy adults were collected from the physical examination department. All blood samples were centrifuged at 3,000 rpm for 20 min, and the upper plasma was collected and stored at -80°C. The expression of TLR3 protein in plasma was detected according to ELISA kit (mlbio, ml027584) instruction.

### 2.11 Statistical analysis

The expression of TLR3 mRNA and protein between the two groups was analyzed by *t*-test using GraphPad Prism 7. All statistical results in this study were considered to be statistically significant if *p* < 0.05.

## 3 Results

### 3.1 TLR3 expression levels in different tumor types

**FIGURE 1:**
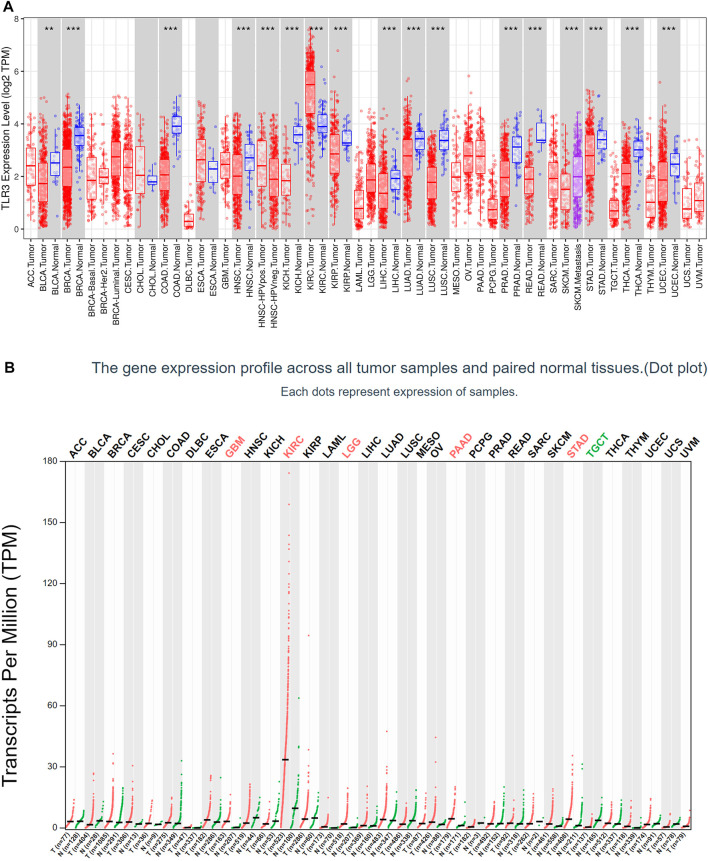
The expression of TLR3 in different cancers. **(A)** The expression levels of TLR3 gene in different cancer tissues and adjacent normal tissues were obtained by TIMER. **(B)** Expression levels of TLR3 transcript obtained from GEPIA in different cancer tissues and paired normal tissues. *p < 0.05; **p < 0.01; ***p < 0.001.

First of all, we investigated the expression of TLR3 in 33 kinds of tumors compared with normal tissues by TIMER. Elevated expression levels of TLR3 were observed in KIRC, while suppressed expression levels of TLR3 were observed in bladder urothelial carcinoma (BLCA), head and neck squamous cell carcinoma (HNSC), breast invasive carcinoma (BRCA), liver hepatocellular carcinoma (LIHC), colon adenocarcinoma (COAD), kidney chromophobe (KICH), lung squamous cell carcinoma (LUSC), kidney renal papillary cell carcinoma (KIRP), lung adenocarcinoma (LUAD), rectum adenocarcinoma (READ), prostate adenocarcinoma (PRAD), stomach adenocarcinoma (STAD), thyroid carcinoma (THCA) and uterine corpus endometrial carcinoma (UCEC) (Figure 1A). Then, we used GEPIA to study the expression levels of TLR3 in 33 cancer types. The results suggested that the TLR3 expression levels in glioblastoma multiforme (GBM), LGG, KIRC, PAAD and STAD were significantly elevated compared with those in adjacent nontumor tissues, but the expression levels of TLR3 in testicular germ cell tumors (TGCT) were significantly decreased (Figure 1B). There were differences between the results generated by the TIMER and GEPIA. Because the TIMER analysis was only based on TCGA, while the GEPIA analysis was based on TCGA and GTEx data.

### 3.2 A biomarker of potential prognosis of KIRC, LGG and PAAD: TLR3

**FIGURE 2:**
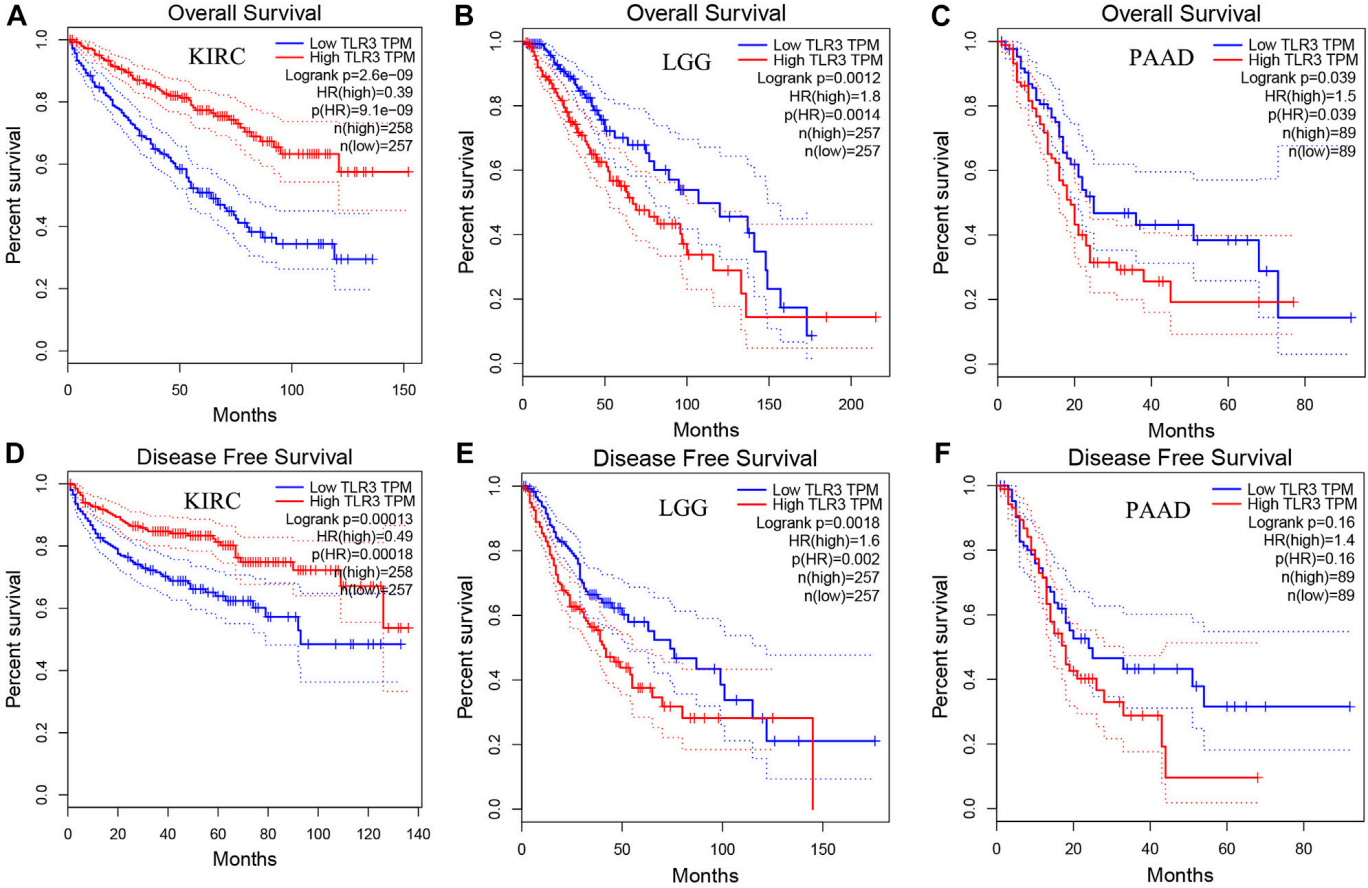
Comparing the effects of high and low expression of TLR3 on KIRC, LGG and PAAD survival outcomes by GEPIA. The effects of high and low expression of TLR3 on OS **(A–C)** and DFS **(D–F)** of KIRC, LGG and PAAD.

**FIGURE 3:**
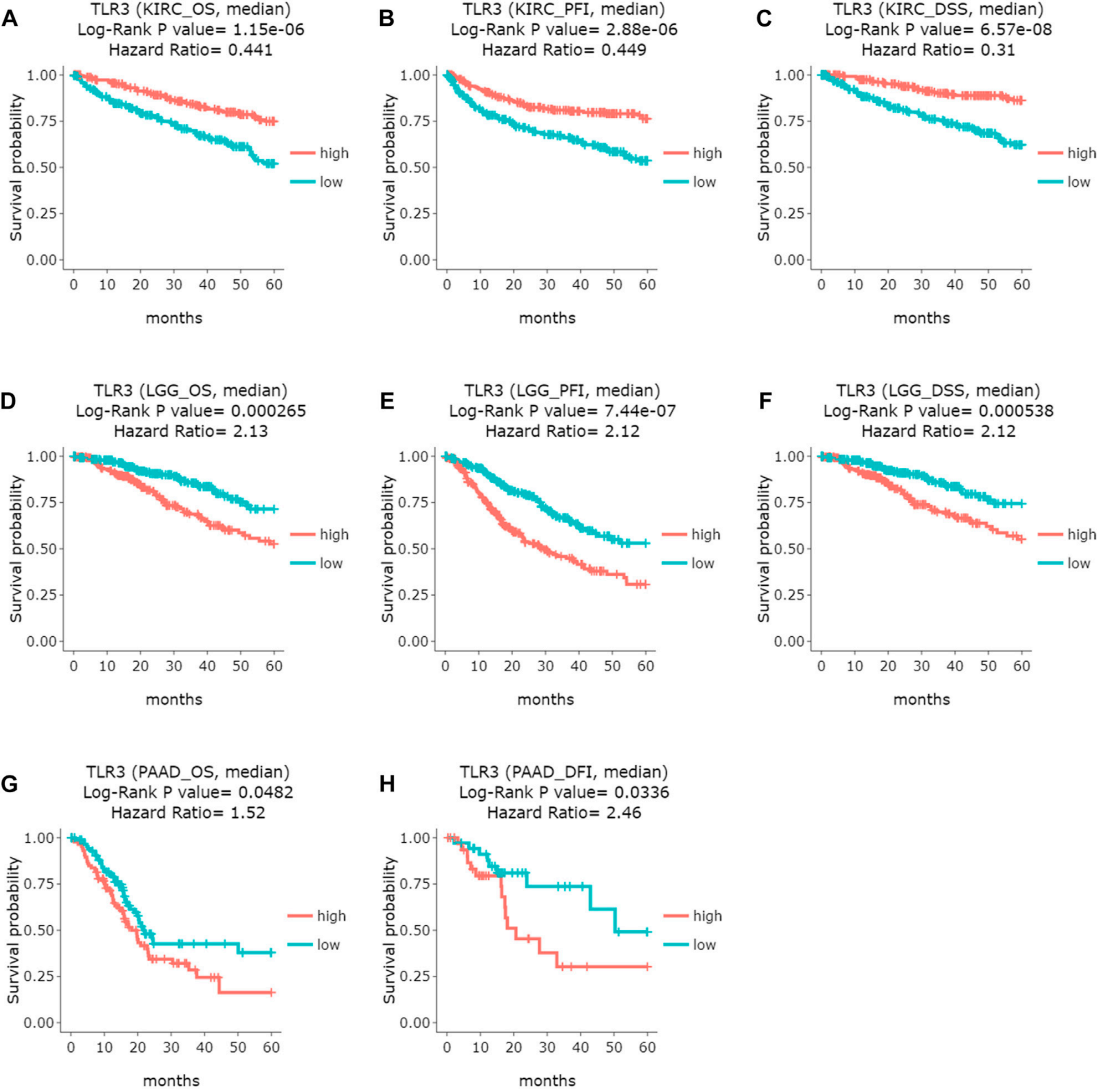
Comparing the effects of high and low expression of TLR3 on KIRC, LGG and PAAD survival outcomes by DriverDBv3. **(A–C)** The effects of high and low expression of TLR3 on OS, PFI and DFS of KIRC. **(D–F)** The effects of high and low expression of TLR3 on OS, PFI and DSS of LGG. **(G,H)** The effects of high and low expression of TLR3 on OS and DFI of PAAD.

Next, we used GEPIA to conduct survival analysis on the above tumors with differential expression of TLR3 in order to understand the influence of TLR3 expression on their prognosis. To our surprise, although TLR3 was differentially expressed in multiple tumors compared with the corresponding paracancerous nontumor tissues, it only affected the overall survival (OS) of KIRC, LGG and PAAD. Elevated expression of TLR3 was beneficial to prolong the OS of KIRC (Figure 2A), while suppressed expression of TLR3 was beneficial to prolong the OS of LGG (Figure 2B) and PAAD (Figure 2C). The effect of TLR3 on the OS of other tumors with TLR3 differential expression was shown in Supplementary Figure S1. Next, we further investigated the associations between TLR3 and these three tumors. We found that elevated expression of TLR3 was beneficial for prolonging disease-free survival (DFS) in KIRC patients (Figure 2D), while suppressed expression of TLR3 was beneficial for prolonging DFS in LGG (Figure 2E), but TLR3 expression had no significant effect on DFS in PAAD (Figure 2F). In order to confirm the prognostic value of TLR3 expression in three kinds of tumors, we further investigated the associations of TLR3 expression with the prognosis of KIRC, LGG and PAAD by DriverDBv3. The results indicated that TLR3 overexpression was beneficial to prolong the OS (Figure 3A), platinum-free treatment interval (PFI) (Figure 3B) as well as disease specific survival (DSS) (Figure 3C) of KIRC, while suppressed expression of TLR3 was beneficial for prolonging OS (Figure 3D), PFI (Figure 3E) and DSS (Figure 3F) of LGG. Moreover, suppressed expression of TLR3 was beneficial for prolonging OS (Figure 3G) and disease-free interval (DFI) (Figure 3H) of PAAD. Therefore, all these results demonstrated that TLR3 expression was associated with the prognosis of KIRC, LGG, and PAAD. Additionally, the expression of TLR3 exhibited different effects on the survival outcome of KIRC, LGG and PAAD.

### 3.3 The expression of TLR3 in different stages, grades, and lymph node metastases of KIRC, LGG, PAAD

**FIGURE 4:**
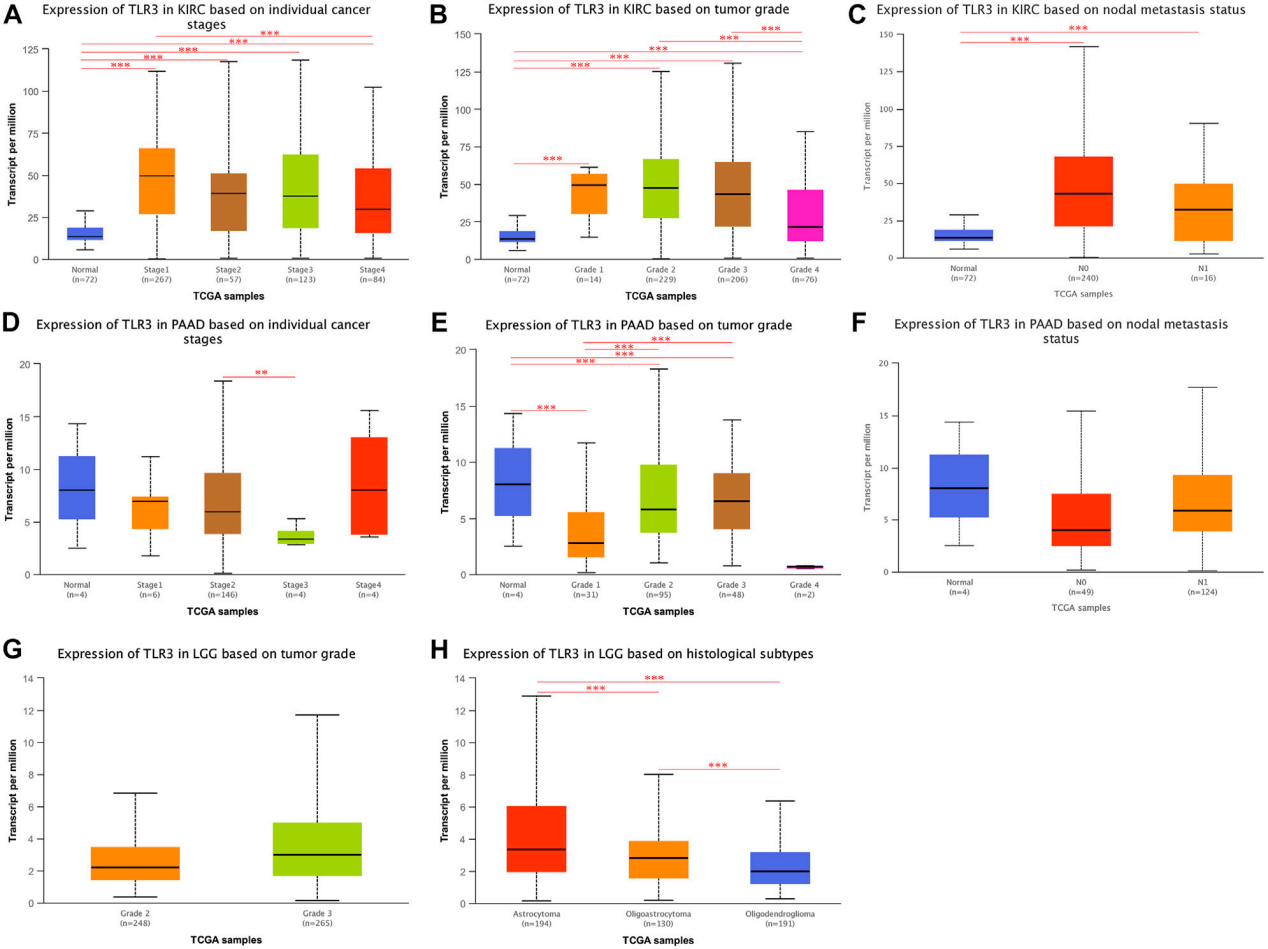
The expression of TLR3 transcript at different phases of KIRC, PAAD and LGG. **(A–C)** Box plots showing the expression of TLR3 transcript based on cancer stages, tumor grades and lymph nodal metastasis status of KIRC, respectively. **(D–F)** Box plots showing the expression of TLR3 transcript based on cancer stages, tumor grades and lymph nodal metastasis status of PAAD, respectively. **(G)** Box plot showing the expression of TLR3 transcript based on tumor grades of LGG. **(H)** Box plot showing the expression of TLR3 transcript based on histological subtypes of LGG. *p < 0.05; **p < 0.01; ***p < 0.001.

Next, we continued to explore the expression of TLR3 in different stages, grades, and lymph node metastases of KIRC, LGG, and PAAD by UALCAN. To our surprise, the expression of TLR3 in different stages, grades and lymph node metastases of KIRC was significantly different from those in adjacent nontumor tissues. Moreover, the TLR3 expression in stage1 (Figure 4A), grade1 (Figure 4B) and N0 (Figure 4C) of KIRC was significantly increased compared with that in paracancerous nontumor tissues, which demonstrated that TLR3 was likely to be an early diagnostic biomarker for KIRC. In addition, the TLR3 expression in stage1 of KIRC was significantly higher than that in stage4 of KIRC (Figure 4A), and the TLR3 expression in grade2 and grade3 of KIRC was significantly higher than that in grade4 of KIRC (Figure 4B). However, the expression of TLR3 in different stages and lymph node metastases of PAAD was no significant difference compared with normal tissues, but the expression of TLR3 in grade3 of PAAD was significantly higher than that in grade1 and grade2 of PAAD (Figures 4D–F). However, we considered that the number of adjacent normal samples of PAAD in UALCAN analysis was only 4, so the comparative expression of TLR3 in PAAD and adjacent normal tissues was lack of representativeness. Additionally, we found that there was no significant difference in the expression of TLR3 in different grades of LGG (Figure 4G), and there was a lack of relevant data on the expression of TLR3 in different stages and lymph node metastases of LGG. Nevertheless, we found that there were significant differences in the expression of TLR3 in different histological subtypes of LGG. These results suggested that TLR3 had the potential to be used as a diagnostic biomarker of KIRC, LGG and PAAD, especially as a biomarker for early diagnosis of KIRC and for pathological classification of LGG.

### 3.4 The associations of TLR3 expression and somatic copy number alterations with the immune cells infiltration levels in KIRC, LGG and PAAD

**FIGURE 5:**
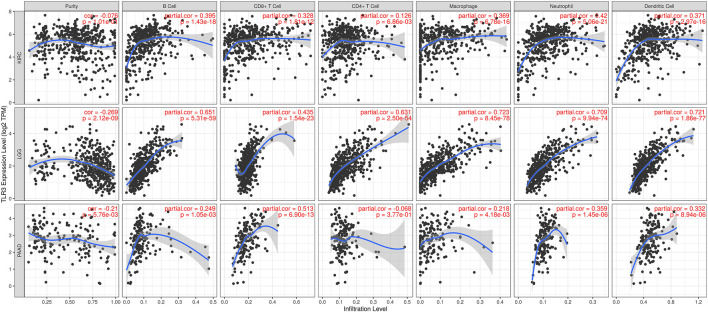
Associations of TLR3 expression with infiltration levels of immune cells in KIRC, LGG and PAAD.

**FIGURE 6:**
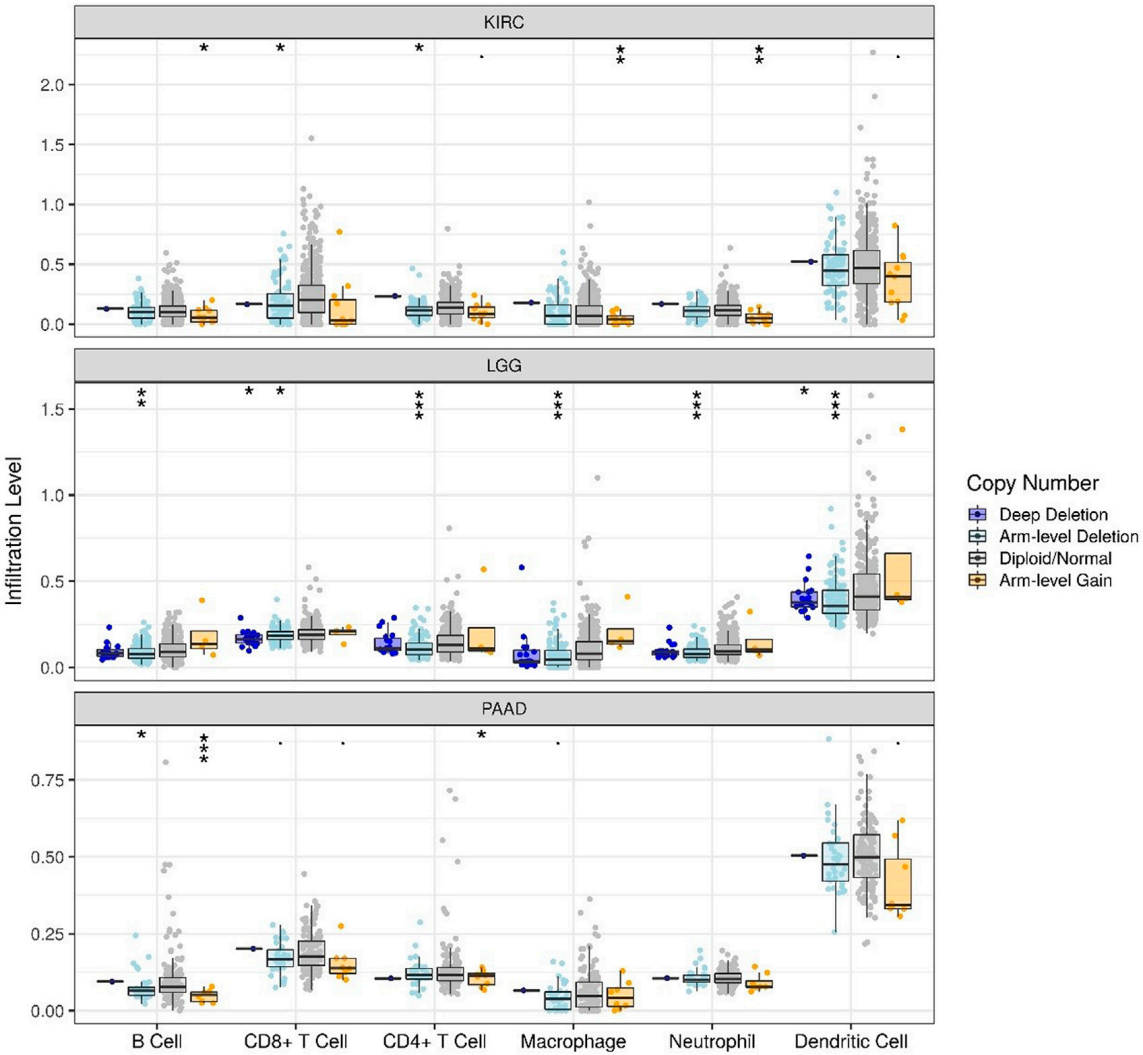
Associations of immune cells infiltration levels with somatic copy number alterations for TLR3 in KIRC, LGG and PAAD. *p < 0.05; **p < 0.01; ***p < 0.001.

The correlations of TLR3 expression with immune cells infiltration levels in KIRC, LGG and PAAD were investigated by TIMER. We discovered the TLR3 expression in KIRC and LGG was positively correlated with the infiltrations of CD8+T cells, B cells, neutrophils, CD4+T cells, macrophages and dendritic cells (DCs). However, TLR3 expression in PAAD was positively associated with the infiltrations of B cells, macrophages, CD8+T cells, neutrophils and DCs, but TLR3 expression was not detectable association with CD4+T cells (Figure 5). We also explored the relationships between somatic copy number alterations (SCNA) of TLR3 and the levels of immune cells infiltration in KIRC, LGG, and PAAD. Our study showed that arm-level gain of TLR3 in KIRC was significantly associated with the infiltrations of neutrophils, B cells and macrophages, while arm-level deletion of TLR3 in KIRC was significantly associated with the infiltrations of CD4+T cells and CD8+T cells. Moreover, arm-level deletion of TLR3 in LGG was apparent association with the infiltrations of DCs, macrophages, B cells, neutrophils, CD4+T cells and CD8+T cells, while deep deletion of TLR3 in LGG was obviously associated with the infiltrations of DCs and CD8+T cells. Additionally, arm-level deletion of TLR3 in PAAD was significantly associated with the infiltrations of B cells, while arm-level gain of TLR3 in PAAD was significantly associated with the infiltrations of CD4+T cells and B cells (Figure 6).

### 3.5 Gene set enrichment analysis of TLR3 in KIRC, LGG and PAAD

**FIGURE 7:**
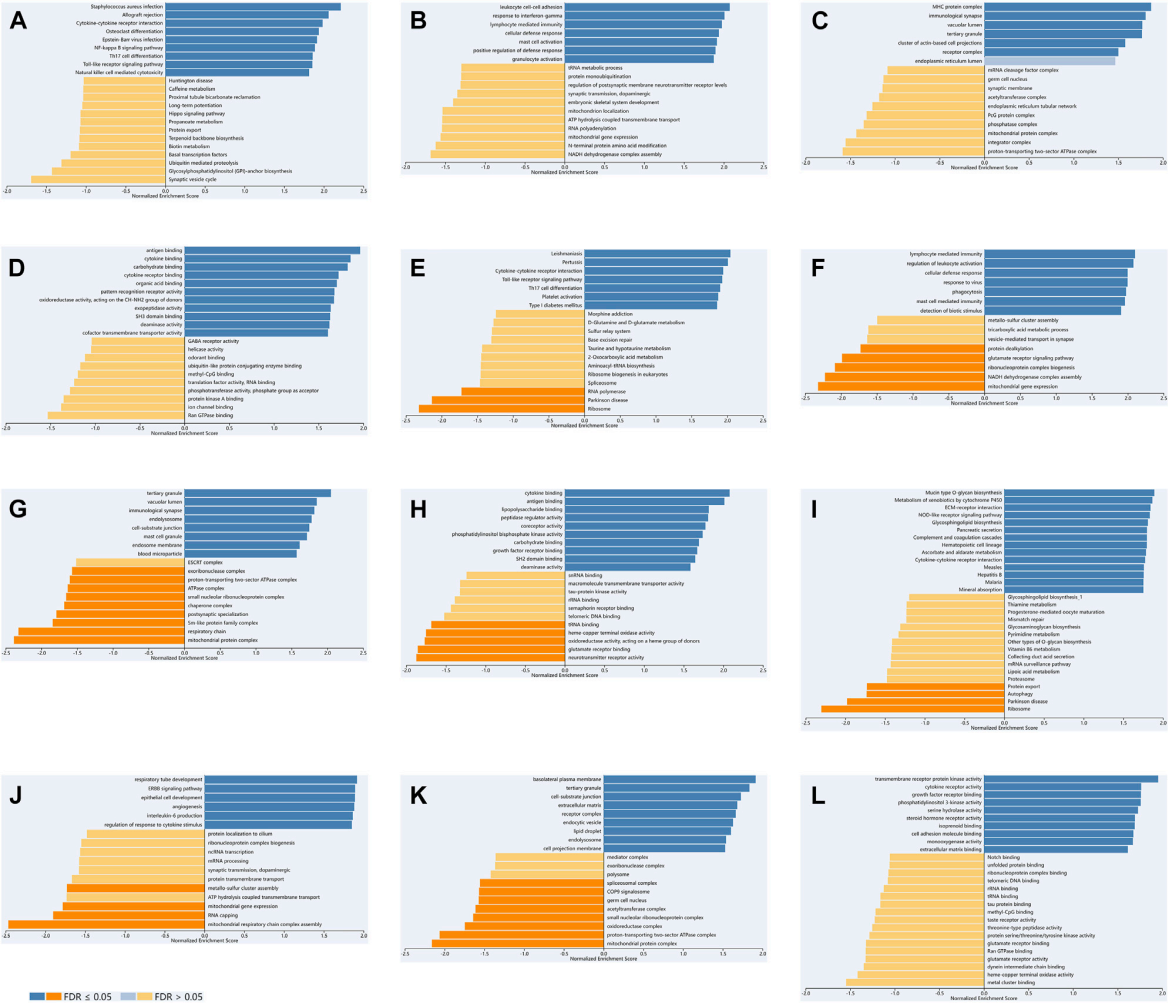
The GO and KEGG analyses of TLR3 in KIRC, LGG and PAAD based on LinkedOmics. KEGG **(A)**, BP **(B)**, CC **(C)** and MF **(D)** of TLR3 in KIRC. KEGG **(E)**, BP **(F)**, CC **(G)** and MF **(H)** of TLR3 in LGG. KEGG **(I)**, BP **(J)**, CC **(K)** and MF **(L)** of TLR3 in PAAD.

We used LinkedOmics to select data sets of KIRC, LGG and PAAD for enrichment analysis of TLR3. We explored GO and KEGG analyses of TLR3-related. Cytokine-cytokine receptor interaction, NF-kappa B signaling pathway, Th17 cell differentiation, toll-like receptor signaling pathway and natural killer cell mediated cytotoxicity were KEGG pathways mainly related to TLR3 in KIRC (Figure 7A). TLR3 in KIRC was involved in many biological processes (BP), mainly including leukocyte cell-cell adhesion, response to interferon-gamma, lymphocyte mediated immunity, cellular defense response and mast cell activation, (Figure 7B). In KIRC, the main cellular components (CC) involved in TLR3 were MHC protein complex, immunological synapse and receptor complex (Figure 7C). Antigen binding, cytokine receptor binding and pattern recognition receptor activity were the main molecular functions (MF) of TLR3 in KIRC (Figure 7D). In LGG and PAAD, the results of GO and KEGG analyses of TLR3 were shown in Figures 7E-L.

### 3.6 PPI network and functional analysis of TLR3 and its interacting genes

**FIGURE 8:**
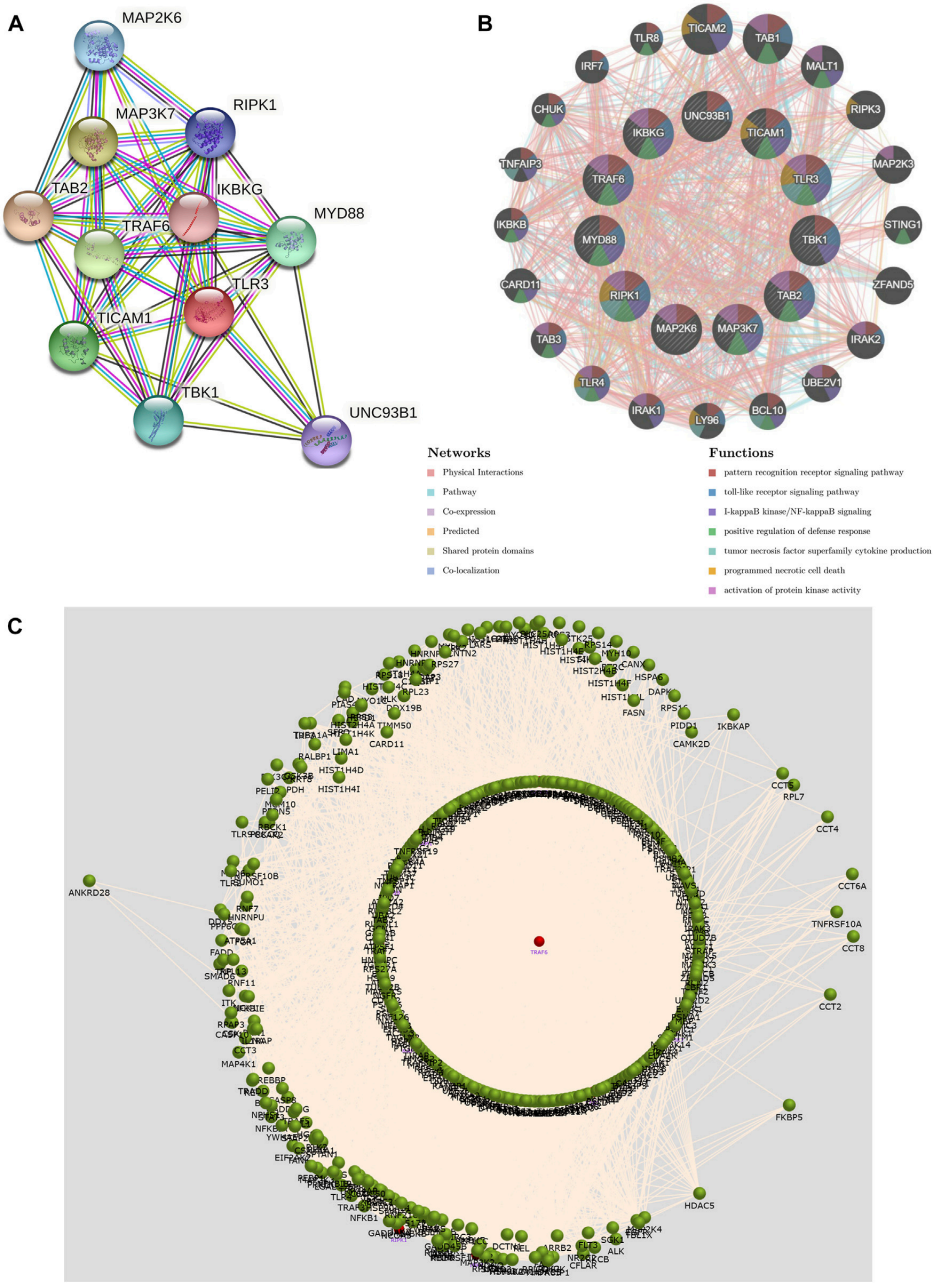
The PPI network and function of TLR3 and its interacting genes and the key gene that regulates their expression. **(A)** The PPI network of TLR3. **(B)** The PPI network and functions of TLR3 and its interacting genes. **(C)** Interaction plot showing that the key gene affecting TLR3 and its interacting genes is TRAF6.

We constructed a PPI network of TLR3 through STRING. The PPI network showed a complex association of TLR3 with other genes (Figure 8A). Next, we performed functional analysis on TLR3 and its interacting genes through GeneMANIA. The results demonstrated that the functions of TLR3 and its interacting genes were mainly focused on pattern recognition receptor signaling pathway, I-kappaB kinase/NF-kappaB signaling, toll-like receptor signaling pathway, tumor necrosis factor superfamily cytokine production, positive regulation of defense response, programmed necrotic cell death and activation of protein kinase activity (Figure 8B). These results suggested that TLR3 and its interacting genes were closely related to immune and anti-tumor effects. Next, we used FunRich to obtain the key gene connecting TLR3 and its interacting genes. These results demonstrated that TRAF6 was a key gene in the interactions between TLR3 and its interacting genes (Figure 8C).

### 3.7 Verification the expression levels of TLR3 in early KIRC patients

**FIGURE 9:**
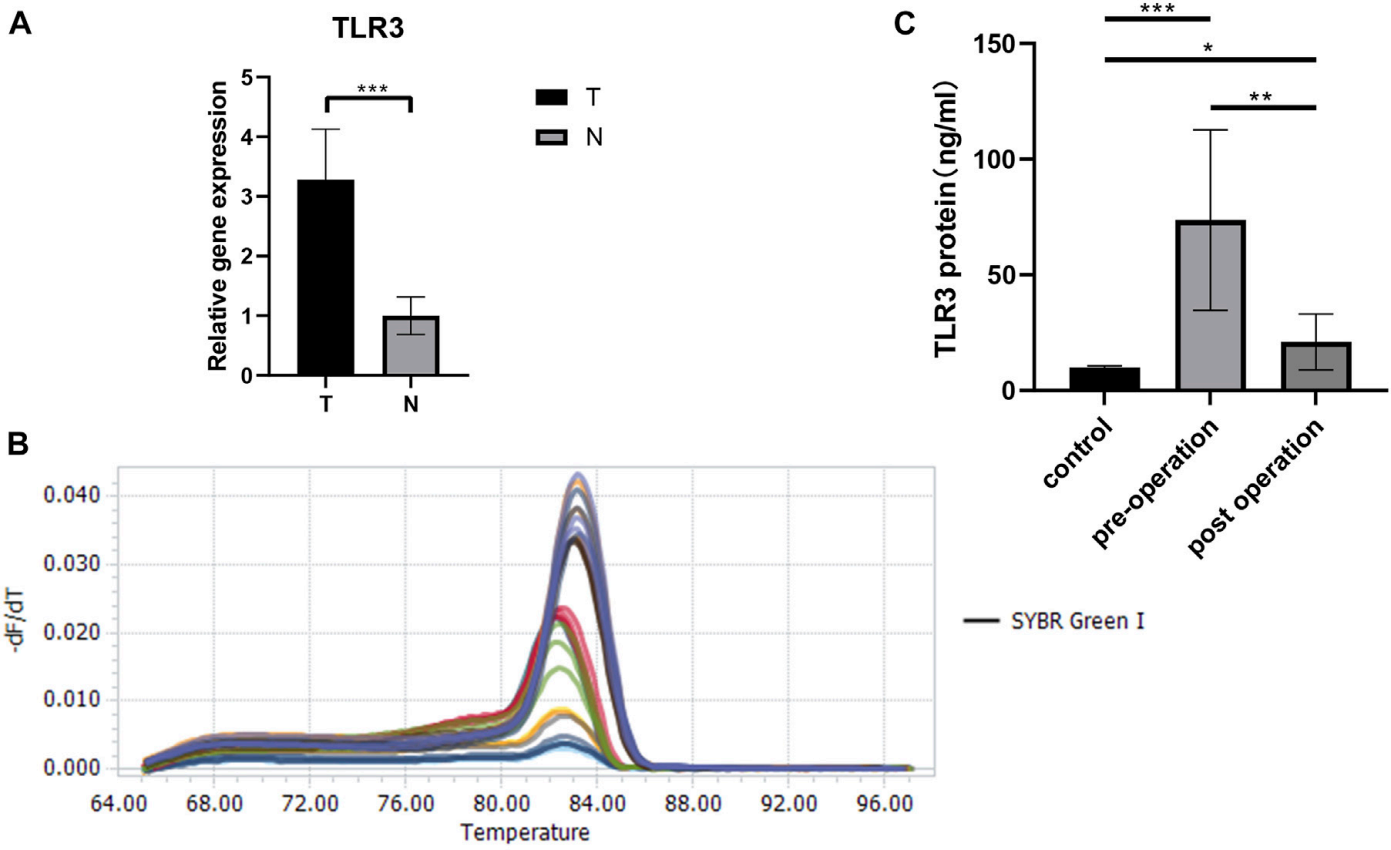
The results of RT-qPCR and ELISA. **(A)** The expression levels of TLR3 mRNA in early stage of KIRC tissues and adjacent normal tissues. **(B)** The melting peaks of RT-qPCR. **(C)** Comparison of TLR3 protein levels in plasma of patients with early KIRC before operation, 3 days after operation and healthy adults. T: KIRC tissues; N: adjacent normal tissues; control: healthy adults; *p < 0.05; **p < 0.01; ***p < 0.001.

Last but not least, we verified the TLR3 mRNA expression levels between tumor tissues and paracancerous nontumor tissues of early KIRC patients. The characteristics of patients used to study the expressions of TLR3 mRNA were shown in Table1. According to RT-qPCR data, the relative expression levels of TLR3 mRNA in early KIRC were considerably higher than that in paracancerous nontumor tissues (Figure 9A). The melting peaks of RT-qPCR were shown in Figure 9B, which demonstrated that the designed primers were specific. Moreover, we compared the expression of TLR3 protein in plasma of patients with early KIRC and healthy adults by ELISA. The basic information of KIRC patients and healthy adults used in the ELISA was shown in Supplementary Table S1. The results of ELISA showed that the expression of TLR3 protein in plasma of patients with KIRC before operation was significantly higher than that of healthy adults, and the expression of TLR3 protein in plasma of patients with KIRC 3 days after operation was significantly lower than that before operation, but still higher than that of healthy adults (Figure 9C).

**TABLE 1:** The characteristics of patients for RT-qPCR.

Patients	Sex	Years of age	Tumor location	Tumor size (cm)	TNM stage	Histological type
Patient1	Male	40	Right	4.8 × 4 × 3.5	T_1_N_0_M_0_	KIRC
Patient2	Male	50	Right	2.5 × 2 × 2	T_1_N_0_M_0_	KIRC
Patient3	Female	41	Left	4.5 × 4 × 4	T_1_N_0_M_0_	KIRC

## 4 Discussion

The most common cancer in renal cell carcinoma (RCC) is KIRC, which accounts for around 75% of all RCC (Linehan and Ricketts, 2019). Early stage of KIRC can be surgically excised to achieve excellent results, however, treating advanced KIRC continues to be a serious challenge (Petitprez et al., 2021). In addition, clinicians currently rely on the stage of tumor lymph node metastasis (TNM) to estimate the prognosis of KIRC patients (Pichler et al., 2013; Qin et al., 2013; Zheng et al., 2017), but the prognosis of patients with the same TNM stage may vary greatly (Wei et al., 2019). However, the accuracy of prognosis prediction can be improved by stratifying the prognosis using biomarkers (such as the expressions of specific genes) [ (Tamayo et al., 2011); (Zou and Mo, 2021)], therefore, it is necessary to search for specific biomarkers for prognosis and early diagnosis of KIRC, as well as for treatment of KIRC.

Glioma, a highly heterogeneous tumor (Poff et al., 2019), is one of the most prevalent primary brain tumors, accounting for about 70% of malignant brain tumors (Gusyatiner and Hegi, 2018). At present, more and more young people suffer LGG of WHO grade Ⅱ and Ⅲ (Gusyatiner and Hegi, 2018). Although the malignant features of LGG are less than those of high-grade gliomas (WHO grade IV), and the clinical prognosis of LGG is relatively better (Claus et al., 2015), there are no biomarkers that can accurately determine the prognosis of LGG. If we can find a biomarker that can accurately judge the prognosis of LGG patients, it will be of great help to the management and treatment of LGG.

PAAD is a devastating tumor disease that is becoming more common and has the lowest 5-year survival rate of cancers (Siegel et al., 2017). There are similarities between chronic pancreatitis and PAAD in connective tissue hyperplasia and inflammatory infiltration (Zheng et al., 2013). At the same time, there is growing evidence that the evolution and maintenance of PAAD are influenced by complicated inflammatory and immunosuppressive environments (Wormann et al., 2014). Therefore, further studies on the association between PAAD and immunity can improve our understanding of the pathogenesis of PAAD and contribute to the development of immunotherapy for PAAD.

TLR3, mainly located in the endoplasmic reticulum and intracellular endocytic compartment, can recognize oligonucleotides from itself and microorganisms (Hussein et al., 2014). TLR3 is expressed in a variety of immune cells, such as dendritic cells (DCs), natural killer cells (NK) cells and macrophages, and TLR3 exerts a momentous effect in the activation of innate immunity (Iwasaki and Medzhitov, 2004). However, TLR3 can also be activated in non-immune cells, such as fibroblasts and vascular endothelial cells (Pirher et al., 2017). Interestingly, there is growing evidence that TLR3 is also generated in cancer cells, and the generation of TLR3 in cancer cells may exert an opposite effect in different cancer progression (Zheng et al., 2021). Therefore, we investigated TLR3 expression in different cancers and its effect on cancer prognosis. At the same time, we studied the interacting genes and related functions of TLR3 in order to understand the possible mechanisms of TLR3 affecting cancers.

First of all, we explored TLR3 expression levels in multiple cancers using GEPIA and TIMER databases. Compared with paracancerous nontumor tissues, the expression levels of TLR3 were significantly elevated in GBM, KIRC, LGG, PAAD and STAD, while suppressed in TGCT, COAD, BLCA, KICH, BRCA, HNSC, LUAD, PRAD, READ, KIRP, LIHC, THCA, LUSC, STAD and UCEC. Secondly, we found that TLR3 was differentially expressed in varieties of tumors compared with paracancerous nontumor tissues, but it only affected the survival time of KIRC, LGG and PAAD. Overexpression of TLR3 was associated with prolonged PFI, DFS, OS and DSS in patients with KIRC, while suppressed expression of TLR3 was associated with prolonged PFI, DFS, OS and DSS in patients with LGG, and suppressed expression of TLR3 was associated with prolonged DFI and OS in patients with PAAD. These results suggested that TLR3 played different biological roles in different tumors. Similar conclusions could be drawn from other cancer studies. For example, high expression of TLR3 prolonged the survival time of patients with non-small-cell lung cancer by inducing apoptosis of cancer cells (Bianchi et al., 2020), but high expression of TLR3 could promote the migration of breast cancer cells by increasing the expression of E-cadherin (Bondhopadhyay et al., 2015). Next, we found that the TLR3 expression in stage1, grade1 and N0 of KIRC was significantly increased compared with that in paracancerous nontumor tissues, and the TLR3 expression in stage1 of KIRC was significantly higher than that in stage4 of KIRC, and the TLR3 expression in grade2 and grade3 of KIRC was significantly higher than that in grade4 of KIRC. These results indicated that the TLR3 expression in early and low grade KIRC was significantly higher than that in advanced and high grade KIRC. In addition, the expression of TLR3 in grade3 of PAAD was significantly higher than that in grade1 and grade2 of PAAD. And there were significant differences in the expression of TLR3 in different histological subtypes of LGG. We also verified that there was a significant elevation in the expression of TLR3 mRNA in early KIRC by RT-qPCR. To our excitement, the results of ELISA showed that the expression of TLR3 protein in plasma of patients with early KIRC before operation was significantly higher than that of healthy adults, and the expression of TLR3 protein in plasma of patients with early KIRC 3 days after operation was significantly lower than that before operation, but still higher than that of healthy adults, which indicated that the occurrence of KIRC could directly lead to the increase of TLR3 protein in plasma. Taking all of the results together, we concluded that TLR3 had the potential to be used as a prognostic biomarker of KIRC, LGG and PAAD, especially as a biomarker for early diagnosis of KIRC and for pathological classification of LGG. The prognostic value of TLR3 in KIRC was similar to the results of Liao et al. (Liao et al., 2021), but our results emphasized the changes of TLR3 in early KIRC and the potential role of TLR3 as an early diagnostic biomarker of KIRC.

For the sake of further exploring the possible mechanisms of TLR3 affecting the prognosis of cancers, we also explored the correlations of TLR3 expression with the immune cells infiltration levels in KIRC, LGG and PAAD. The expression of TLR3 in KIRC and LGG was positively correlated with the infiltration levels of CD4+T cells, CD8+T cells, B cells, neutrophils, macrophages and dendritic cells (DCs). On the other hand, TLR3 expression in PAAD was positively correlated with the infiltration levels of B cells, macrophages, CD8+T cells, neutrophils and DCs, but TLR3 expression was not significantly correlated with CD4+T cells. Therefore, the associations of TLR3 with levels of immune cells infiltration suggested that TLR3 played a vital role in regulating tumor immunology of KIRC, LGG and PAAD. Some studies had shown that tumor immune cells infiltration had different prognostic value in different cancers (Zhang et al., 2019; Zuo et al., 2020). In KIRC patients, B cells infiltration prolonged tumor-specific survival (Stenzel et al., 2020), CD8+T cells infiltration prolonged patients’ OS outcome (Zhang et al., 2019), elevated neutrophils abundance was associated with favorable prognosis (Niu et al., 2021), what’s more, infiltration of dendritic cells and T cells and elevated adaptive immune response could effectively inhibit tumor recurrence and metastasis [(Lim et al., 2007); (Ghatalia et al., 2019)]. Therefore, it is reasonable to speculate that the high expression of TLR3 in KIRC tissues may be an active immune response, which is consistent with the conclusion of a recent study on esophageal squamous cell carcinoma (Su et al., 2022). The study of esophageal squamous cell carcinoma demonstrated that a gene highly expressed in tumors and positively related to prognosis recruited a variety of anti-tumor immune cells for the tumor immune microenvironment. However, the specific reason of up-regulated TLR3 expression in KIRC is still unclear, which needs further study. A study on colon cancer may provide us with some inspiration (Cao et al., 2021). For example, further study on the interaction between TLR3 overexpression cells and other cells through single cell RNA sequencing data may help us to understand the specific mechanism of TLR3 up-regulation in KIRC and the relationship between the special expression pattern of TLR3 and the prognosis of KIRC. In LGG patients, elevated levels of immune cells infiltration were associated with poorer prognosis [(Feng et al., 2020); (Zhang et al., 2020)]. In PAAD patients, elevated levels of immune cells infiltration were associated with decreased OS (Ju et al., 2020), and patients at high risk had high levels of immune cells infiltration (Xu et al., 2021). Our results were similar to the results of these predecessors, but further confirmed the link between three types of cancer and immunity. Moreover, we also explored the correlations between SCNA of TLR3 and the immune cells infiltration levels in KIRC, LGG, and PAAD. Our study showed that arm-level gain of TLR3 in KIRC was significantly associated with the infiltrations of neutrophils, B cells and macrophages, while arm-level deletion of TLR3 in KIRC was significantly associated with the infiltrations of CD4+T cells and CD8+T cells. Moreover, arm-level deletion of TLR3 in LGG was considerably associated with the infiltrations of DCs, macrophages, B cells, neutrophils, CD8+T cells and CD4+T cells, while deep deletion of TLR3 in LGG was significantly associated with the infiltration levels of DCs and CD8+T cells. Additionally, arm-level deletion of TLR3 in PAAD was considerably associated with the infiltrations of B cells, while arm-level gain of TLR3 in PAAD was significantly correlated with the infiltrations of CD4+T cells and B cells. These results further supported a strong association of TLR3 with the levels of immune cells infiltration in three types of cancer.

For a better understanding of TLR3 in KIRC, LGG, and PAAD, we explored GO and KEGG analyses of TLR3-related. Although the results of GO and KEGG analyses of TLR3 were different in KIRC, LGG and PAAD, one common feature of all results was that TLR3 was closely related to the immune process. TLR3 was involved in a variety of BP, CC and MF, mainly including response to interferon-gamma, mast cell activation, macrophage activation, pattern recognition receptor activity and cytokine binding. Interferon is a kind of molecule with multiple effects, including anti-tumor effect and cancer-promoting effect, which plays a vital role in tumor immune microenvironment [(Castro et al., 2018); (Snell et al., 2017)]. Activation of mast cells and macrophages can be used in cancer treatment (Eissmann et al., 2019). KEGG analysis identified the signaling pathways involved by TLR3, mainly including Th17 cell differentiation, toll-like receptor signaling pathway and NOD-like receptor signaling pathway. The imbalance of NOD-like receptor activation is involved in the pathogenesis of tumors, but its role in the development and progression of different cancers is quite different (Moossavi et al., 2018). The results of our study and previous researches demonstrated that TLR3 was closely related to the tumor immune microenvironment (Zou et al., 2022), and this study provided an explanation for the different prognostic outcome of TLR3 in different cancers.

Additionally, we investigated the TLR3 interacting genes and performed functional analysis on TLR3 and its interacting genes, the results demonstrated that the functions of TLR3 and its interacting genes were mainly focused on pattern recognition receptor signaling pathway, toll-like receptor signaling pathway, I-kappa B kinase/NF-kappa B signaling, positive regulation of defense response, tumor necrosis factor superfamily cytokine production, programmed necrotic cell death and activation of protein kinase activity. NF-kappa B is associated with the origin and progression of malignancies [(Jin et al., 2019); (Wang et al., 2019)]. Tumor necrosis factor has an effect on tumor-associated macrophages and cancer cells, thus affecting the prognosis of patients (Cassetta et al., 2019). The effect of programmed necrotic cell death can not only confer on tumor growth advantage, but also make tumor cells necrotic and vulnerable (Lin et al., 2020). Protein kinase activity exerts a critical role in antioxidant stress and cancer development [ (Xu et al., 2019a); (Xu et al., 2020)]. These results once again provide a theoretical basis for TLR3 to play a different prognostic value in different cancers. Encouragingly, our study found that TRAF6 was a key gene in the interactions between TLR3 and its interacting genes. TRAF6, a key inflammatory mediator, mediates tumor growth and metastasis and effectively reduces the migration and activation of macrophages [(Park et al., 2020); (Seijkens et al., 2018)]. Additionally, TRAF6 plays an important role in the activation of TLR3 (Jiang et al., 2003). Studies have reported that immune checkpoint proteins affect tumor occurrence and development by regulating TRAF6, regulating toll-like receptor signal pathway and stimulating tumor immune microenvironment (Xu et al., 2019b). These results comprehensively demonstrated the important role of TLR3 in tumor immune microenvironment.

Previous studies on TLR3 were mainly focused on infectious diseases and inflammation-related diseases, but there were few studies on cancers. Our study enriched the associations between TLR3 and cancers and proved that TLR3 had the potential to be a diagnostic and prognostic biomarker for KIRC, LGG and PAAD, especially an early diagnosis biomarker of KIRC. However, the role and molecular mechanisms of TLR3 in cancer are quite complex, but there were significant correlations between the expression and SCNA of TLR3 and levels of immune cells infiltration of KIRC, LGG and PAAD. Moreover, GSEA of TLR3 in KIRC, LGG and PAAD showed that TLR3 was closely related to tumor immune microenvironment. The PPI network and functional analysis of TLR3 and its interacting genes also proved the close relationship between TLR3 and tumor immune microenvironment. We have every reason to speculate that TLR3 affects the prognosis of KIRC, LGG and PAAD by affecting tumor immune microenvironment, although TLR3 is also likely to influence the prognosis of patients through other factors. On the other hand, it is undeniable that our study still has some limitations. For example, we did not verify the association of PAAD and LGG with TLR3, and the sample size of comparing the expression levels of TLR3 protein in plasma of early KIRC patients and healthy adults needs to be further increased. The expression of TLR3 mRNA in early KIRC and adjacent normal tissues also needs to be further verified. More experimental and clinical researches are necessary to determine our findings about the effects of TLR3 on KIRC, LGG and PAAD. Next, we will collect blood and tissue samples from KIRC, LGG, and PAAD patients. With the continuous collection of samples, we will increase the sample size to verify our results in future studies.

## 5 Conclusion

Compared with the corresponding normal tissues, TLR3 expression levels were significantly increased in KIRC, LGG and PAAD. Moreover, the expression of TLR3 was significantly higher in early KIRC compared with that in paracancerous nontumor tissues. The occurrence of KIRC could directly lead to the increase of TLR3 protein in plasma. Elevated expression of TLR3 was beneficial to prolong the OS, DFS, PFI and DSS of KIRC. Suppressed expression of TLR3 was beneficial to prolong the OS, DFS, PFI and DSS of LGG. Suppressed expression of TLR3 was beneficial for prolonging OS and DFI of PAAD. The expression and function of TLR3 in KIRC, LGG and PAAD were closely related to tumor immune microenvironment. TRAF6 was a key gene in the interactions between TLR3 and its interacting genes. Considering the changes of TLR3 expression levels in KIRC, LGG and PAAD, as well as the influence of TLR3 expression on the prognosis of these three cancers, and the close correlation between TLR3 and the tumor immune microenvironment, TLR3 may become a potential therapeutic target and an important molecular biomarker for judging the prognosis of KIRC, LGG and PAAD, as well as a diagnostic biomarker of KIRC, LGG and PAAD, especially an early diagnostic biomarker of KIRC.

## Data Availability

The original contributions presented in the study are included in the article/Supplementary Material, further inquiries can be directed to the corresponding author.
