# Supplementary material for: TLR3 serves as a novel diagnostic and prognostic biomarker and is closely correlated with immune microenvironment in three types of cancer
Source: Front Genet. 2022 Nov 7;13:905988. doi: 10.3389/fgene.2022.905988 (PMC9676367; doi:10.3389/fgene.2022.905988)
Supplement: Supplementary file 1 [file DataSheet1.ZIP › all raw data/Ethics approval.pdf]

广西医科大学第一附属医院
THE FIRST AFFILIATED HOSPITAL OF
GUANGXI MEDICAL UNIVERSITY

FIRST AFFILIATED HOSPITAL of GUANGXI MEDICAL UNIVERSITY
ETHICAL REVIEW COMMITTEE
Approval Notice

Approval Number: 2021(KY-E-182)

Title: Toll-like Receptors Serve as Early Diagnostic Biomarkers and Therapeutic Targets for Kidney Renal Clear Cell Carcinoma by Influencing the Immune Microenvironment： Comprehensive Bioinformatics Analysis Combined with Experimental Validation

Research Contents: We systematically investigated the expressions of TLRs in KIRC by ONCOMINE, UALCAN and GEPIA databases, and analyzed the relationships between the expressions of TLRs and tumor stage and prognosis in patients with KIRC by GEPIA. we used clinical samples to verify the expressions of TLR3 and TLR4 in early stage of KIRC by RT-qPCR and flow cytometry (FC). Our study provides new insights into TLRs and their relationships with KIRC, contributing to the research of early diagnosis and therapeutic targets of KIRC.

Applicant: Zengnan Mo

Application Department: Department of Urology, The First Affiliated Hospital of Guangxi Medical University, 530021 Nanning, Guangxi, China

Acknowledgement: This work was supported by the grants from the National Natural Science Foundation of China (81770759), the National Key Research and Development Program of China (2017YFC0908000), Major Project of Guangxi Innovation Driven (AA18118016), Guangxi key Laboratory for Genomic and Personalized Medicine (grant number 16-380-54, 17-259-45, 19-050-22, 19-185-33, 20-065-33), Guangxi Science and Technology Base and Talent Project (2019AC17009) and Guangxi Clinical Research Center for Urology and Nephrology (2020AC03006).

Date of Application: December 28, 2020

Date of Approval: December 29, 2020

Conclusion: This project fully considered and protected the rights and interests of the study objects. It meets the criteria of Ethical Review Committee. The Medical Ethics Committee of First Affiliated Hospital of Guangxi Medical University has approved the protocol.

Signature: Songqing He

Songqing He

(Vice) Director of Ethical Review Committee

First Affiliated Hospital of Guangxi Medical University

Date: Dec 29. 2020
