# Supplementary material for: TLR3 serves as a novel diagnostic and prognostic biomarker and is closely correlated with immune microenvironment in three types of cancer
Source: Front Genet. 2022 Nov 7;13:905988. doi: 10.3389/fgene.2022.905988 (PMC9676367; doi:10.3389/fgene.2022.905988)
Supplement: Supplementary file 1 [file DataSheet1.ZIP › all raw data/original figures/Figure 8/Figure 8B.pdf]

GeneMANIA report

Created on : 10 June 2021 20:46:41
Last database update : 13 April 2021 00:00:00
Application version : 3.6.0

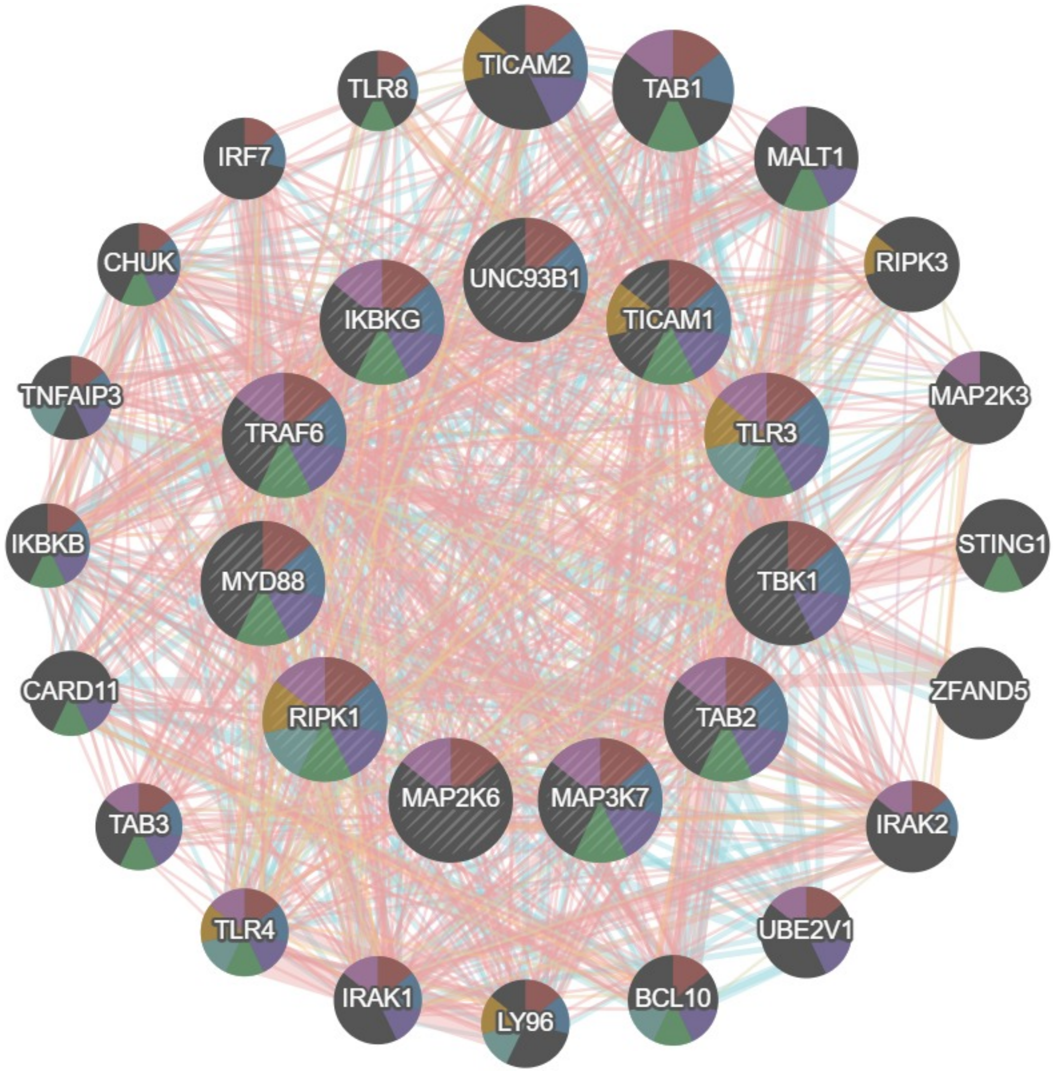

Networks

- Physical Interactions
- Pathway
- Co-expression
- Predicted
- Shared protein domains
- Co-localization

Functions

- pattern recognition receptor signaling pathway
- toll-like receptor signaling pathway
- I-kappaB kinase/NF-kappaB signaling
- positive regulation of defense response
- tumor necrosis factor superfamily cytokine production
- programmed necrotic cell death
- activation of protein kinase activity
