# Supplementary material for: TLR3 serves as a novel diagnostic and prognostic biomarker and is closely correlated with immune microenvironment in three types of cancer
Source: Front Genet. 2022 Nov 7;13:905988. doi: 10.3389/fgene.2022.905988 (PMC9676367; doi:10.3389/fgene.2022.905988)
Supplement: Supplementary file 1 [file DataSheet1.ZIP › all raw data/Supplementary table 1.docx]

The basic information of KIRC patients and healthy adults for ELISA

| Samples | Sex | Years of age | Tumor location | TNM stage | Histological type |
| --- | --- | --- | --- | --- | --- |
| Sample 1 | Male | 54 | Left | T_1_N_0_M_0_ | KIRC |
| Sample 2 | Female | 50 | Right | T_2_N_0_M_0_ | KIRC |
| Sample 3 | Female | 70 | Left | T_2_N_0_M_0_ | KIRC |
| Sample 4 | Male | 53 | Right | T_1_N_0_M_0_ | KIRC |
| Sample 5 | Female | 48 | Right | T_1_N_0_M_0_ | KIRC |
| Sample 6 | Male | 60 | Right | T_2_N_0_M_0_ | KIRC |
| Sample 7 | Male | 33 | Left | T_1_N_0_M_0_ | KIRC |
| Control 1 | Male | 42 | **/** | **/** | **/** |
| Control 2 | Male | 29 | **/** | **/** | **/** |
| Control 3 | Male | 36 | **/** | **/** | **/** |
| Control 4 | Male | 48 | **/** | **/** | **/** |
| Control 5 | Male | 50 | **/** | **/** | **/** |
| Control 6 | Female | 35 | **/** | **/** | **/** |
| Control 7 | Female | 28 | **/** | **/** | **/** |
