# Supplementary figures and images for: TLR3 serves as a novel diagnostic and prognostic biomarker and is closely correlated with immune microenvironment in three types of cancer
Source: Front Genet. 2022 Nov 7;13:905988. doi: 10.3389/fgene.2022.905988 (PMC9676367; doi:10.3389/fgene.2022.905988)

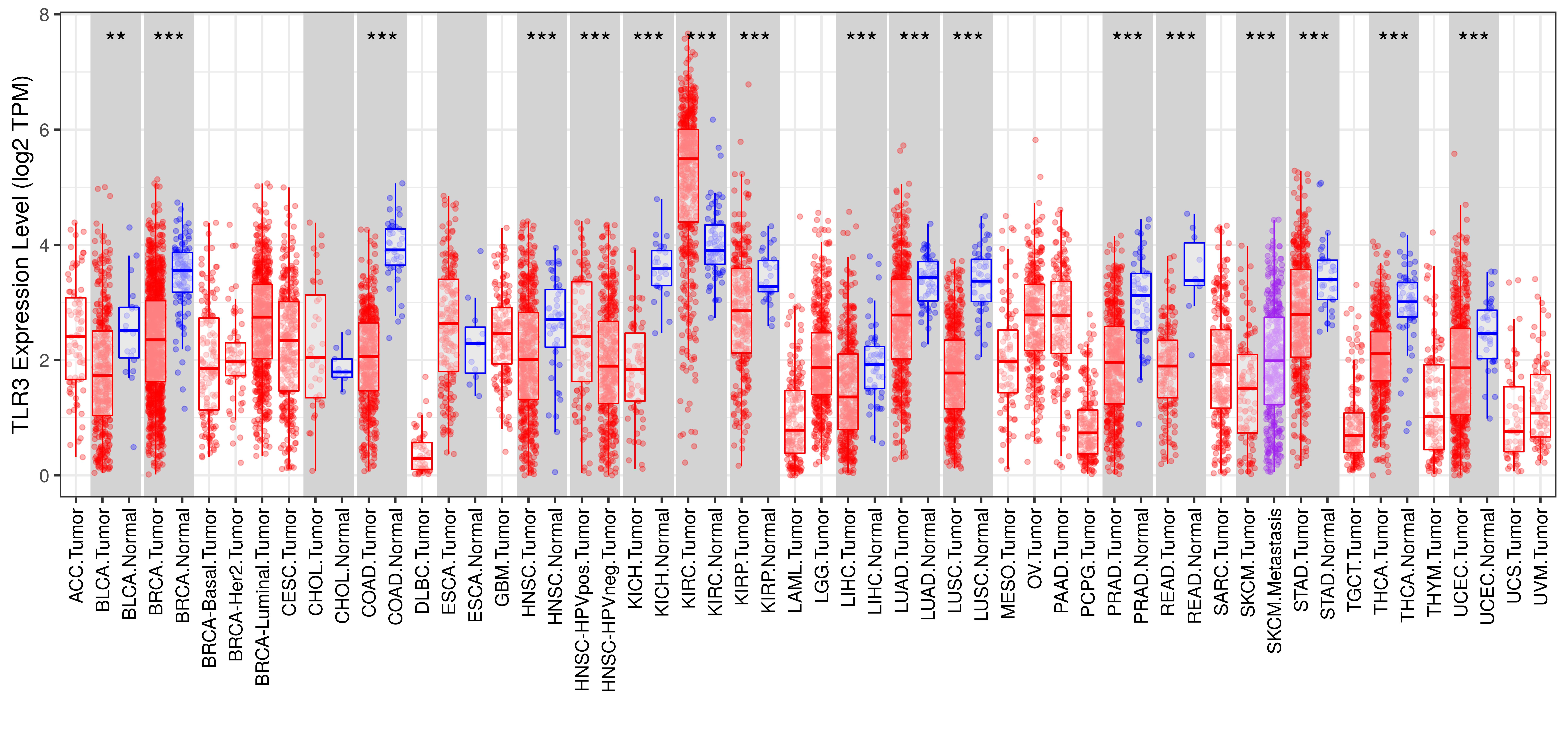

Supplement: Supplementary file 1 [file DataSheet1.ZIP › all raw data/original figures/Figure 1/Figure 1A.jpg]

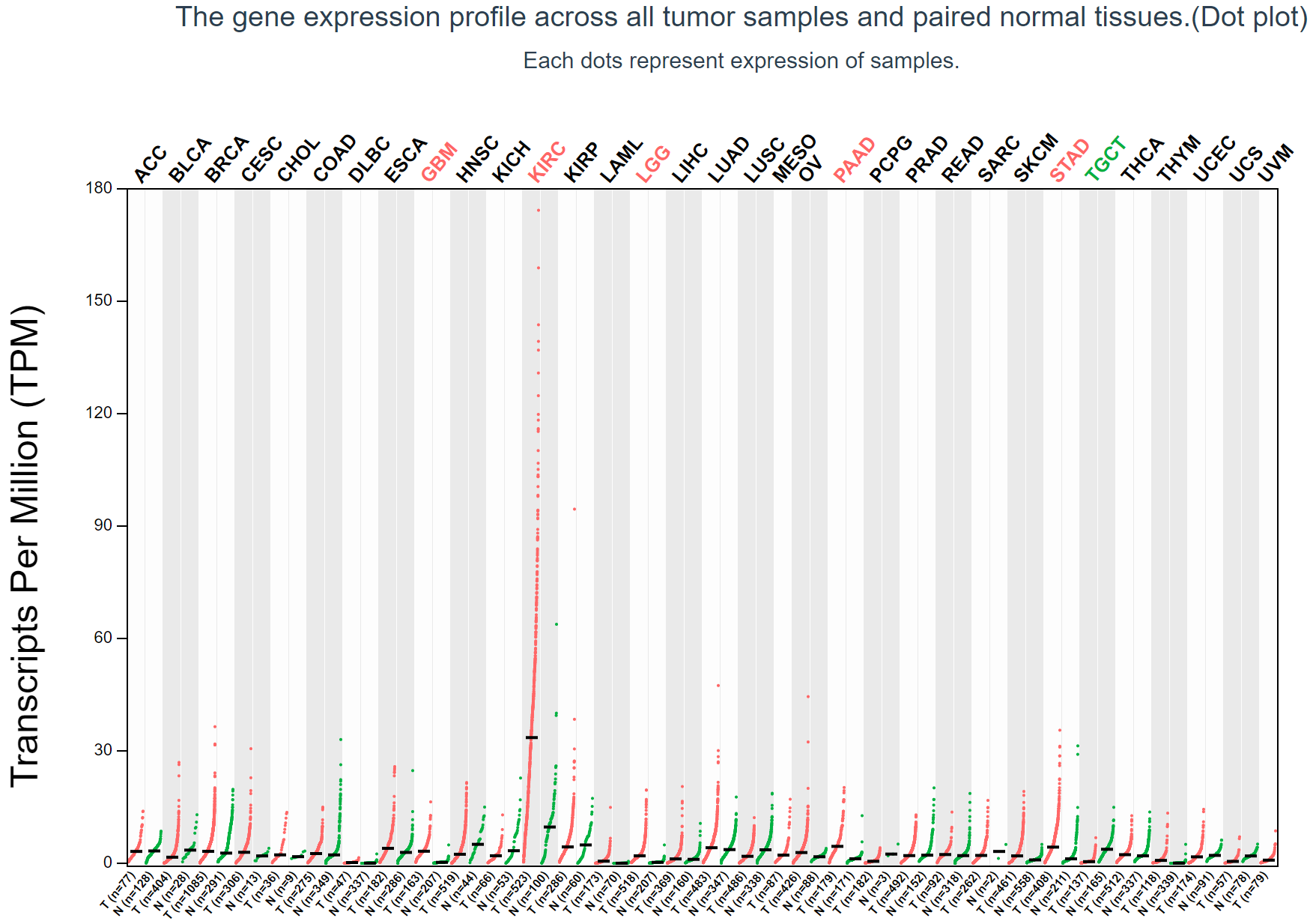

Supplement: Supplementary file 1 [file DataSheet1.ZIP › all raw data/original figures/Figure 1/Figure 1B.png]

# Overall Survival

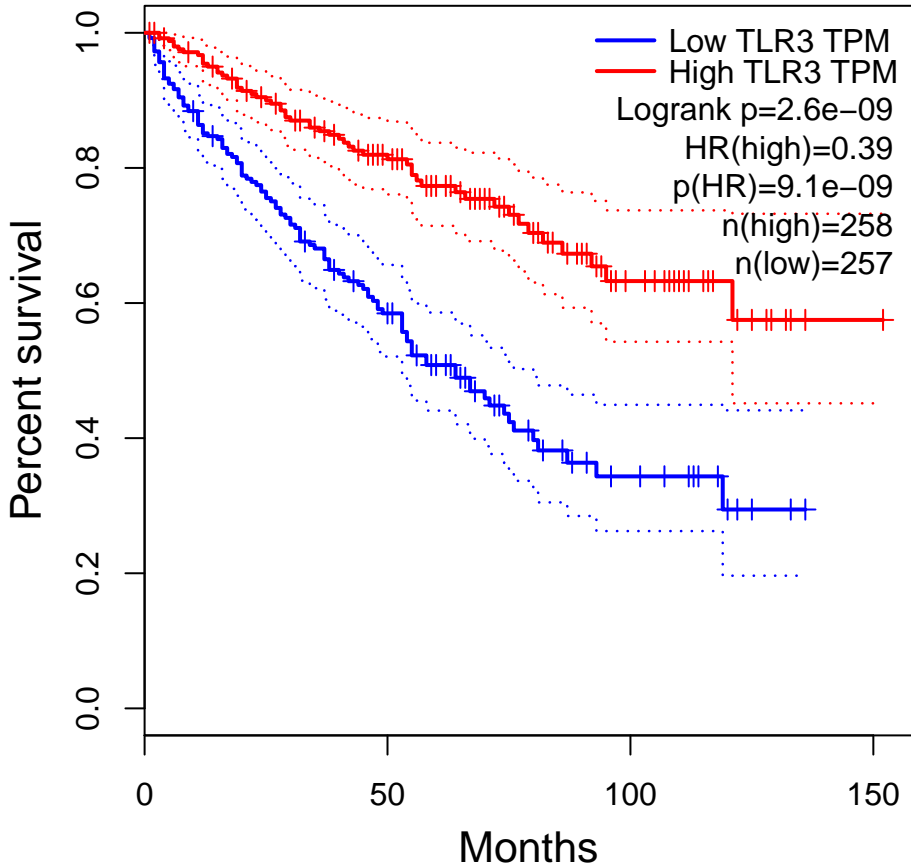

Supplement: Supplementary file 1 [file DataSheet1.ZIP › all raw data/original figures/Figure 2/Figure 2A.pdf]

# Overall Survival

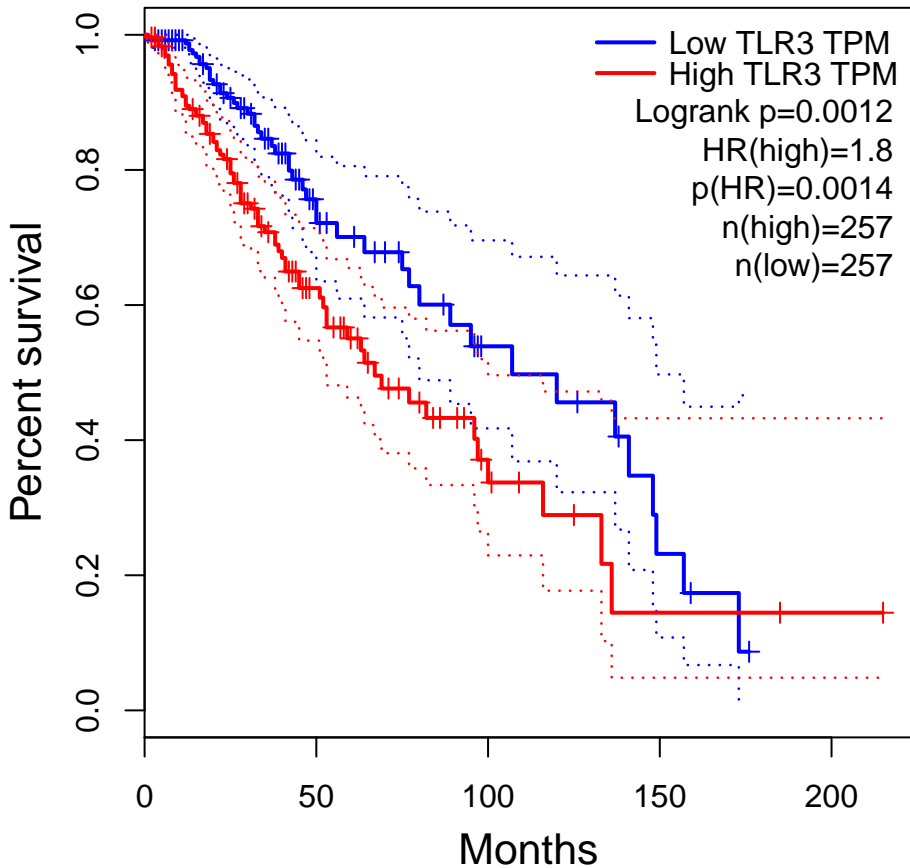

Supplement: Supplementary file 1 [file DataSheet1.ZIP › all raw data/original figures/Figure 2/Figure 2B.pdf]

# Overall Survival

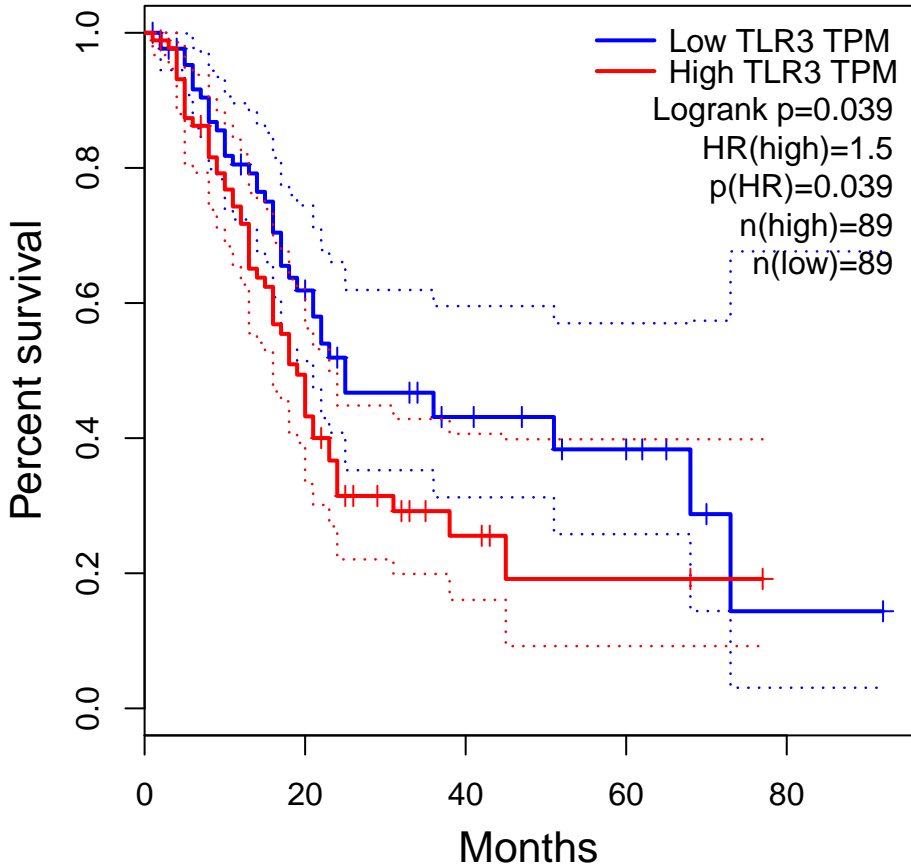

Supplement: Supplementary file 1 [file DataSheet1.ZIP › all raw data/original figures/Figure 2/Figure 2C.pdf]

# Disease Free Survival

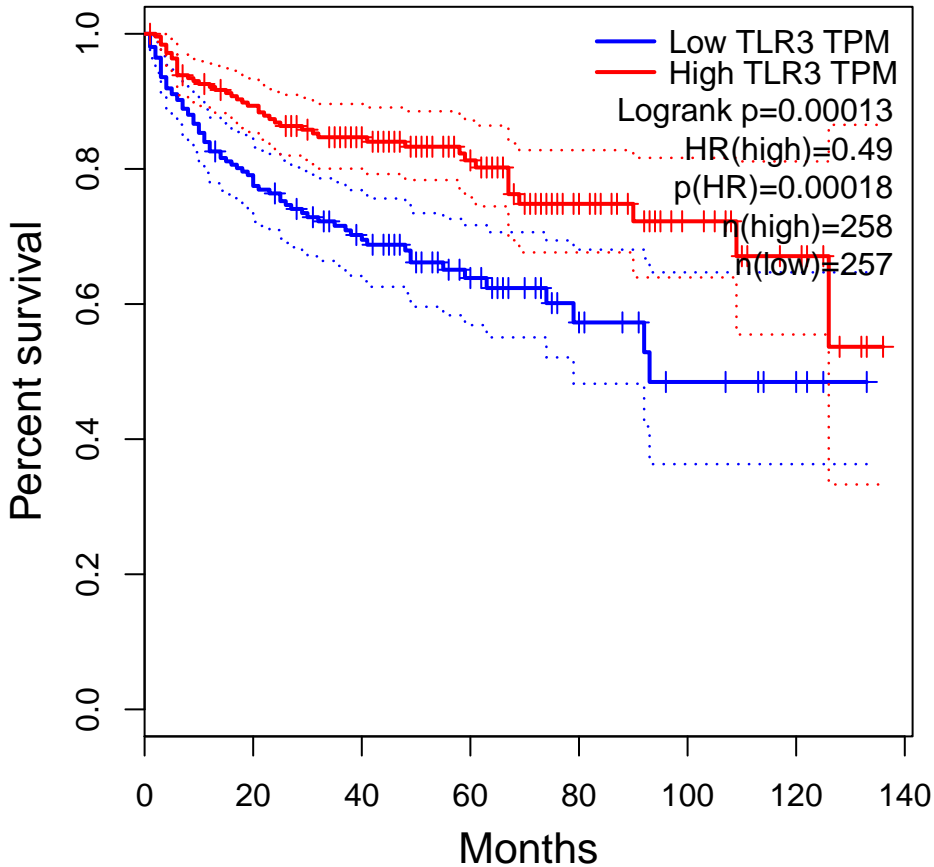

Supplement: Supplementary file 1 [file DataSheet1.ZIP › all raw data/original figures/Figure 2/Figure 2D.pdf]

# Disease Free Survival

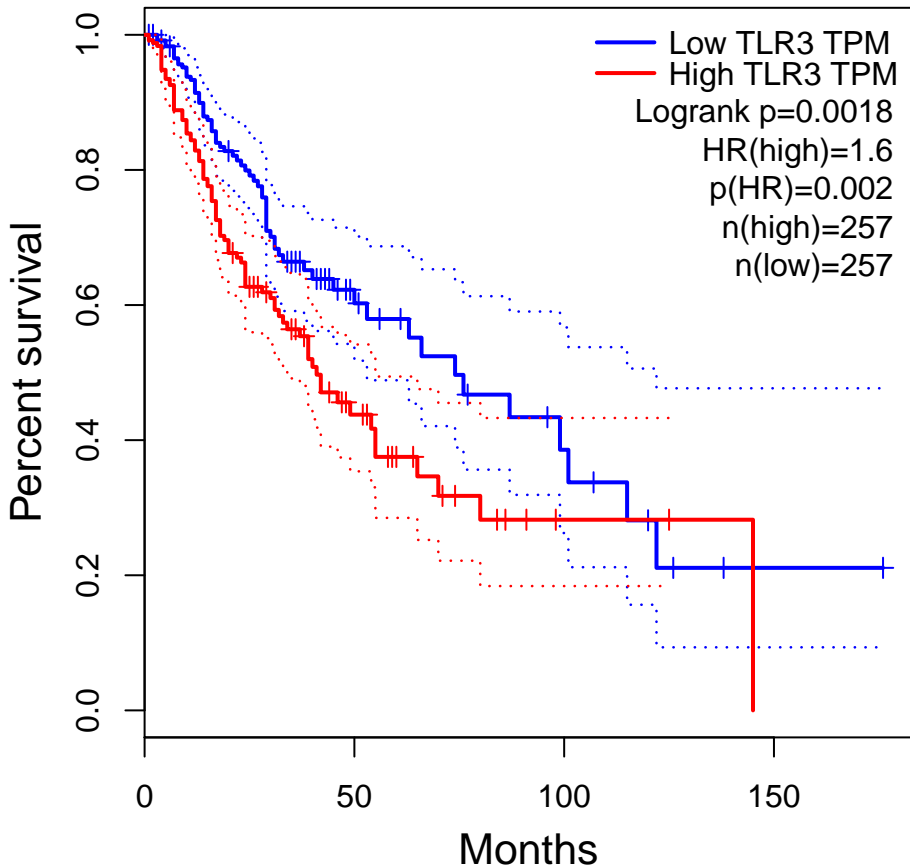

Supplement: Supplementary file 1 [file DataSheet1.ZIP › all raw data/original figures/Figure 2/Figure 2E.pdf]

# Disease Free Survival

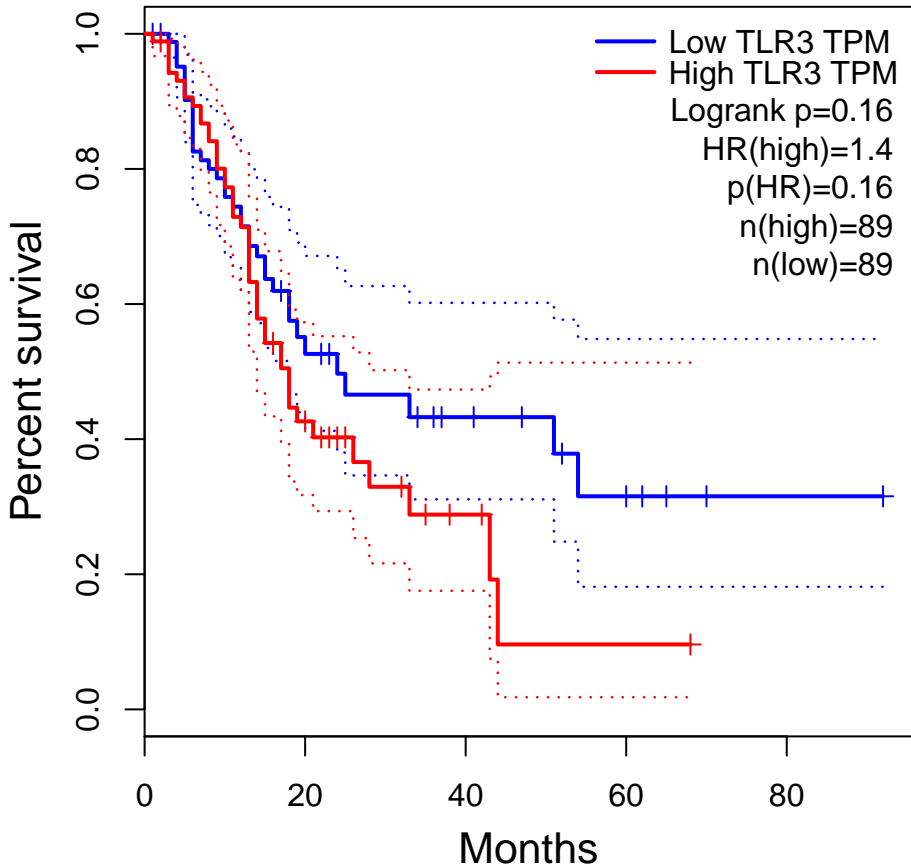

Supplement: Supplementary file 1 [file DataSheet1.ZIP › all raw data/original figures/Figure 2/Figure 2F.pdf]

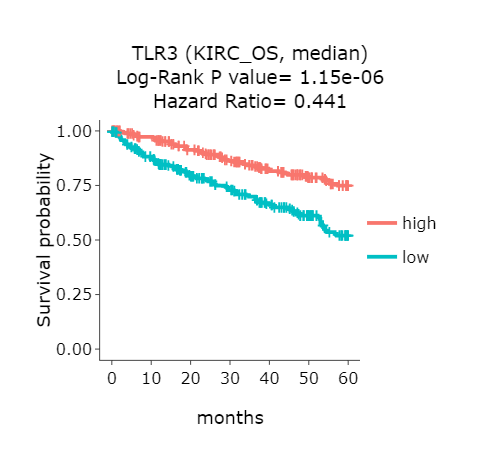

Supplement: Supplementary file 1 [file DataSheet1.ZIP › all raw data/original figures/Figure 3/Figure 3A.png]

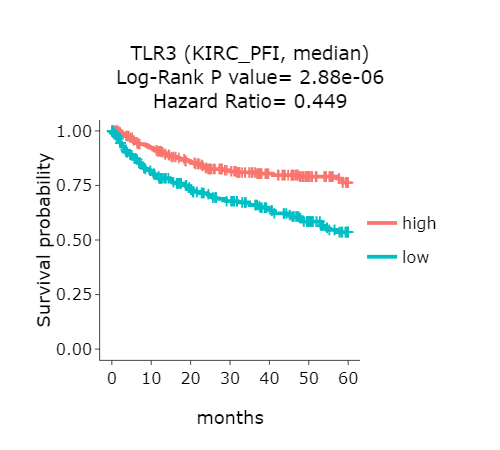

Supplement: Supplementary file 1 [file DataSheet1.ZIP › all raw data/original figures/Figure 3/Figure 3B.png]

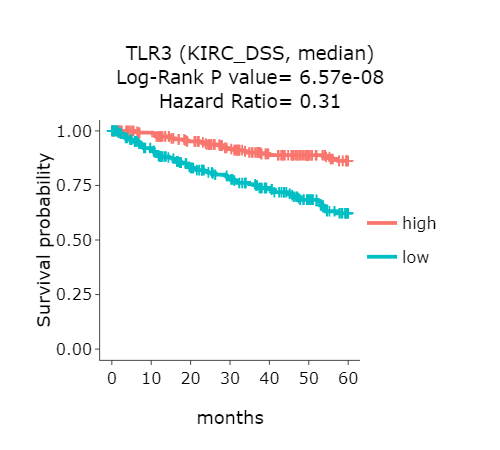

Supplement: Supplementary file 1 [file DataSheet1.ZIP › all raw data/original figures/Figure 3/Figure 3C.png]

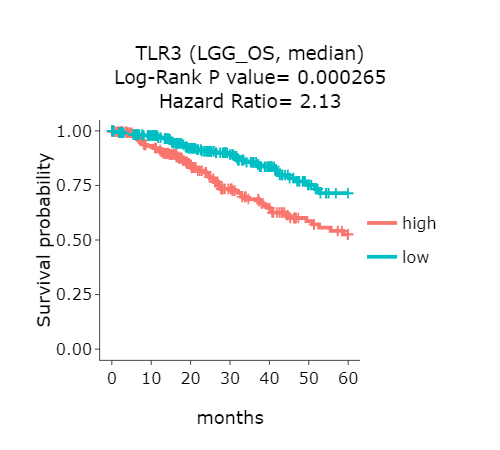

Supplement: Supplementary file 1 [file DataSheet1.ZIP › all raw data/original figures/Figure 3/Figure 3D.png]

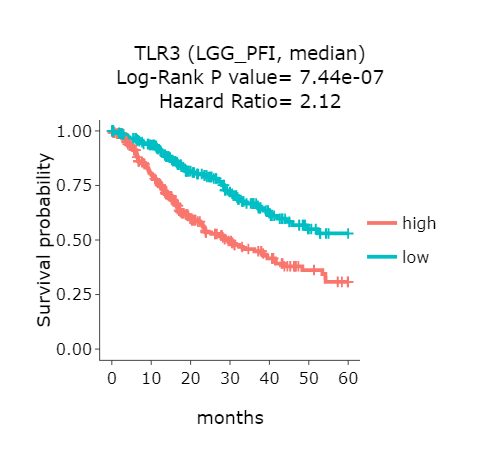

Supplement: Supplementary file 1 [file DataSheet1.ZIP › all raw data/original figures/Figure 3/Figure 3E.png]

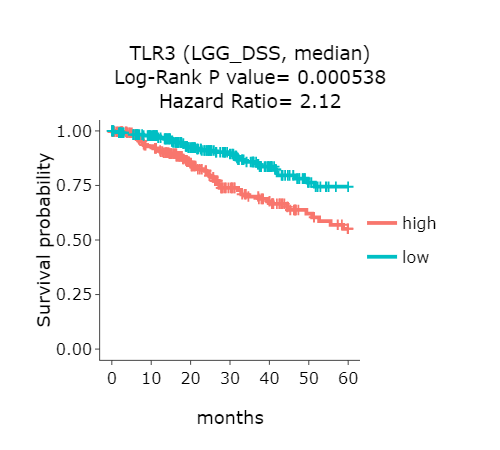

Supplement: Supplementary file 1 [file DataSheet1.ZIP › all raw data/original figures/Figure 3/Figure 3F.png]

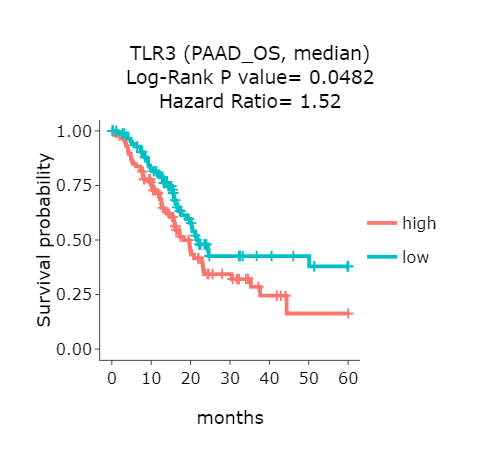

Supplement: Supplementary file 1 [file DataSheet1.ZIP › all raw data/original figures/Figure 3/Figure 3G.png]

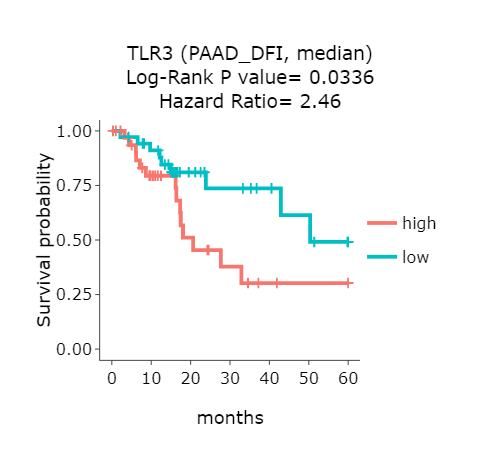

Supplement: Supplementary file 1 [file DataSheet1.ZIP › all raw data/original figures/Figure 3/Figure 3H.png]

# Expression of TLR3 in KIRC based on individual cancer stages

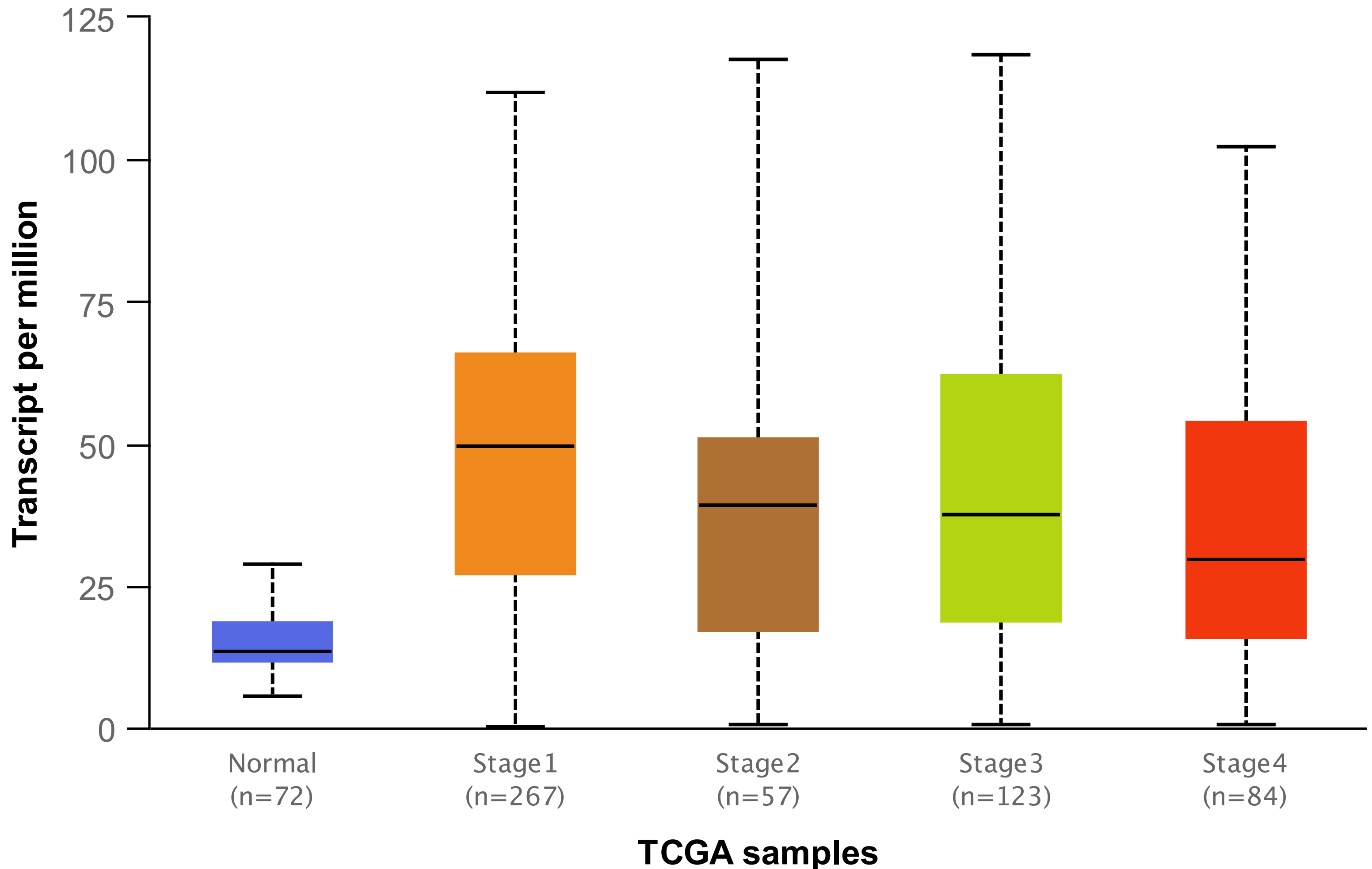

Supplement: Supplementary file 1 [file DataSheet1.ZIP › all raw data/original figures/Figure 4/Figure 4A.pdf]

# Expression of TLR3 in KIRC based on tumor grade

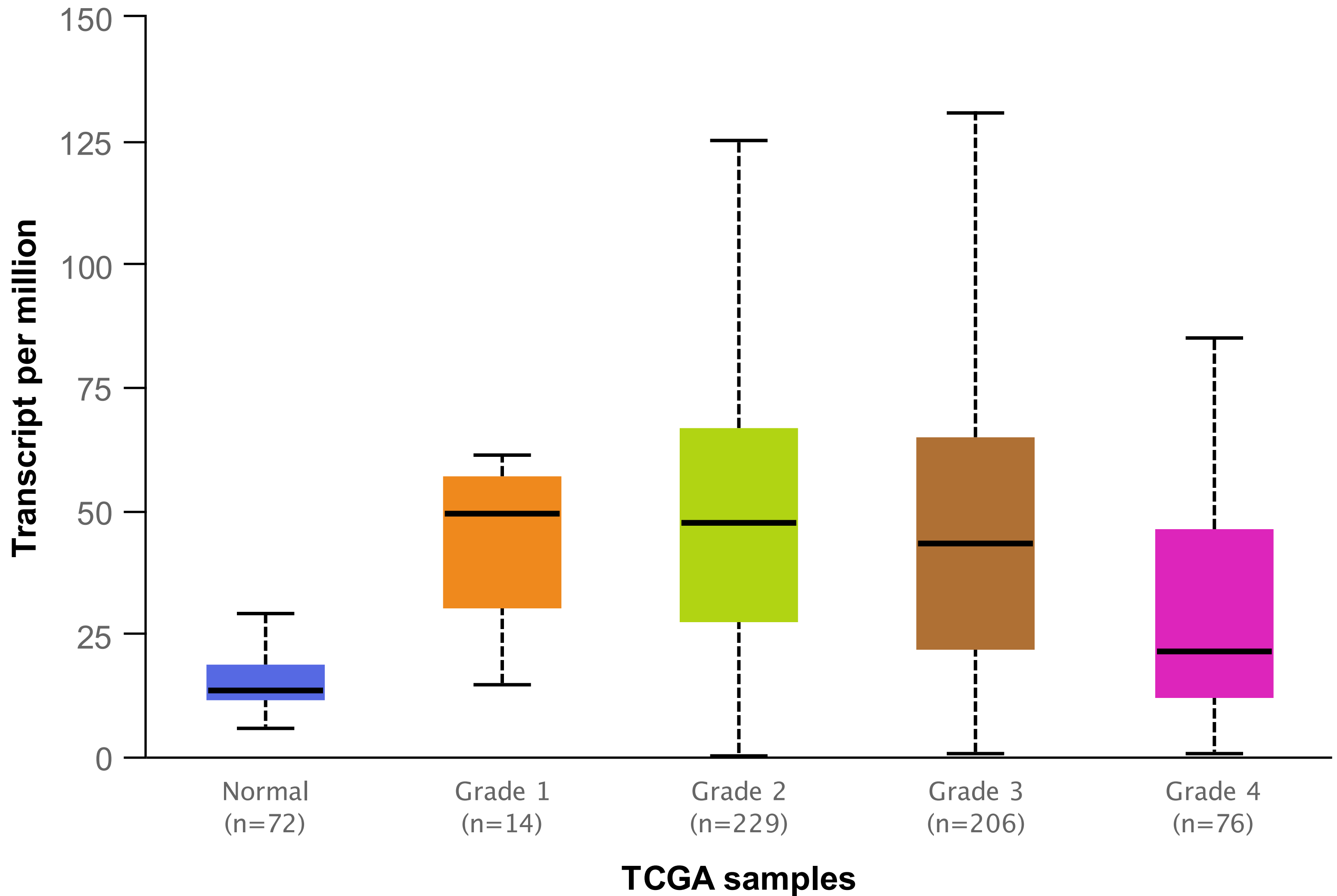

Supplement: Supplementary file 1 [file DataSheet1.ZIP › all raw data/original figures/Figure 4/Figure 4B.pdf]

# Expression of TLR3 in KIRC based on nodal metastasis status

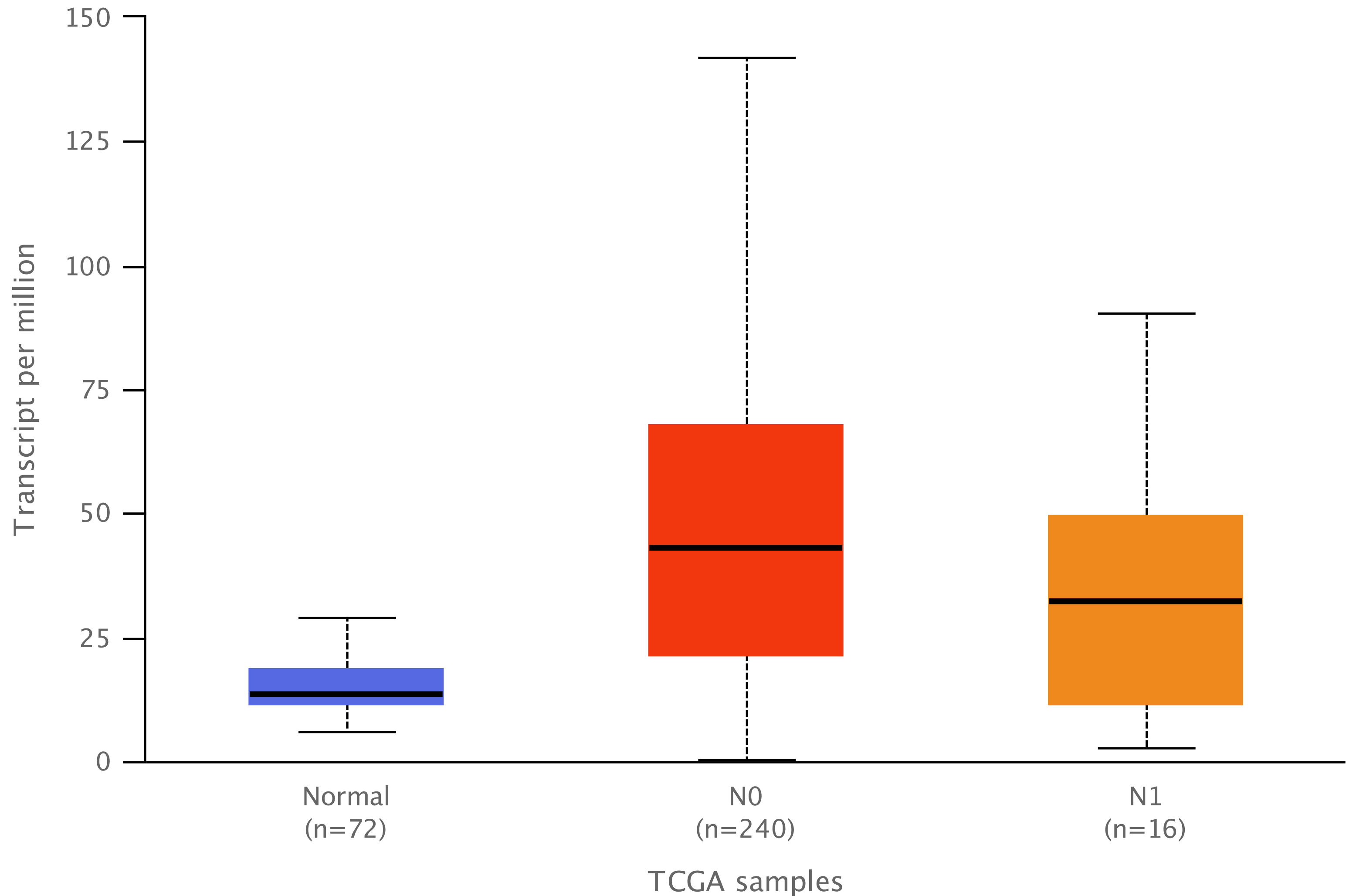

Supplement: Supplementary file 1 [file DataSheet1.ZIP › all raw data/original figures/Figure 4/Figure 4C.pdf]

# Expression of TLR3 in PAAD based on individual cancer stages

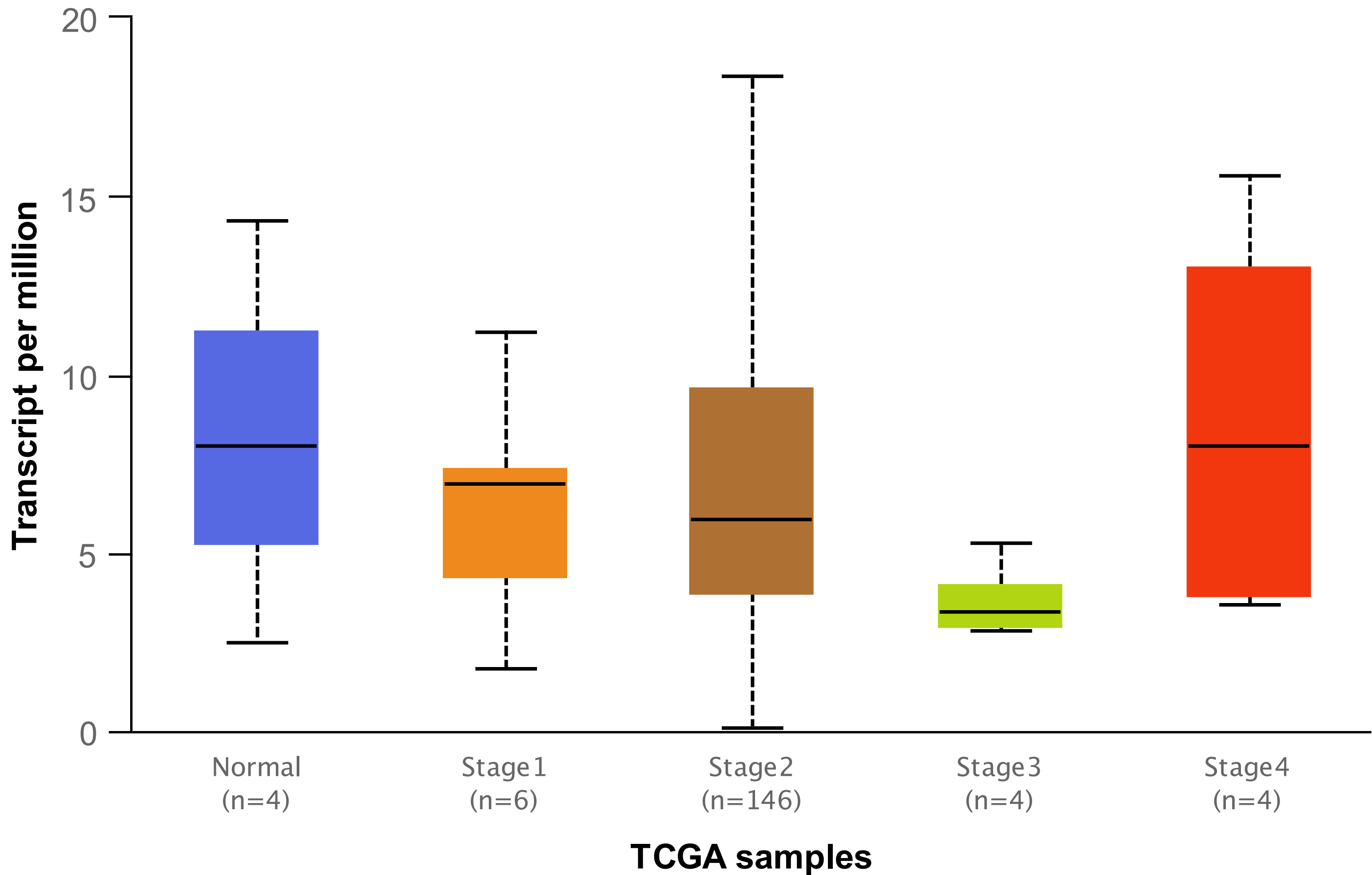

Supplement: Supplementary file 1 [file DataSheet1.ZIP › all raw data/original figures/Figure 4/Figure 4D.pdf]

# Expression of TLR3 in PAAD based on tumor grade

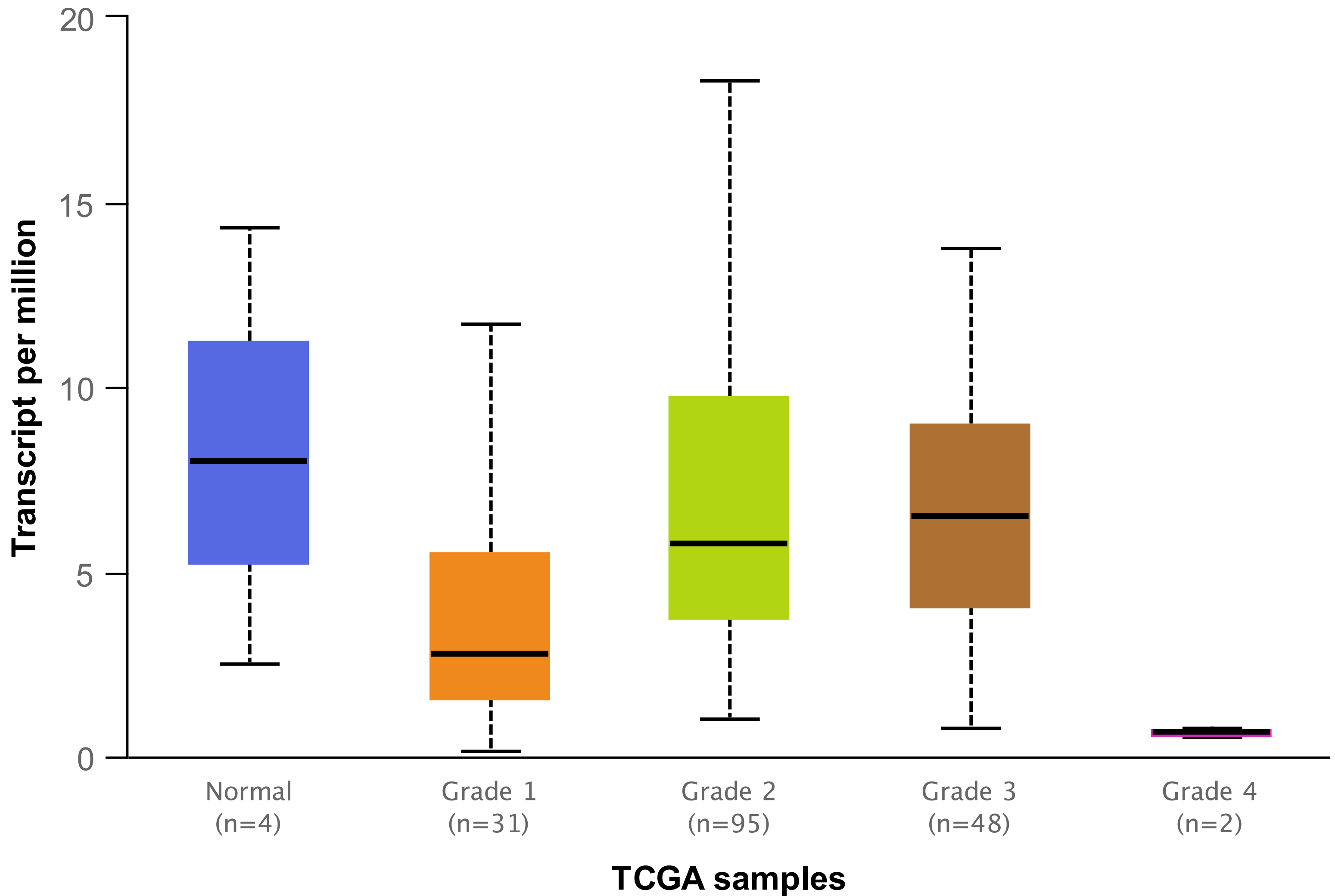

Supplement: Supplementary file 1 [file DataSheet1.ZIP › all raw data/original figures/Figure 4/Figure 4E.pdf]

# Expression of TLR3 in PAAD based on nodal metastasis status

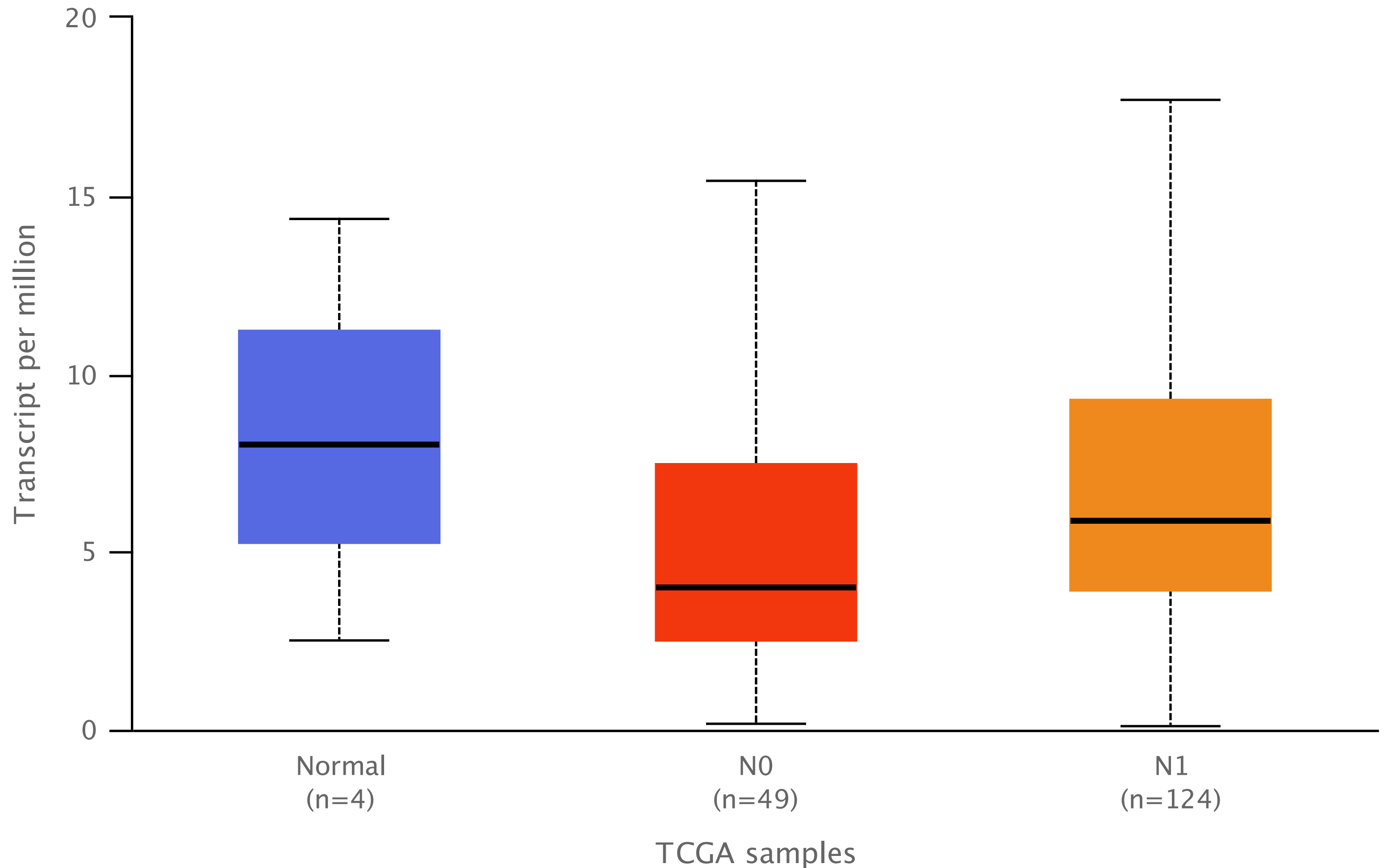

Supplement: Supplementary file 1 [file DataSheet1.ZIP › all raw data/original figures/Figure 4/Figure 4F.pdf]

# Expression of TLR3 in LGG based on tumor grade

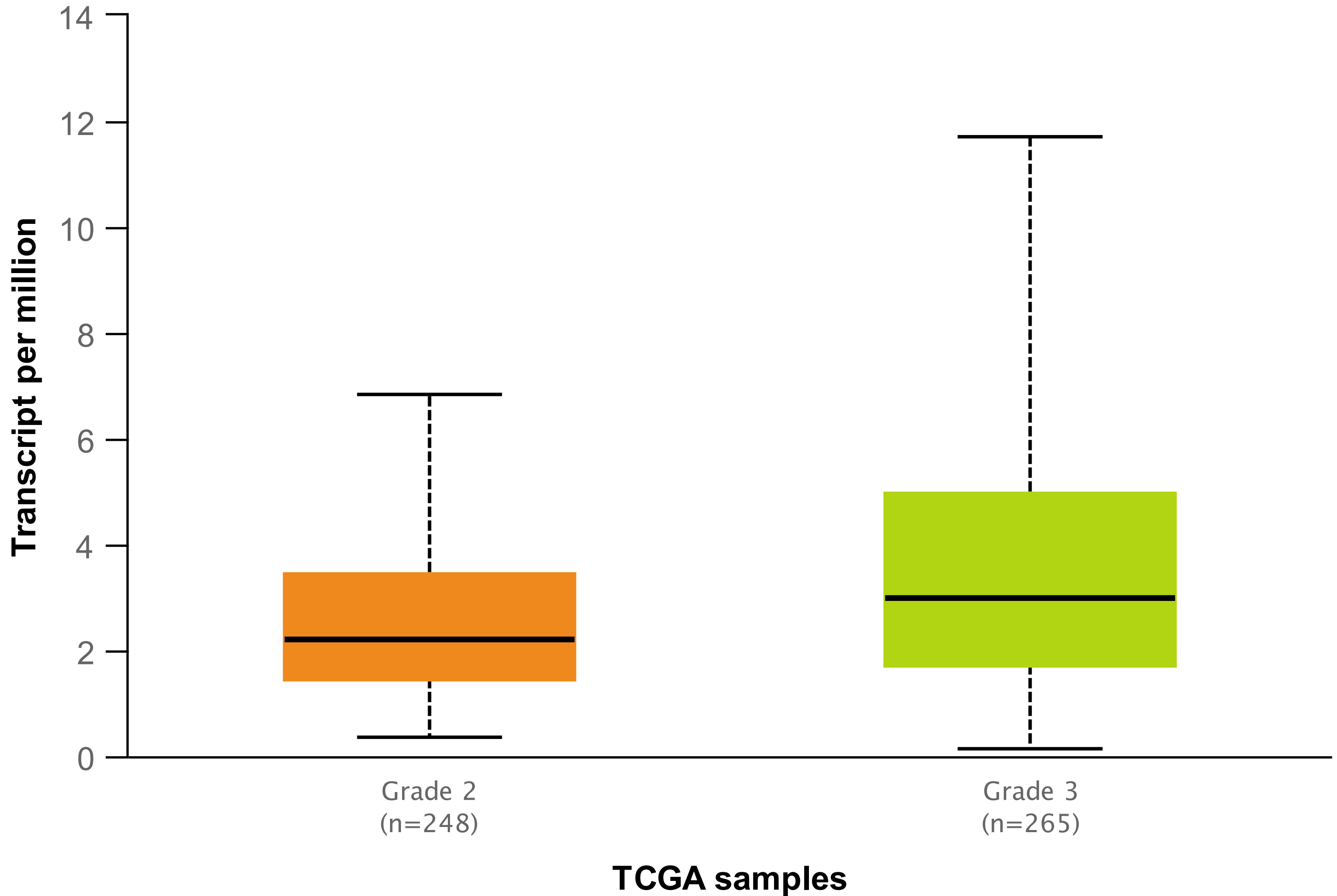

Supplement: Supplementary file 1 [file DataSheet1.ZIP › all raw data/original figures/Figure 4/Figure 4G.pdf]

# Expression of TLR3 in LGG based on histological subtypes

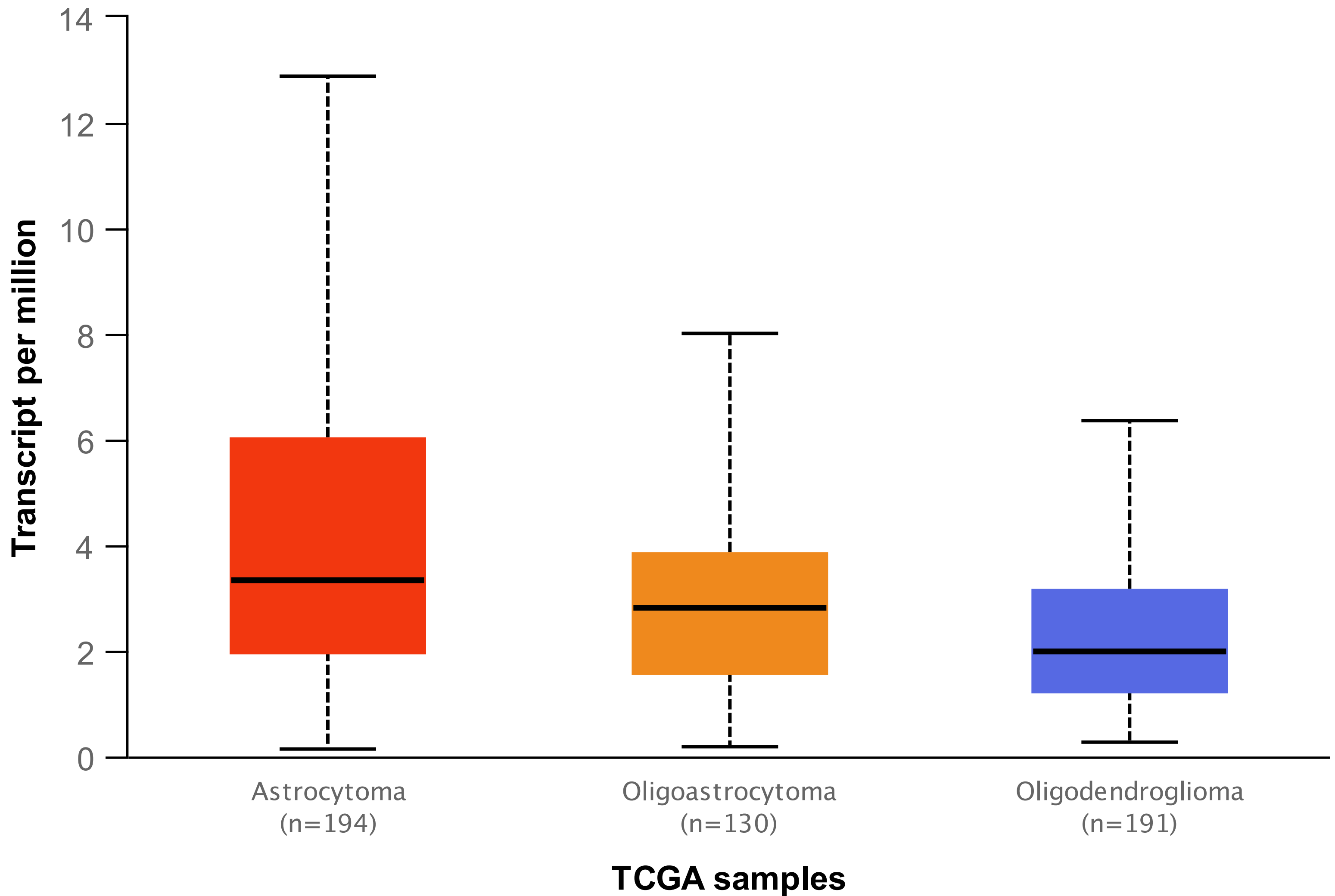

Supplement: Supplementary file 1 [file DataSheet1.ZIP › all raw data/original figures/Figure 4/Figure 4H.pdf]

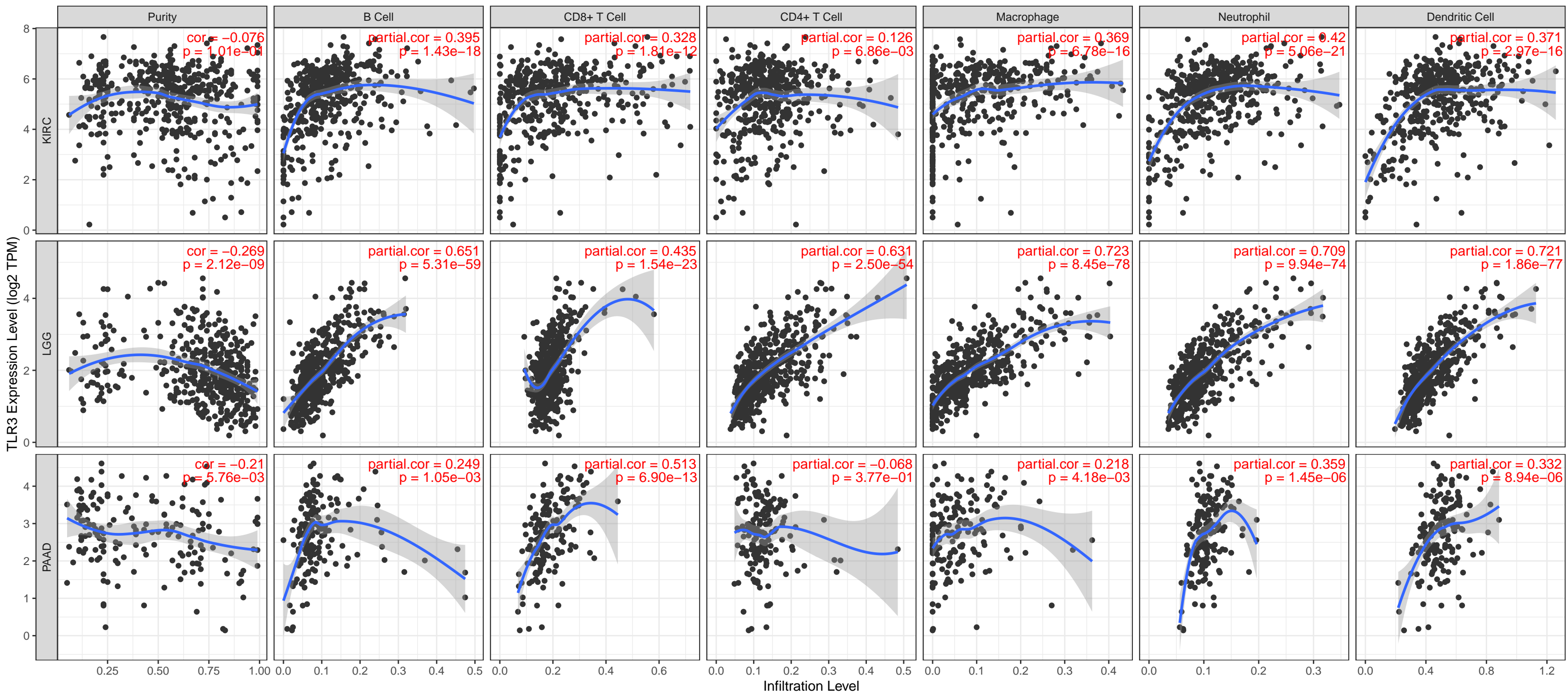

Supplement: Supplementary file 1 [file DataSheet1.ZIP › all raw data/original figures/Figure 5/Figure 5.pdf]

Infiltration Level

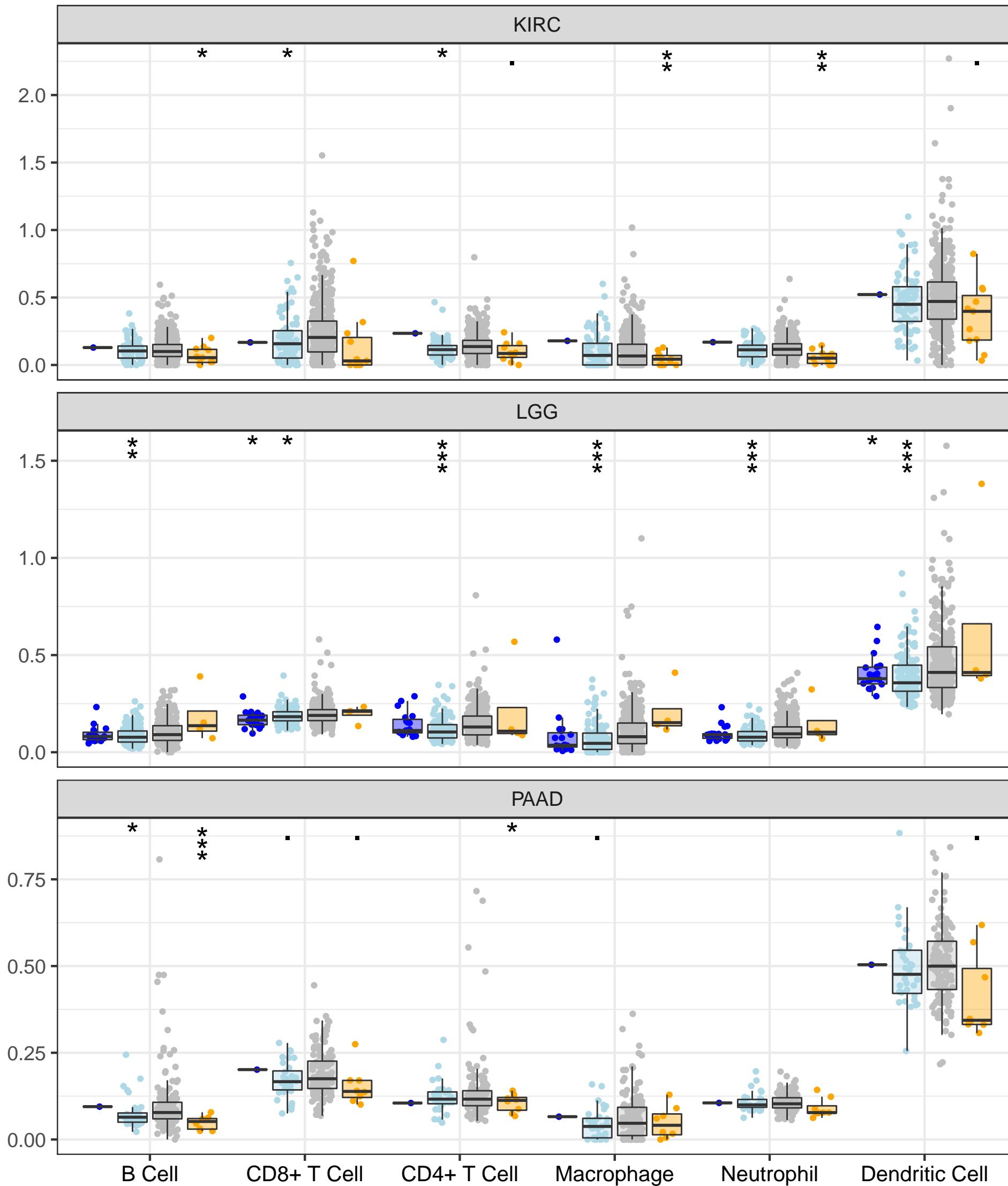

Supplement: Supplementary file 1 [file DataSheet1.ZIP › all raw data/original figures/Figure 6/Figure 6.pdf]

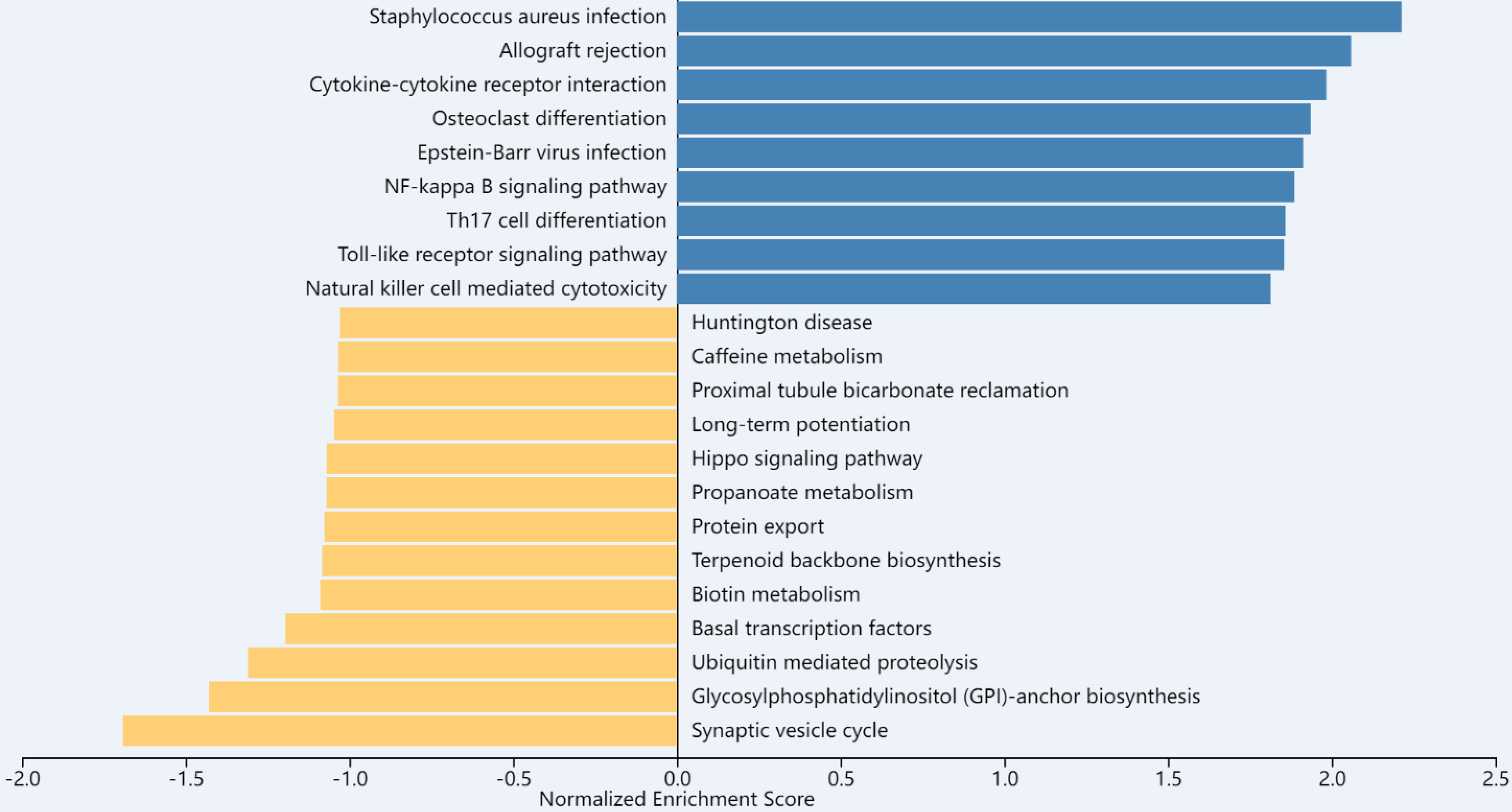

Supplement: Supplementary file 1 [file DataSheet1.ZIP › all raw data/original figures/Figure 7/Figure 7A.png]

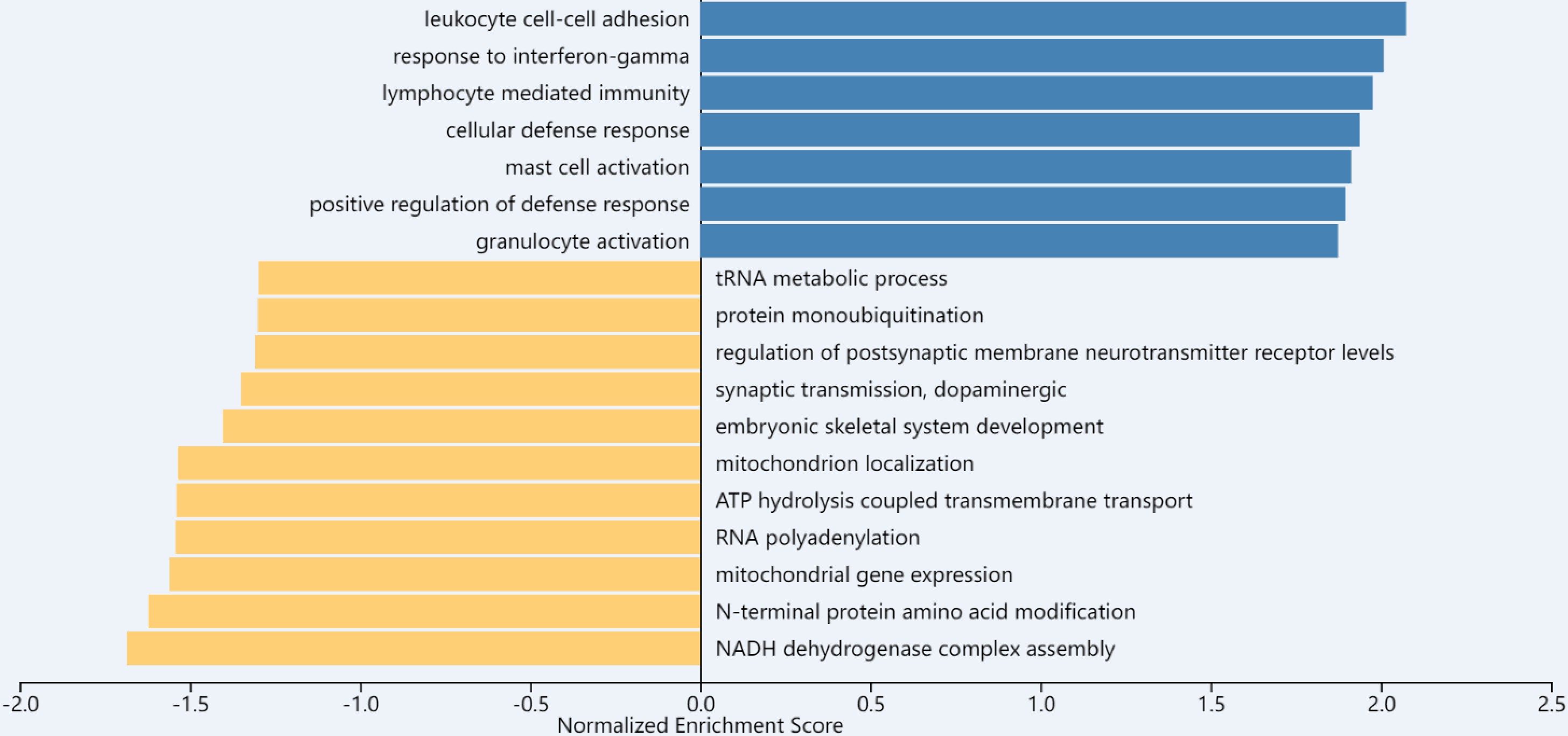

Supplement: Supplementary file 1 [file DataSheet1.ZIP › all raw data/original figures/Figure 7/Figure 7B.png]

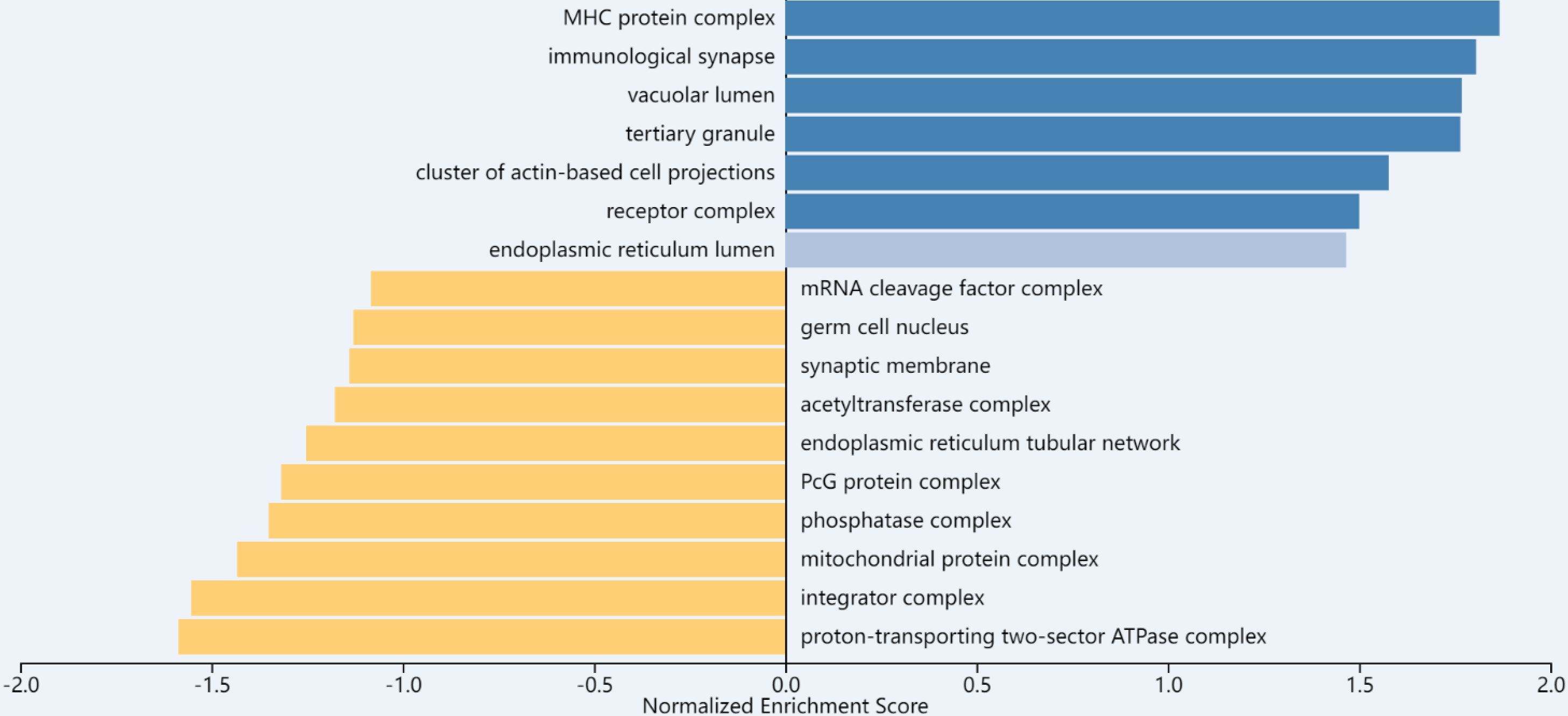

Supplement: Supplementary file 1 [file DataSheet1.ZIP › all raw data/original figures/Figure 7/Figure 7C.png]

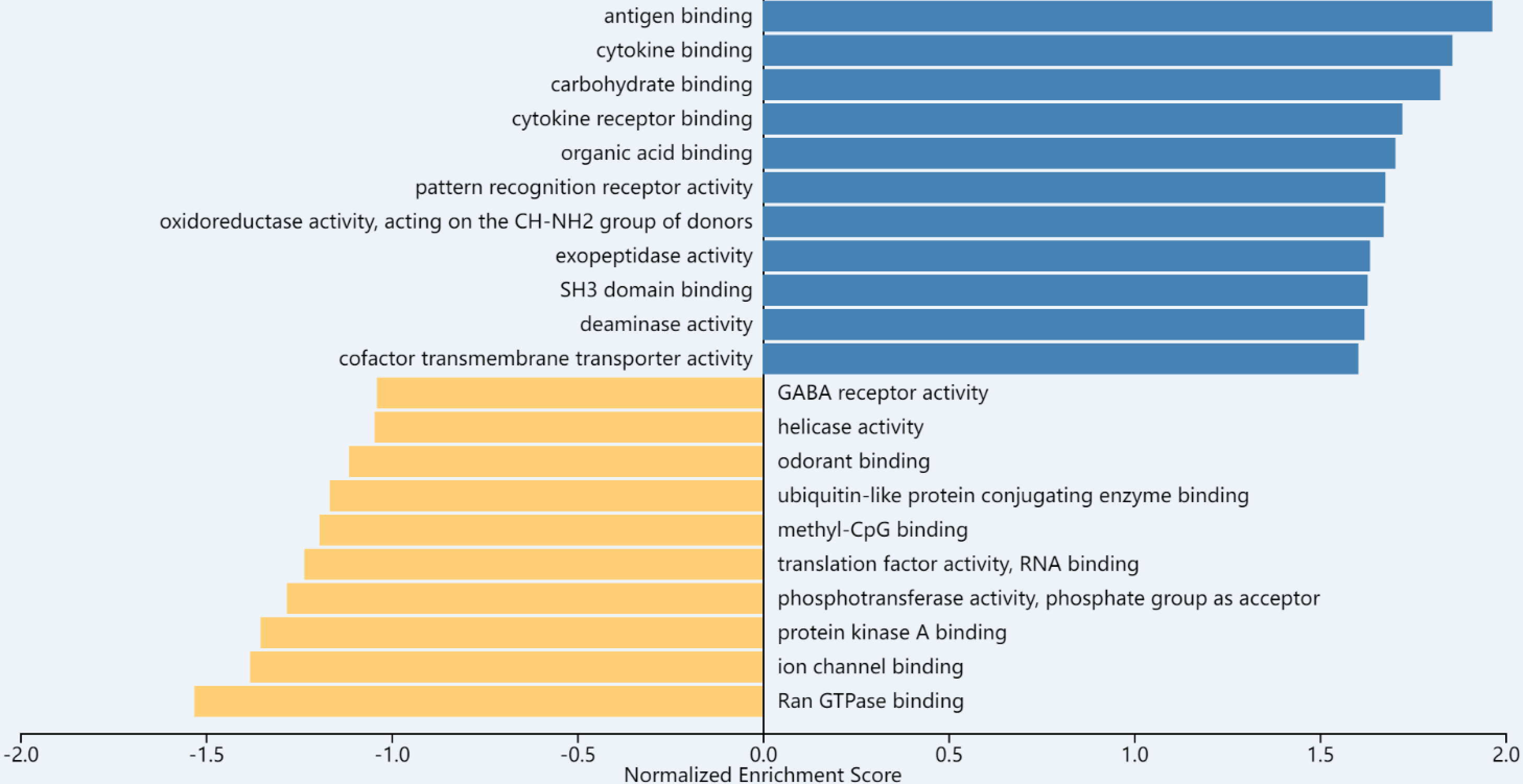

Supplement: Supplementary file 1 [file DataSheet1.ZIP › all raw data/original figures/Figure 7/Figure 7D.png]

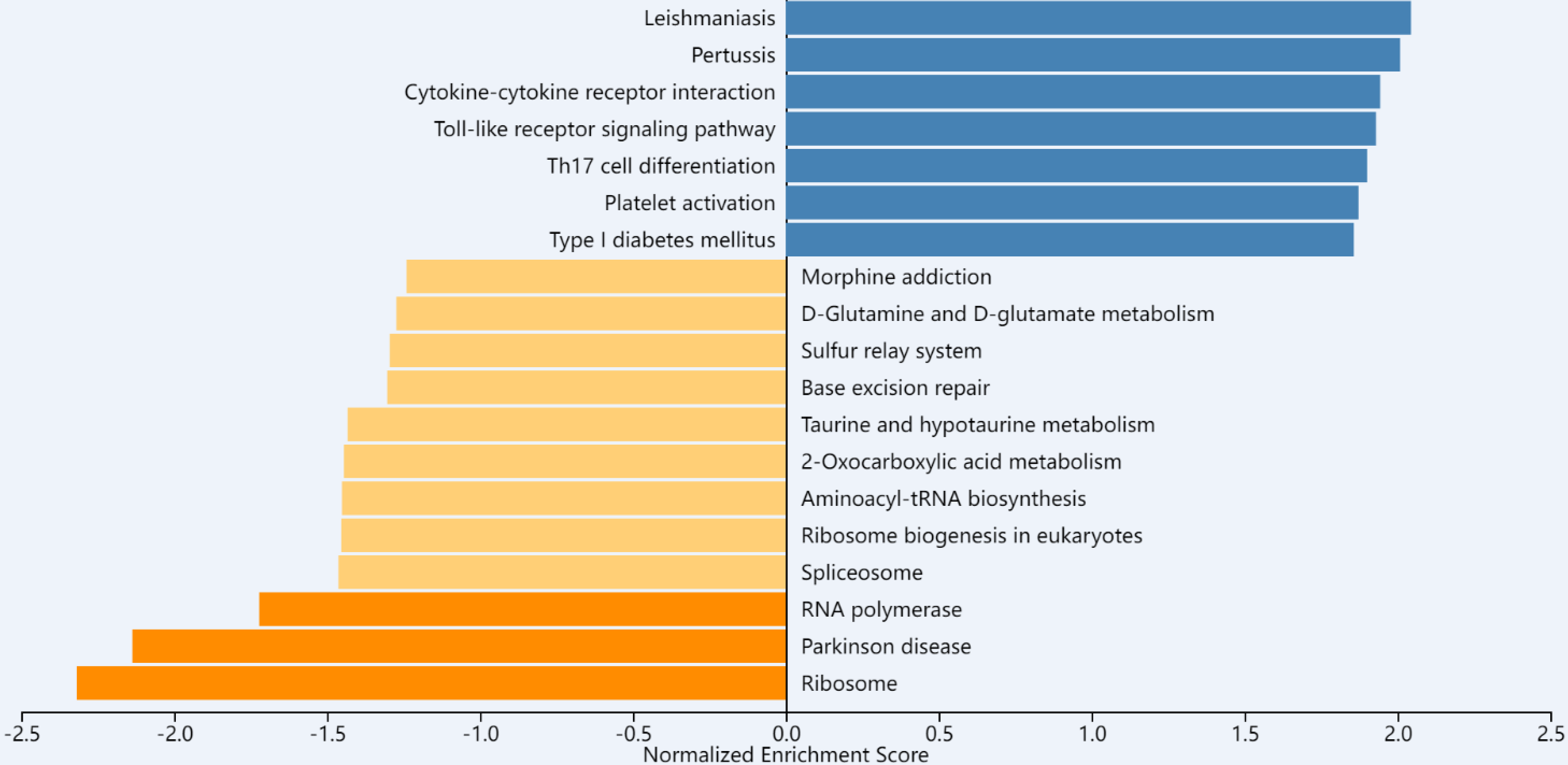

Supplement: Supplementary file 1 [file DataSheet1.ZIP › all raw data/original figures/Figure 7/Figure 7E.png]

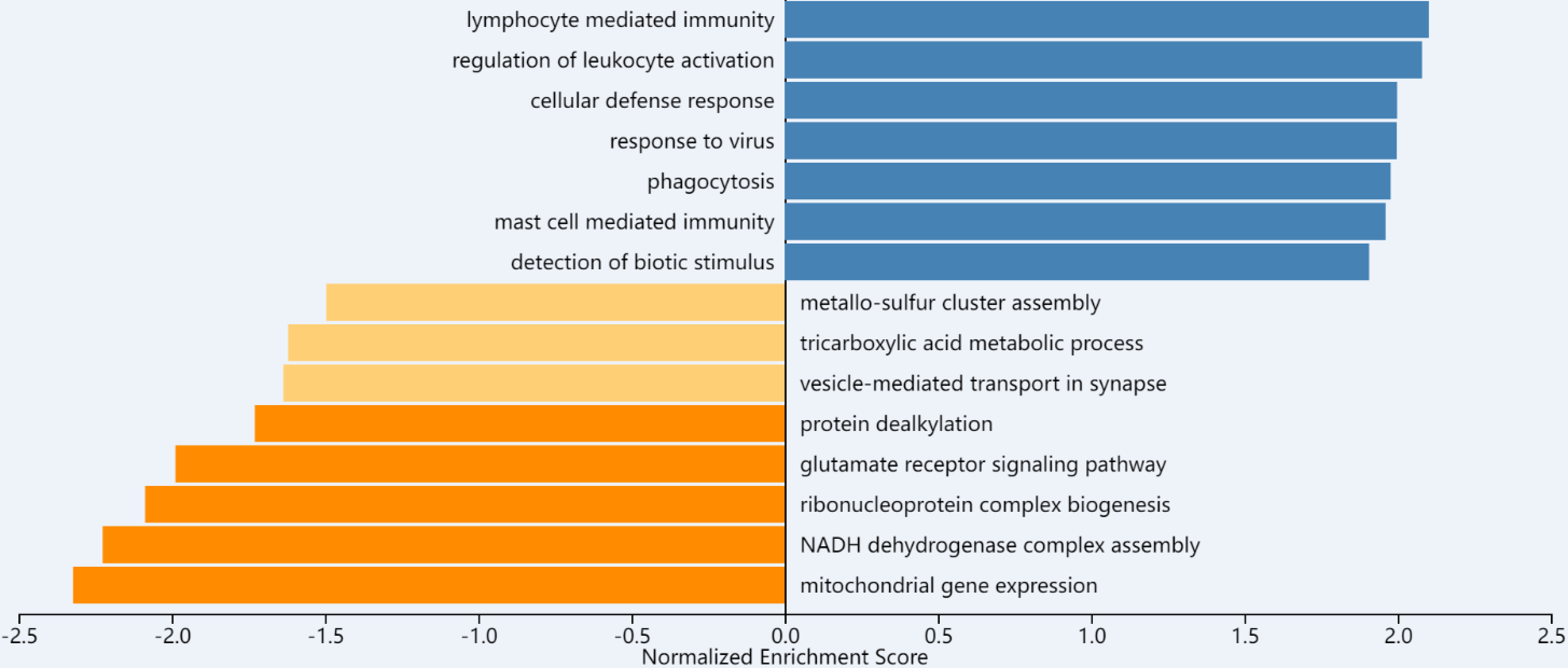

Supplement: Supplementary file 1 [file DataSheet1.ZIP › all raw data/original figures/Figure 7/Figure 7F.png]

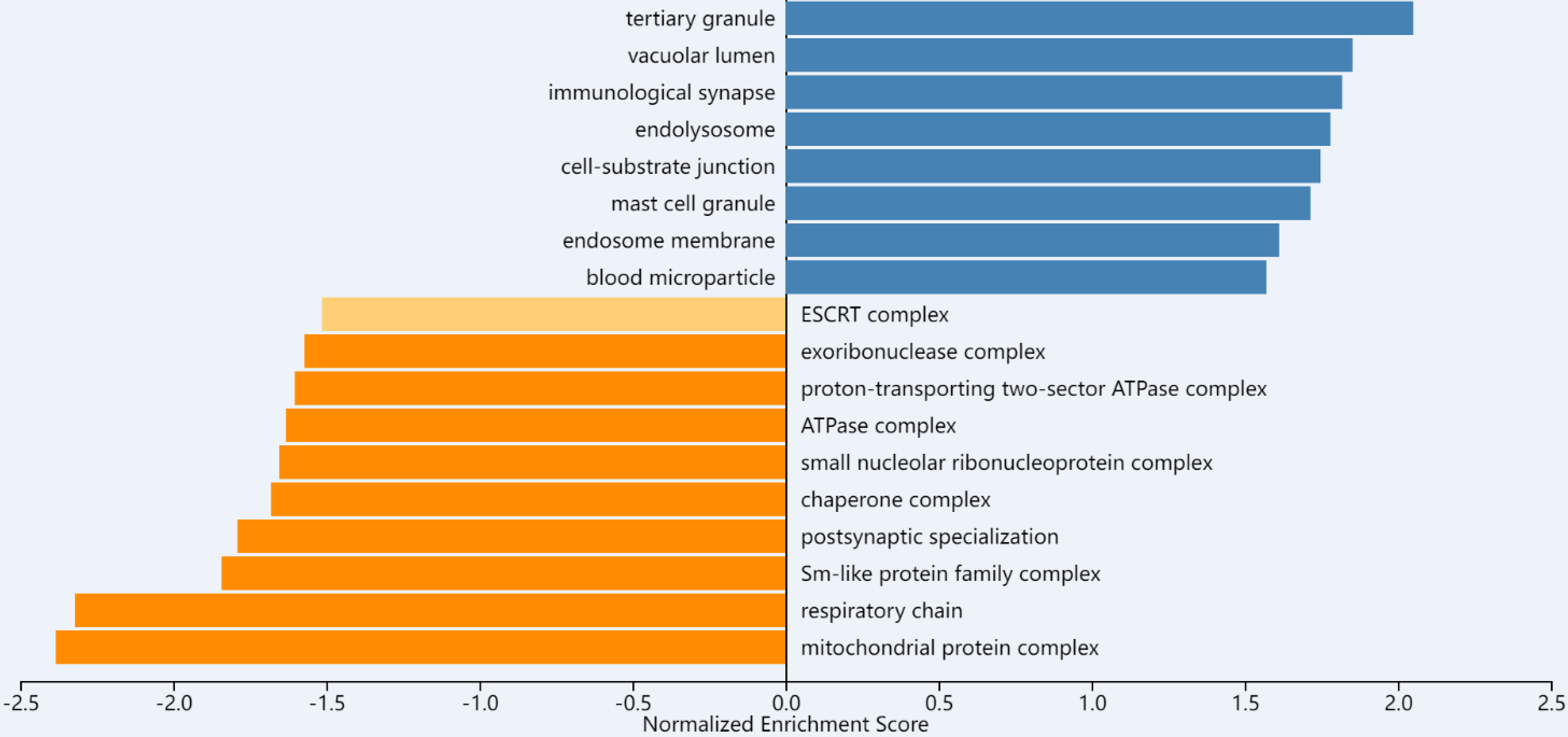

Supplement: Supplementary file 1 [file DataSheet1.ZIP › all raw data/original figures/Figure 7/Figure 7G.png]

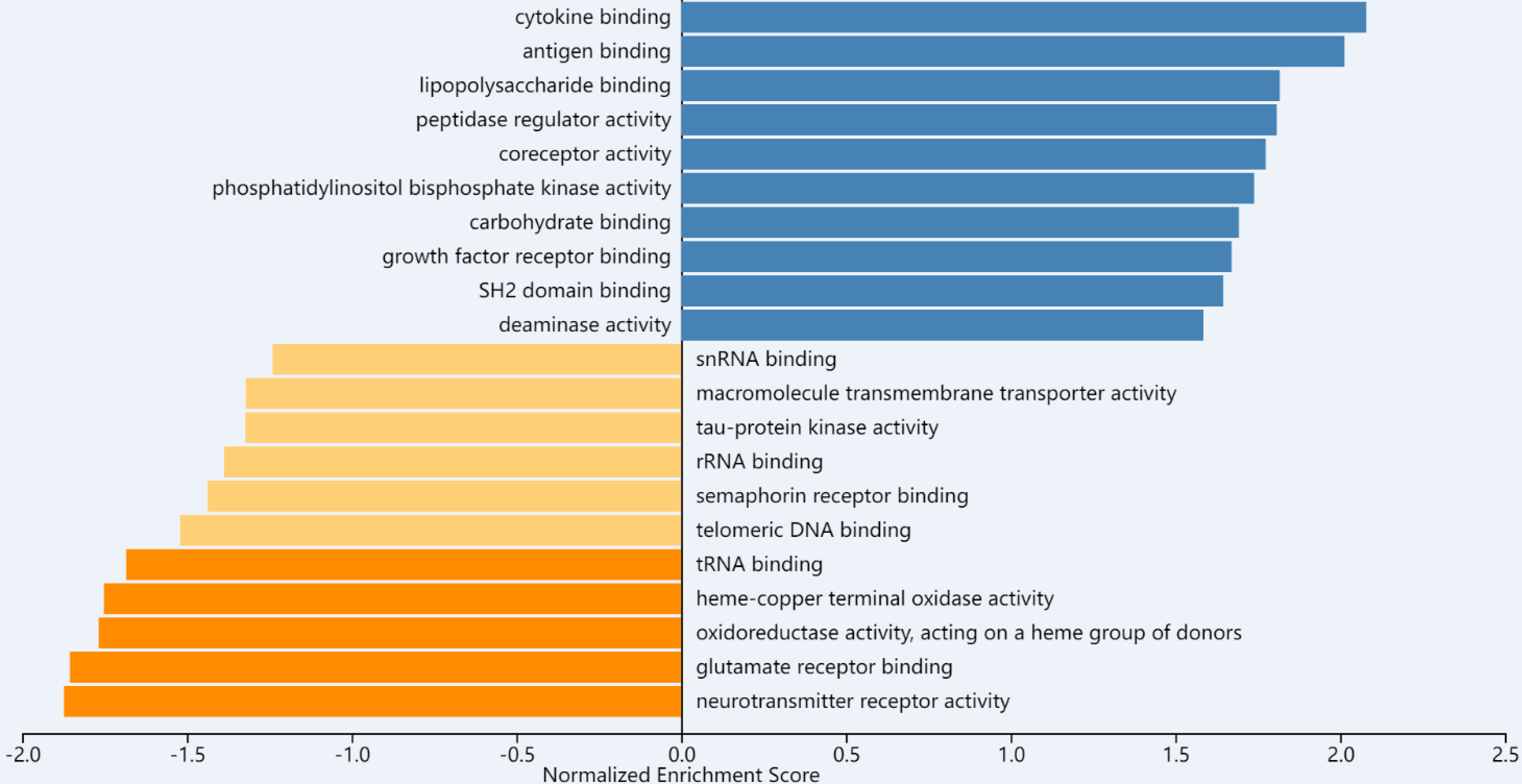

Supplement: Supplementary file 1 [file DataSheet1.ZIP › all raw data/original figures/Figure 7/Figure 7H.png]

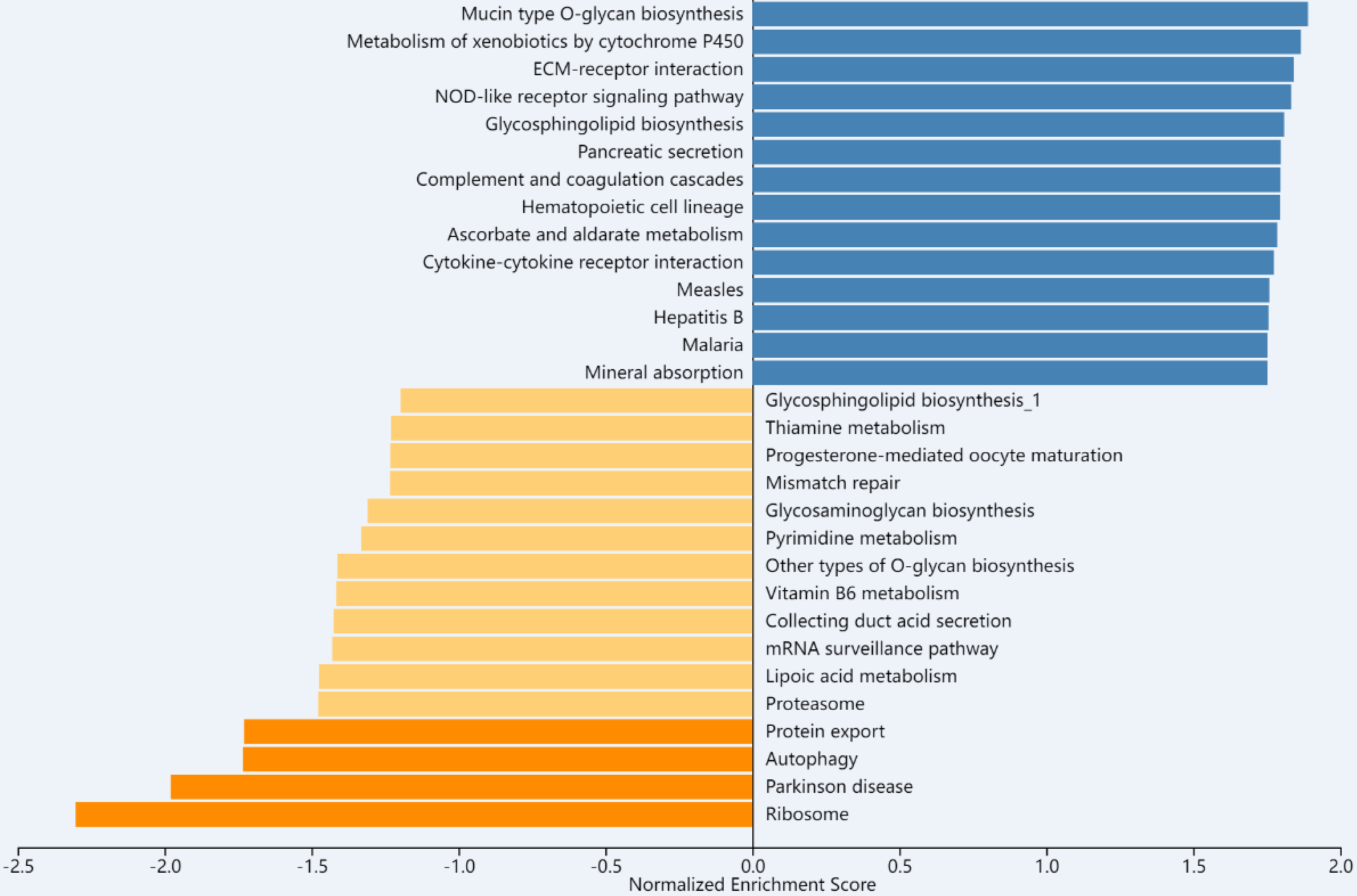

Supplement: Supplementary file 1 [file DataSheet1.ZIP › all raw data/original figures/Figure 7/Figure 7I.png]

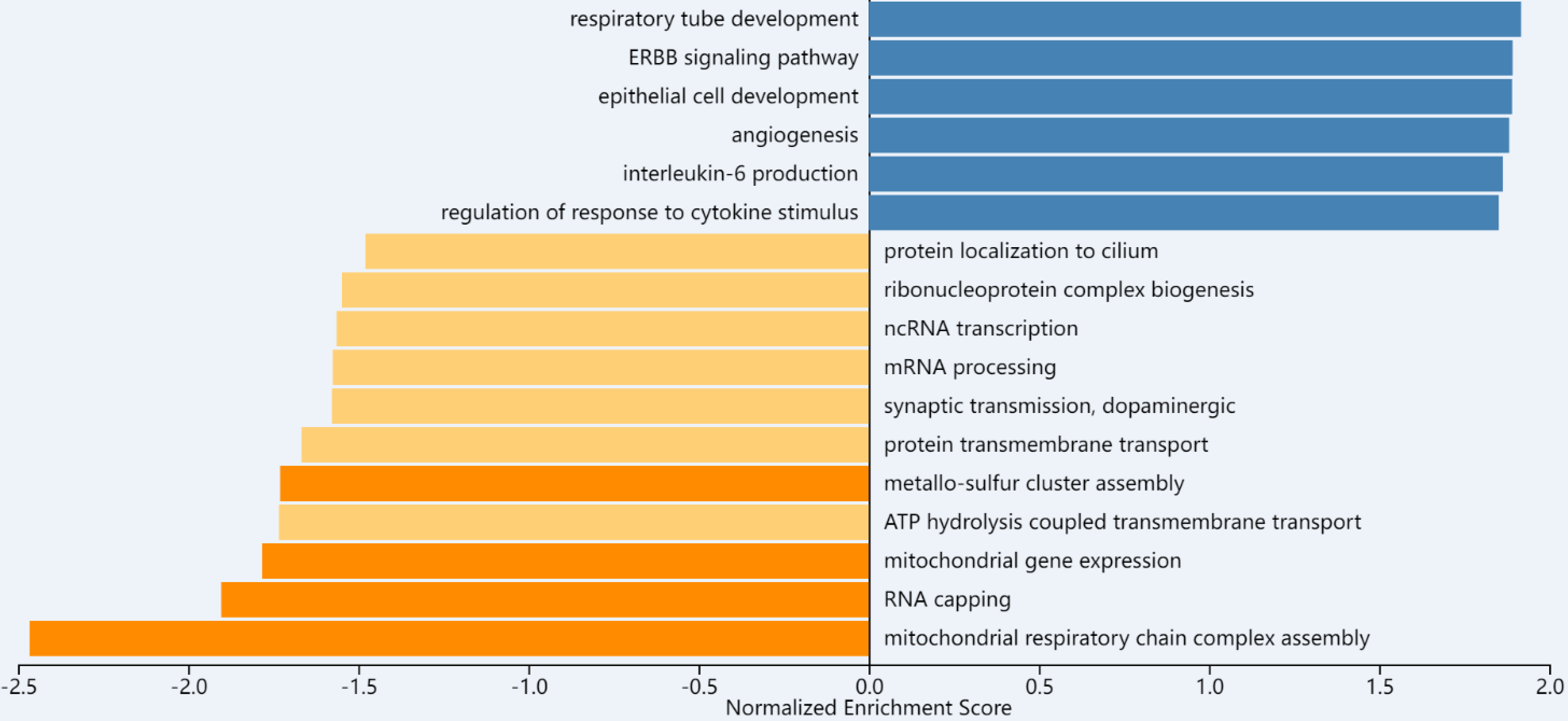

Supplement: Supplementary file 1 [file DataSheet1.ZIP › all raw data/original figures/Figure 7/Figure 7J.png]

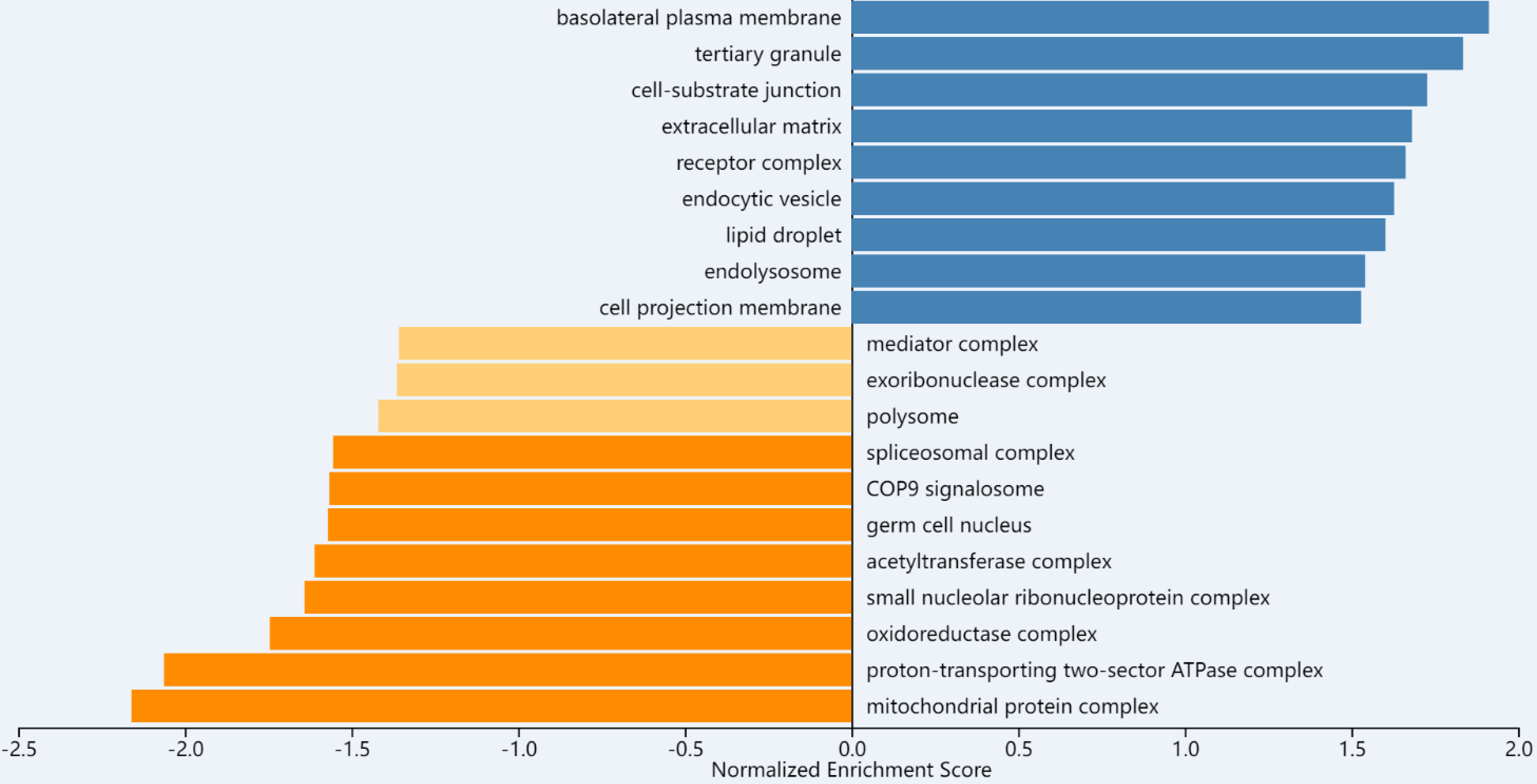

Supplement: Supplementary file 1 [file DataSheet1.ZIP › all raw data/original figures/Figure 7/Figure 7K.png]

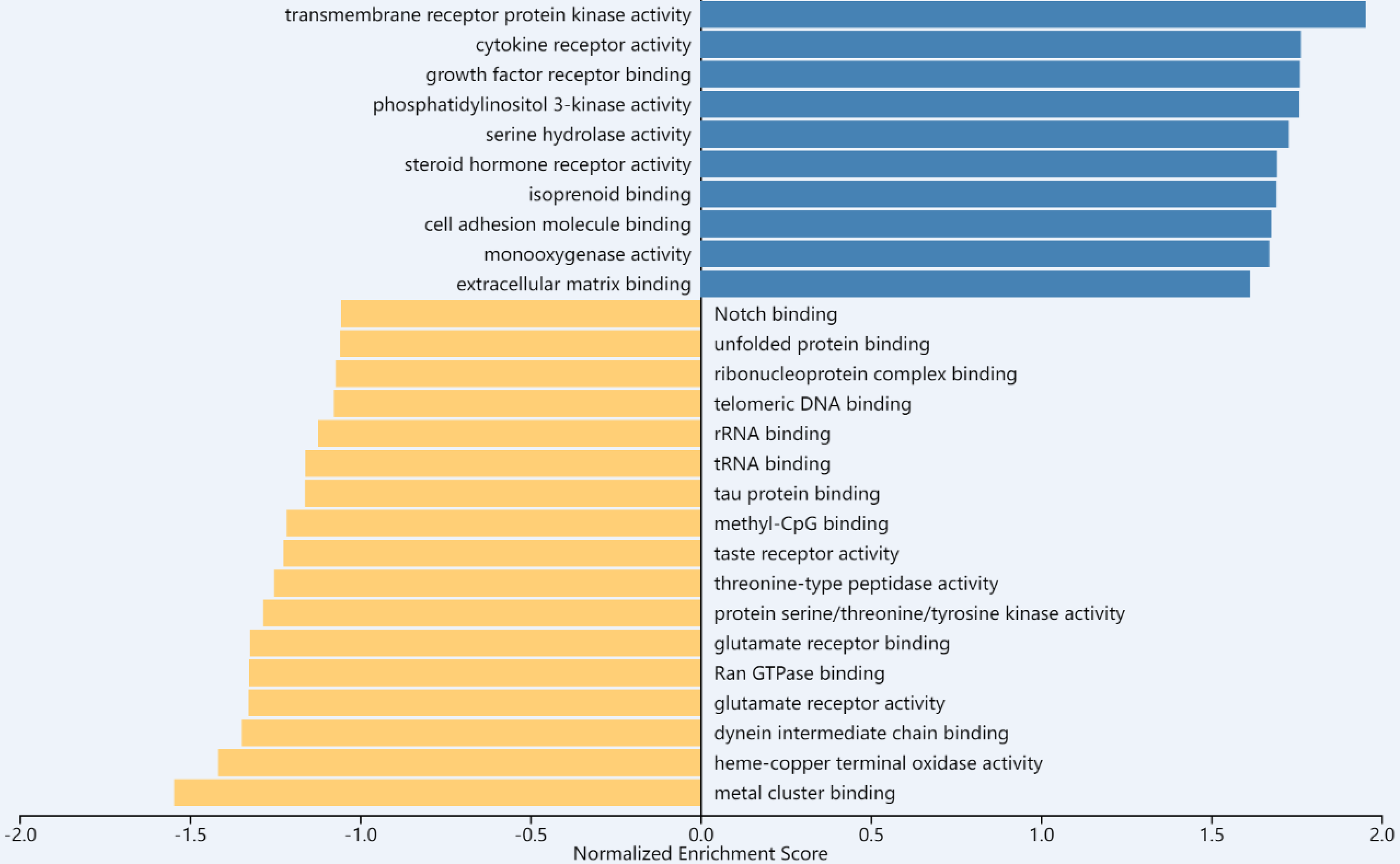

Supplement: Supplementary file 1 [file DataSheet1.ZIP › all raw data/original figures/Figure 7/Figure 7L.png]

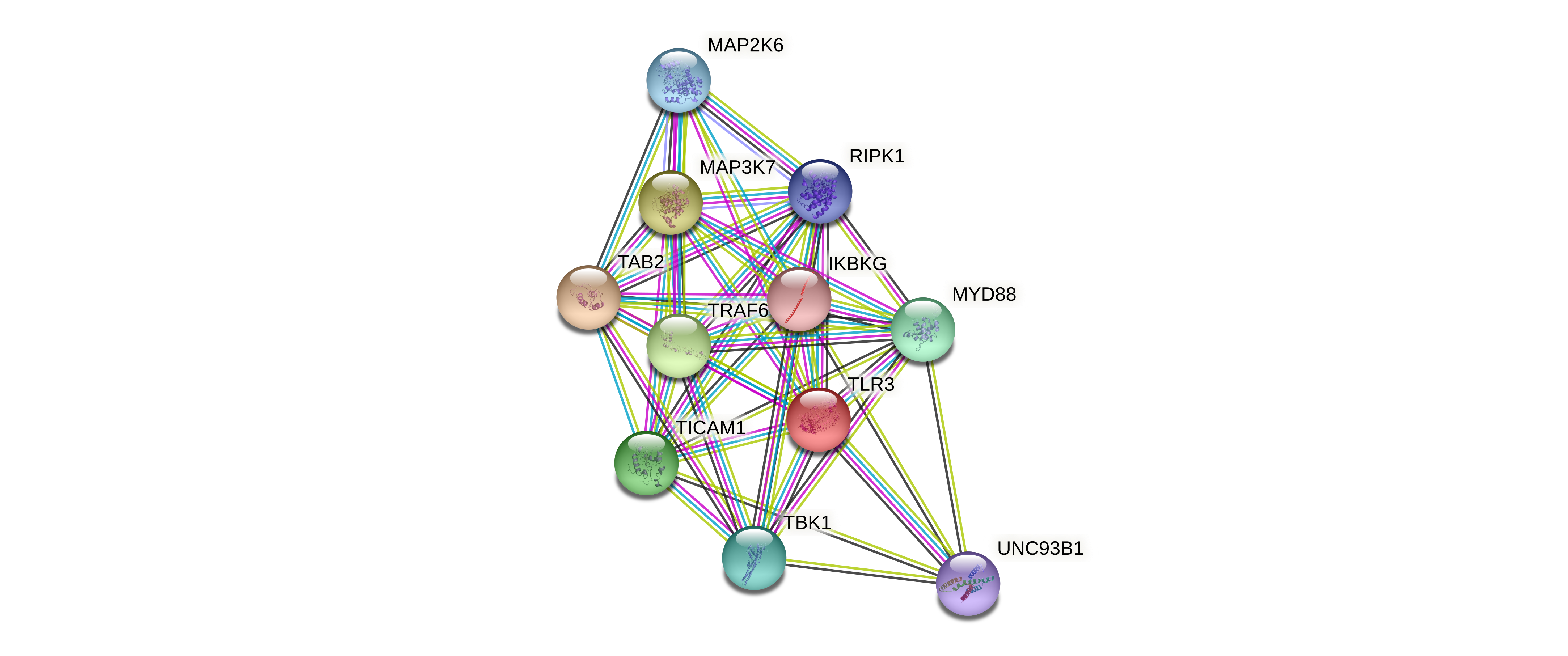

Supplement: Supplementary file 1 [file DataSheet1.ZIP › all raw data/original figures/Figure 8/Figure 8A.png]

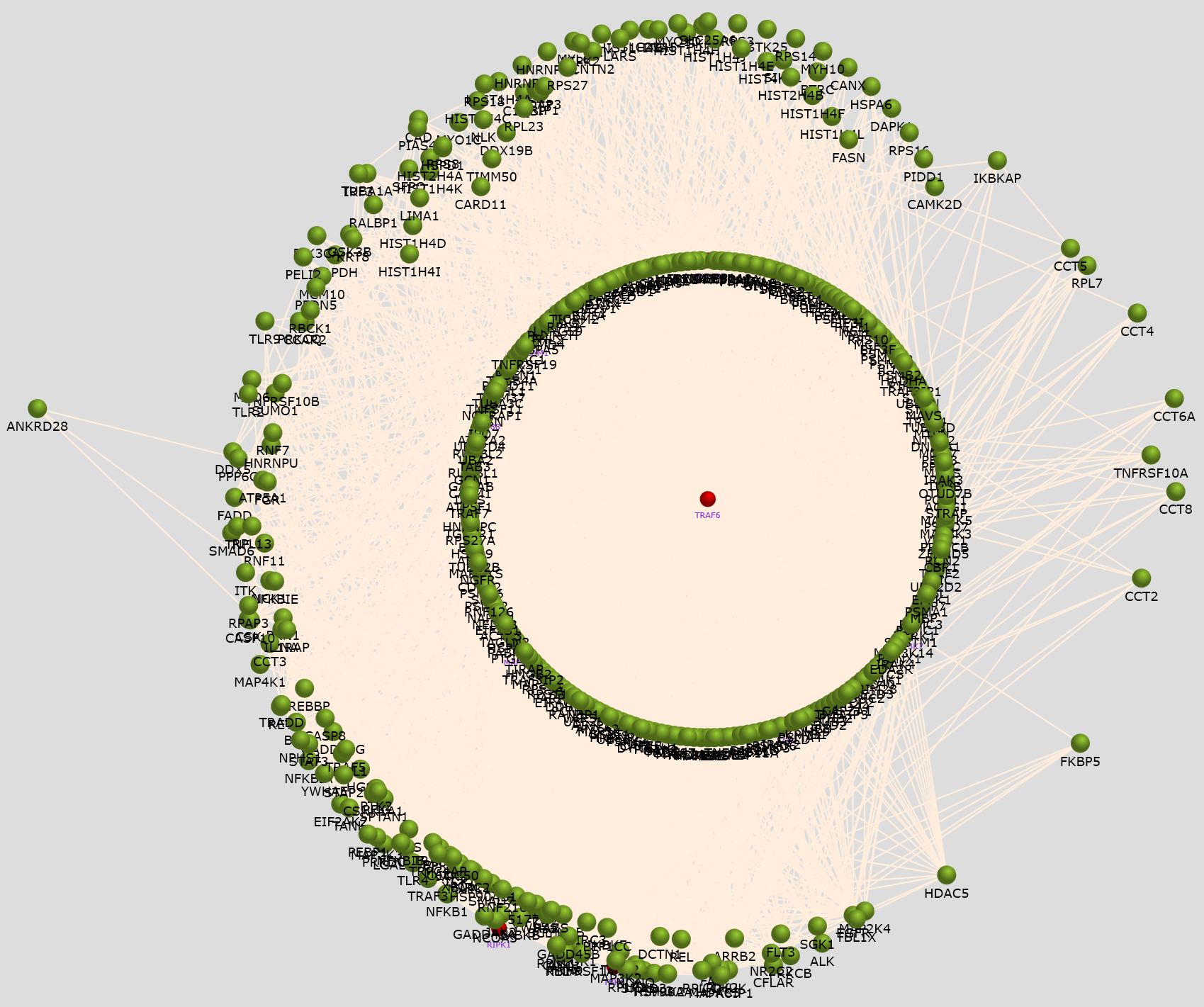

Supplement: Supplementary file 1 [file DataSheet1.ZIP › all raw data/original figures/Figure 8/Figure 8C.png]

# TLR3

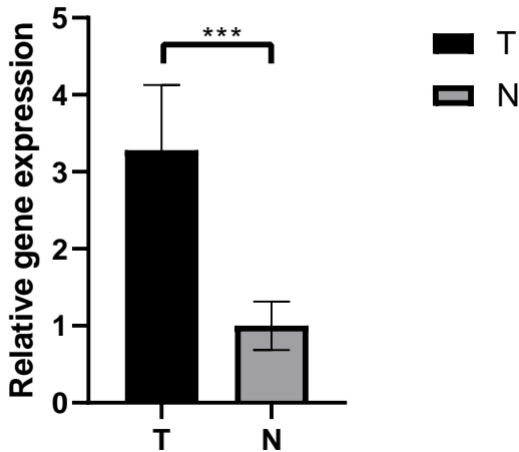

Supplement: Supplementary file 1 [file DataSheet1.ZIP › all raw data/original figures/Figure 9/Figure 9A.pdf]

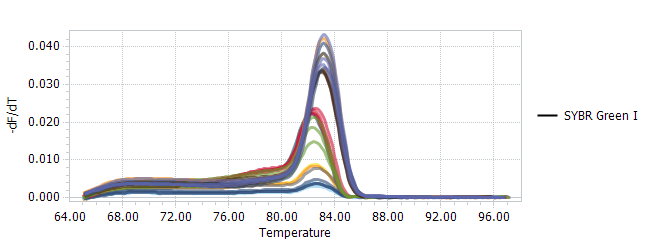

Supplement: Supplementary file 1 [file DataSheet1.ZIP › all raw data/original figures/Figure 9/Figure 9B.png]

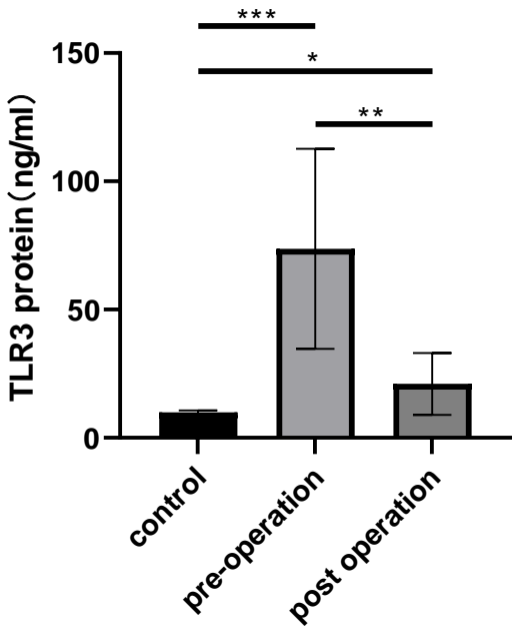

Supplement: Supplementary file 1 [file DataSheet1.ZIP › all raw data/original figures/Figure 9/Figure 9C.pdf]

# Overall Survival

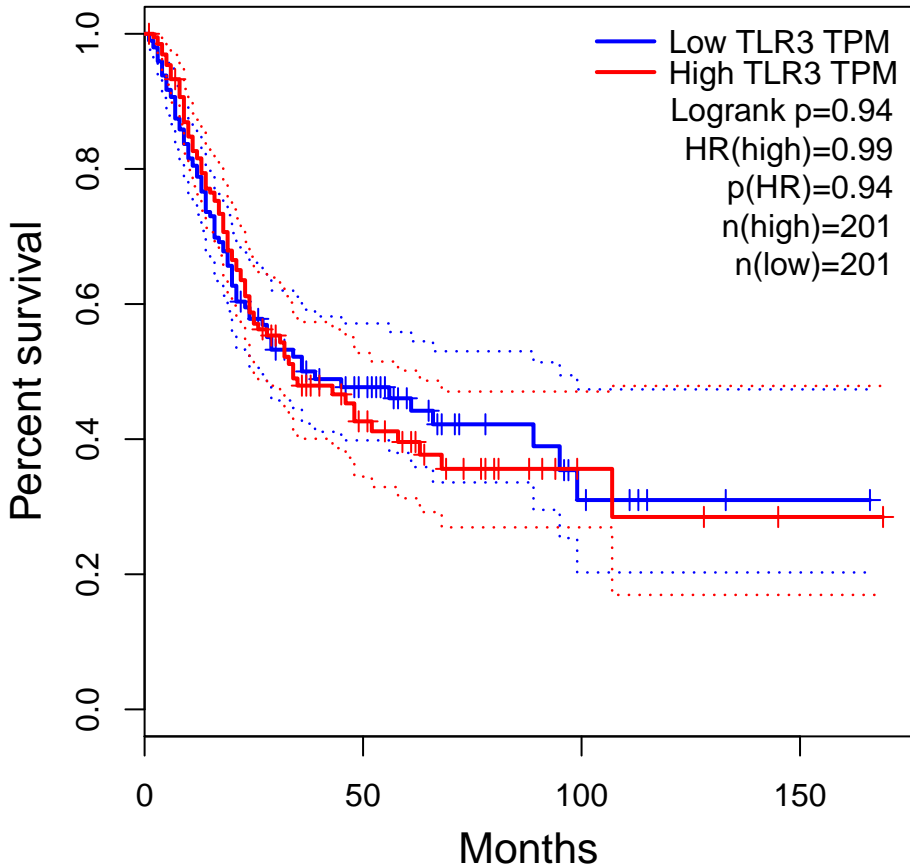

Supplement: Supplementary file 1 [file DataSheet1.ZIP › all raw data/original figures/Supplementary figure 1/A_BLCA_TLR3_survival.pdf]

# Overall Survival

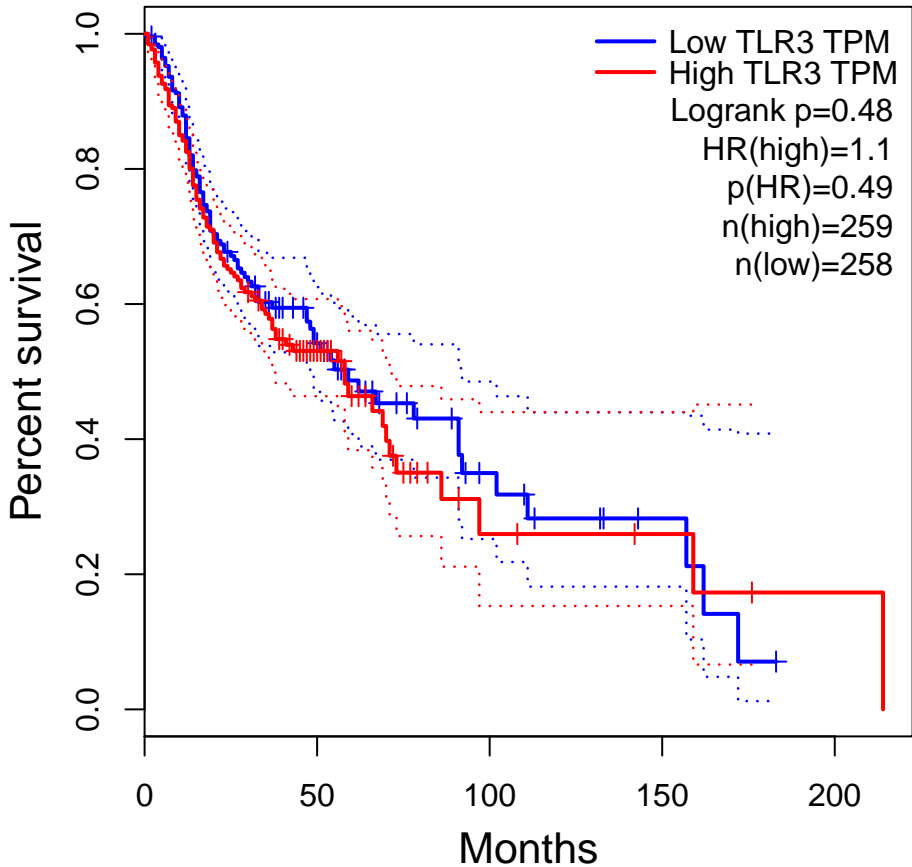

Supplement: Supplementary file 1 [file DataSheet1.ZIP › all raw data/original figures/Supplementary figure 1/B_HNSC_TLR3_survival.pdf]

# Overall Survival

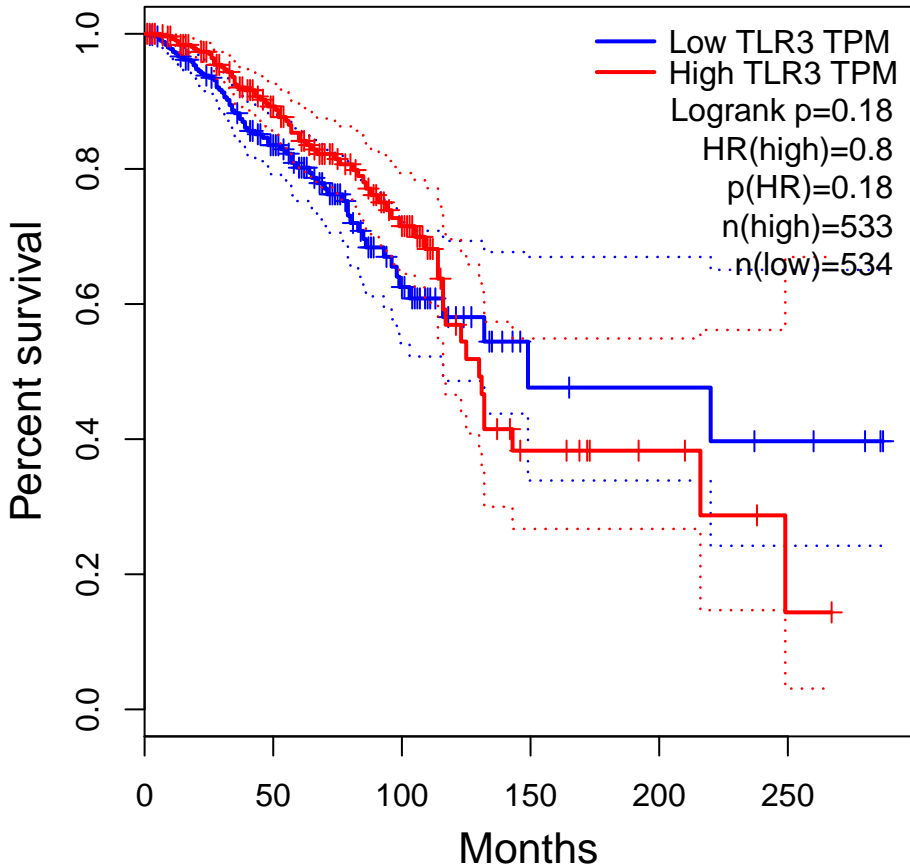

Supplement: Supplementary file 1 [file DataSheet1.ZIP › all raw data/original figures/Supplementary figure 1/C_BRCA_TLR3_survival.pdf]

# Overall Survival

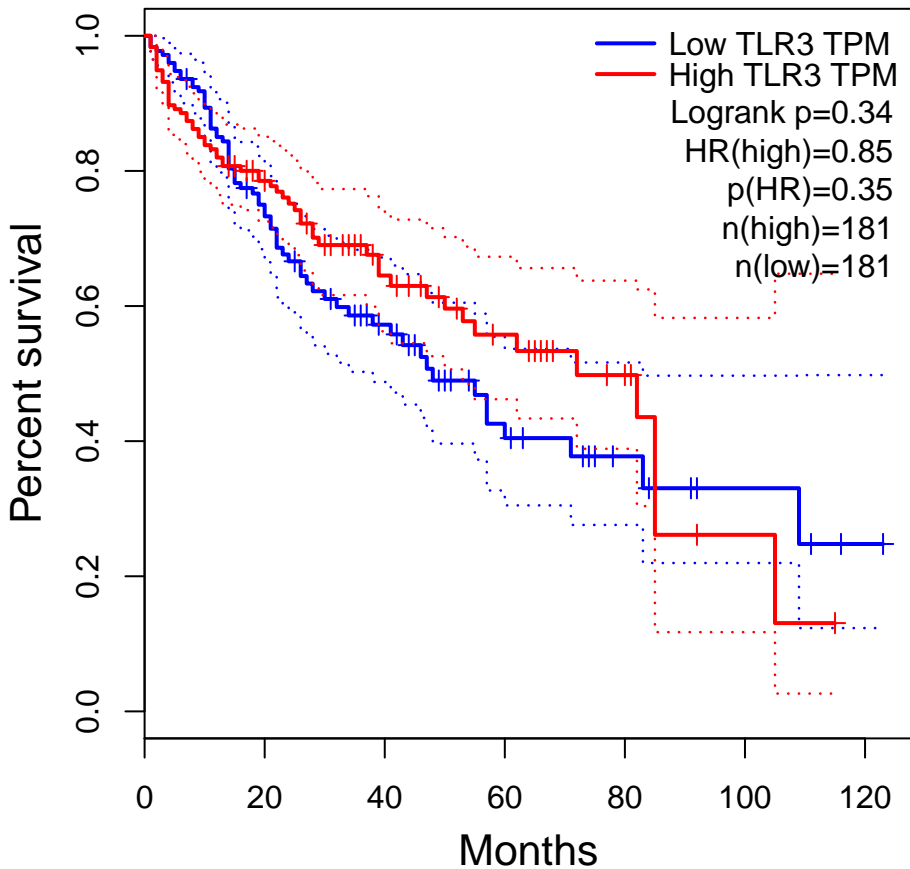

Supplement: Supplementary file 1 [file DataSheet1.ZIP › all raw data/original figures/Supplementary figure 1/D_LIHC_TLR3_survival.pdf]

# Overall Survival

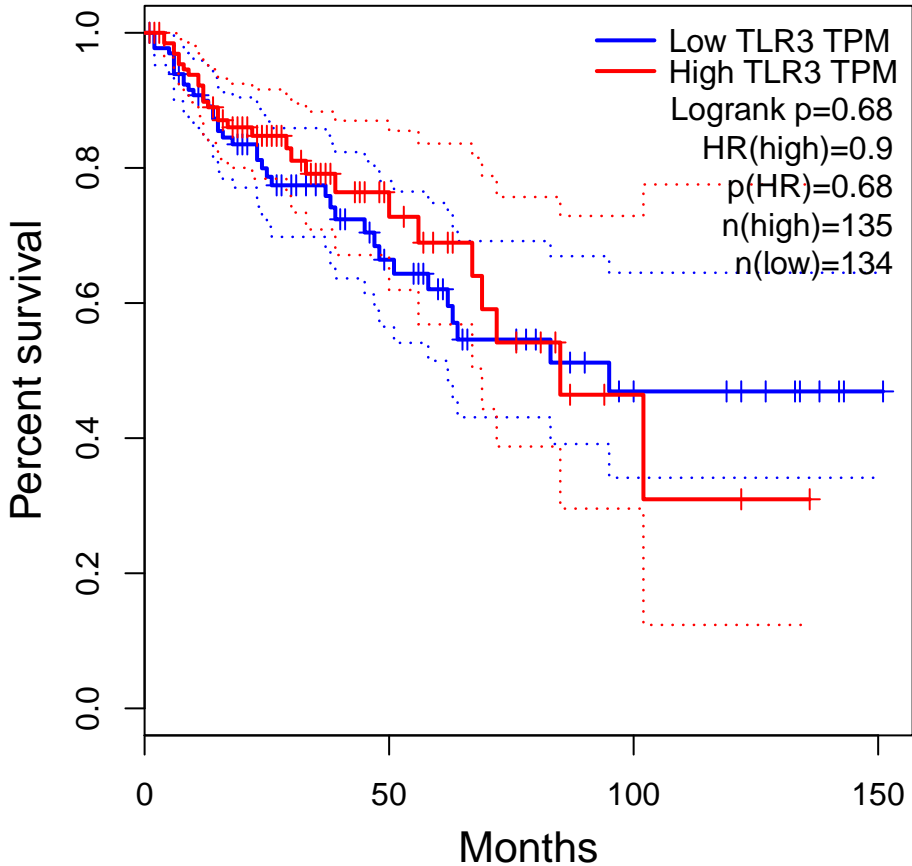

Supplement: Supplementary file 1 [file DataSheet1.ZIP › all raw data/original figures/Supplementary figure 1/E_COAD_TLR3_survival.pdf]

# Overall Survival

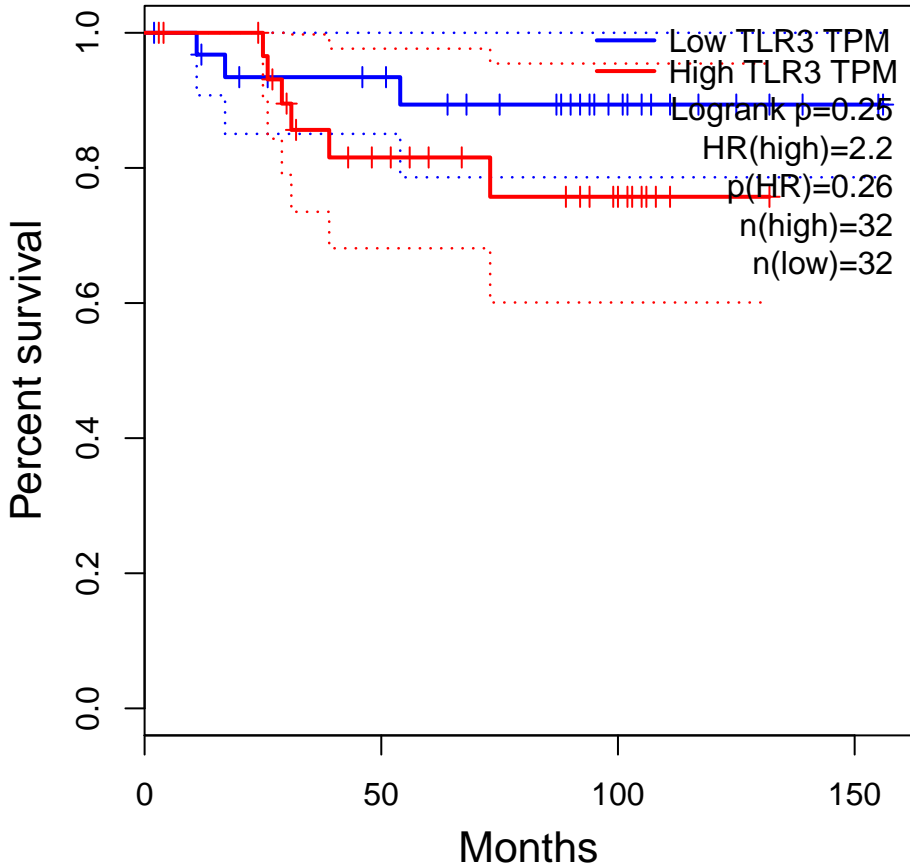

Supplement: Supplementary file 1 [file DataSheet1.ZIP › all raw data/original figures/Supplementary figure 1/F_KICH_TLR3_survival.pdf]

# Overall Survival

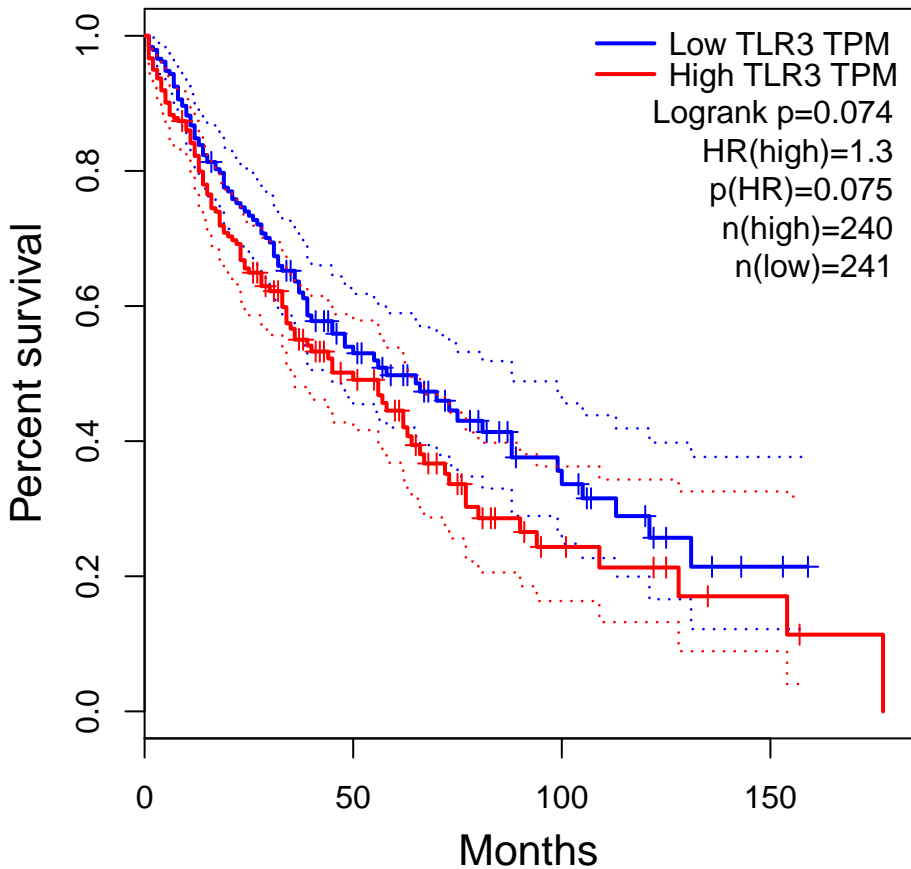

Supplement: Supplementary file 1 [file DataSheet1.ZIP › all raw data/original figures/Supplementary figure 1/G_LUSC_TLR3_survival.pdf]

# Overall Survival

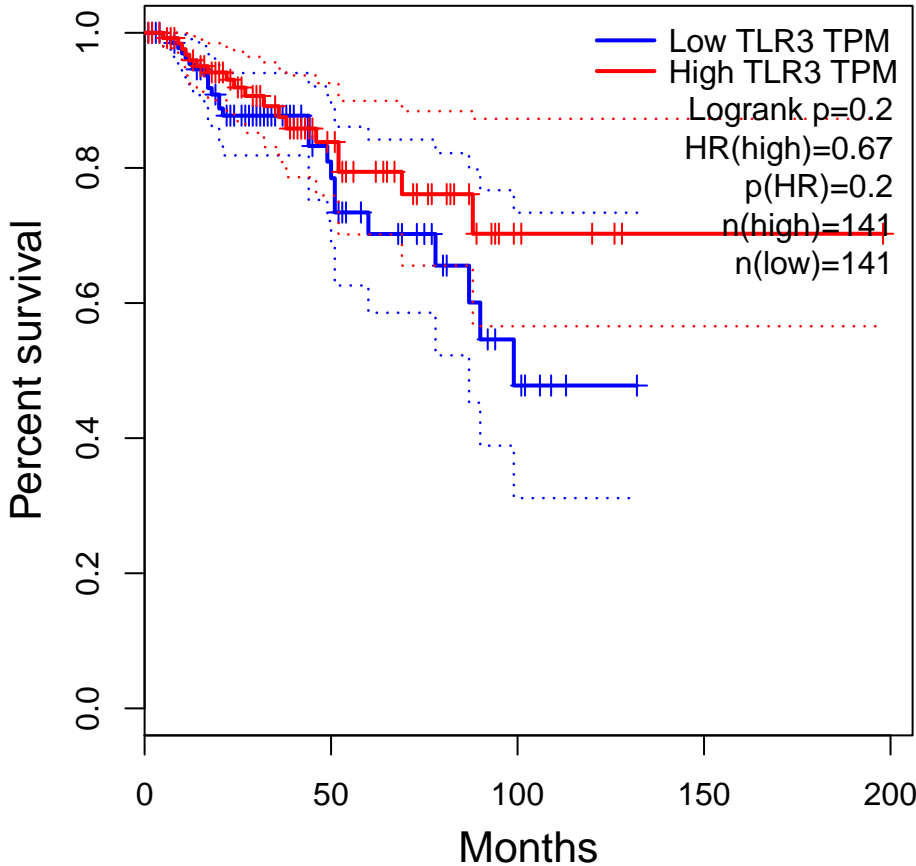

Supplement: Supplementary file 1 [file DataSheet1.ZIP › all raw data/original figures/Supplementary figure 1/H_KIRP_TLR3_survival.pdf]

# Overall Survival

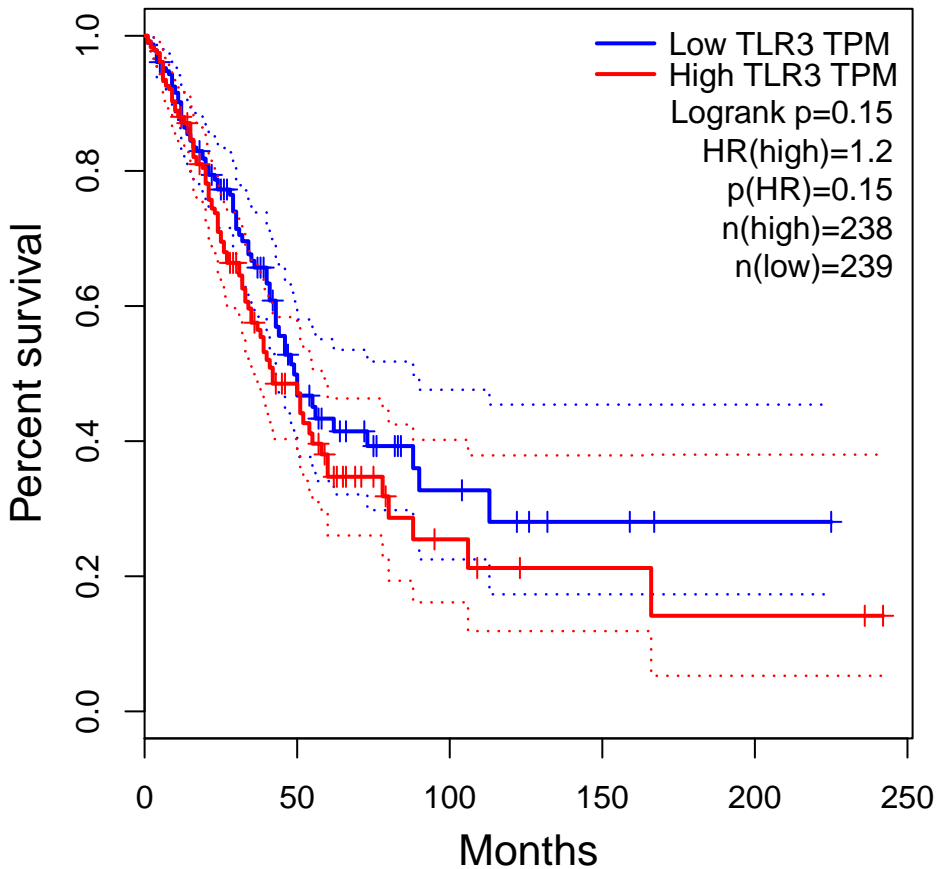

Supplement: Supplementary file 1 [file DataSheet1.ZIP › all raw data/original figures/Supplementary figure 1/I_LUAD_TLR3_survival.pdf]

# Overall Survival

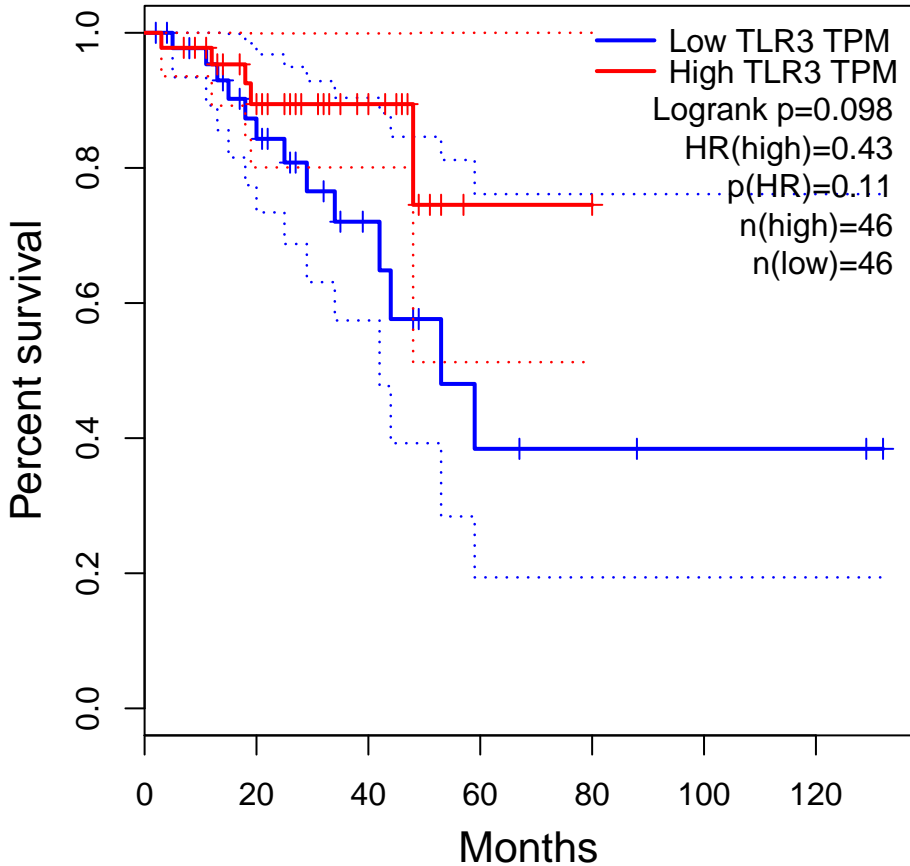

Supplement: Supplementary file 1 [file DataSheet1.ZIP › all raw data/original figures/Supplementary figure 1/J_READ_TLR3_survival.pdf]

# Overall Survival

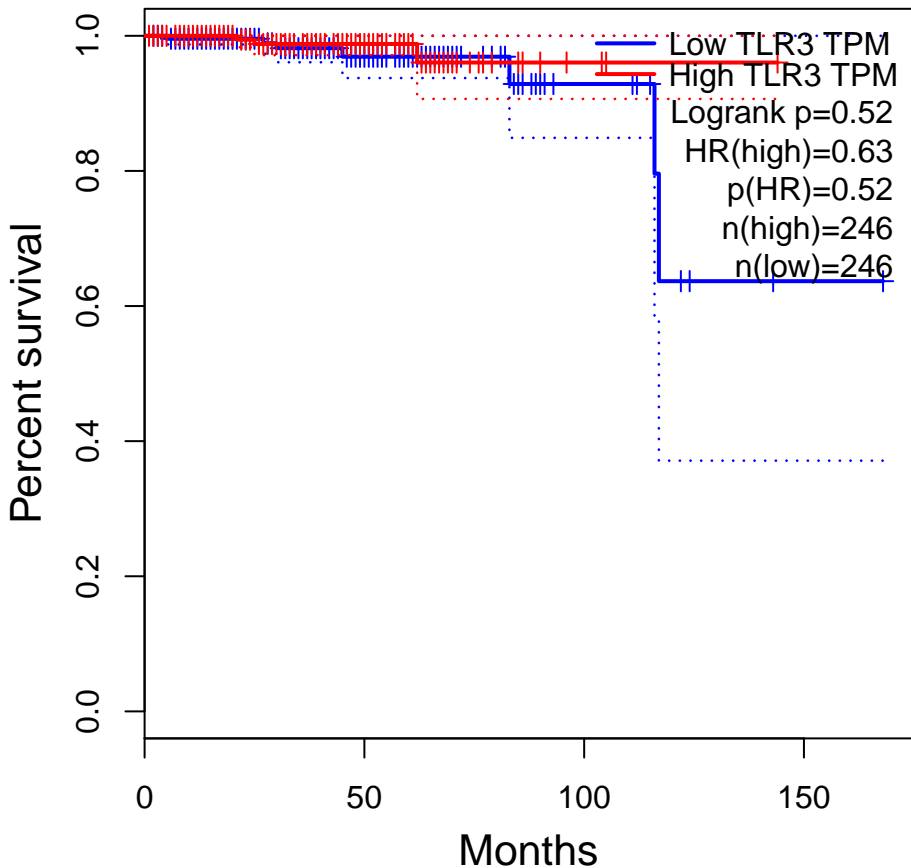

Supplement: Supplementary file 1 [file DataSheet1.ZIP › all raw data/original figures/Supplementary figure 1/K_PRAD_TLR3_survival.pdf]

# Overall Survival

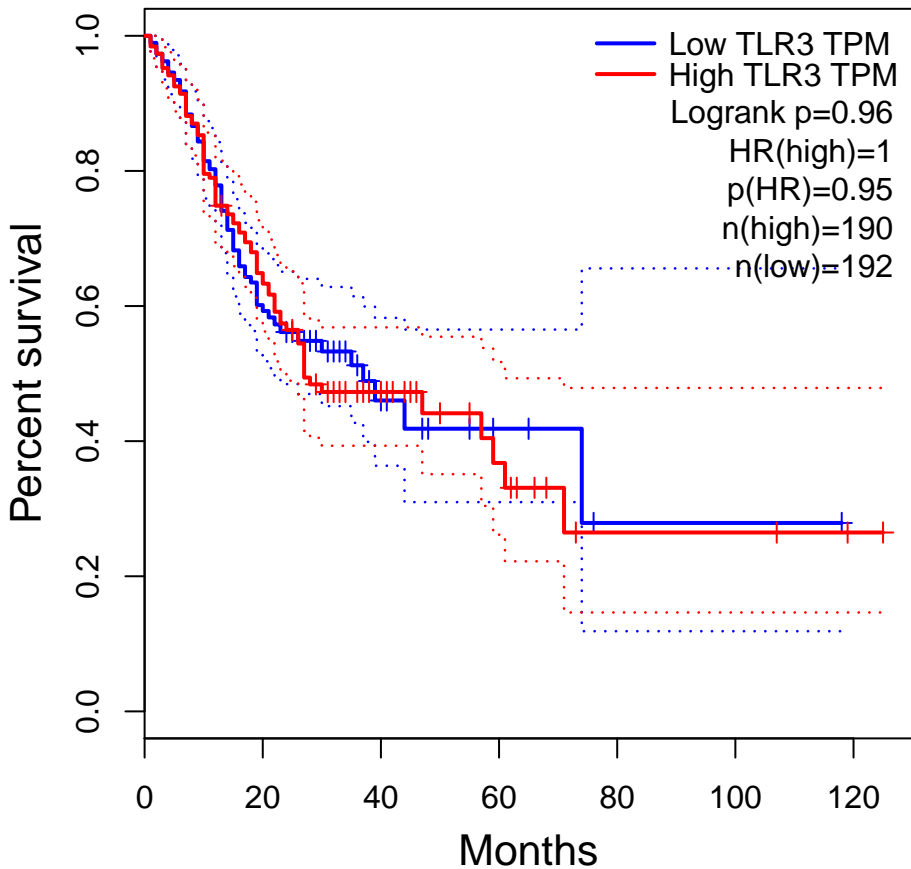

Supplement: Supplementary file 1 [file DataSheet1.ZIP › all raw data/original figures/Supplementary figure 1/L_STAD_TLR3_survival.pdf]

# Overall Survival

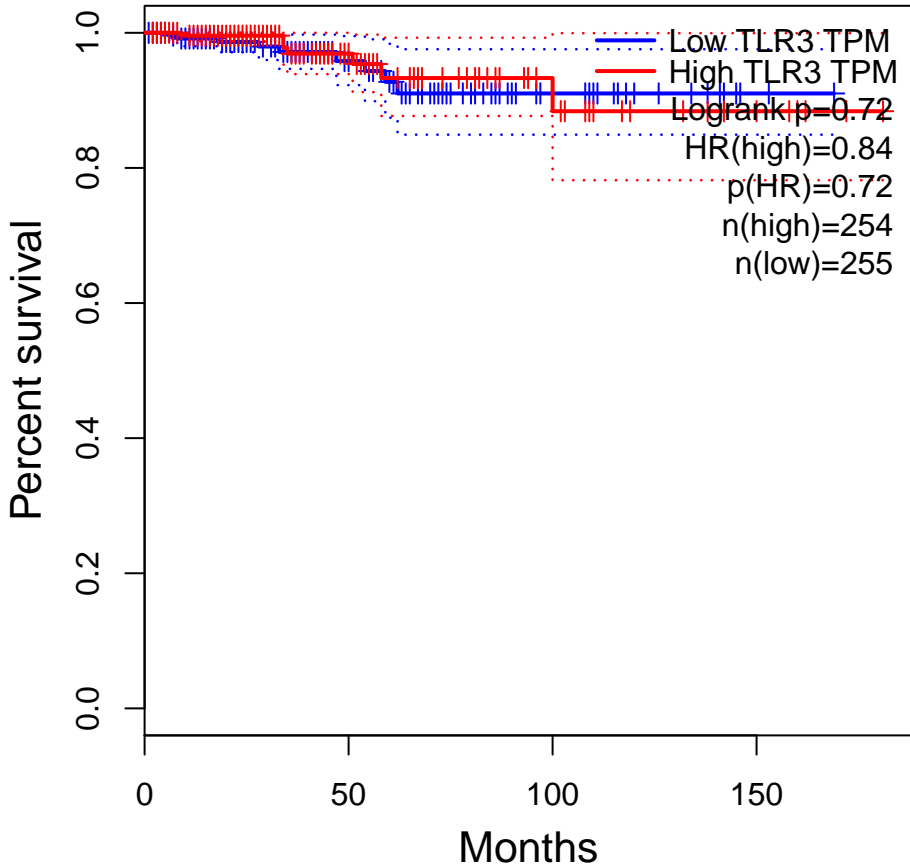

Supplement: Supplementary file 1 [file DataSheet1.ZIP › all raw data/original figures/Supplementary figure 1/M_THCA_TLR3_survival.pdf]

# Overall Survival

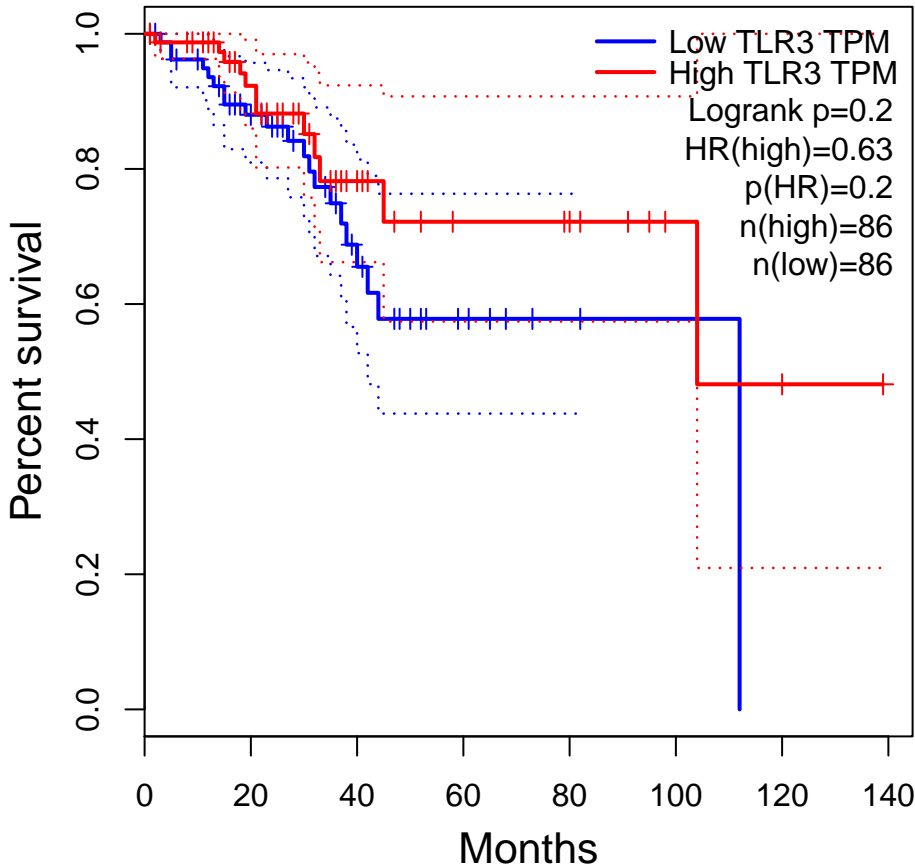

Supplement: Supplementary file 1 [file DataSheet1.ZIP › all raw data/original figures/Supplementary figure 1/N_UCEC_TLR3_survival.pdf]

# Overall Survival

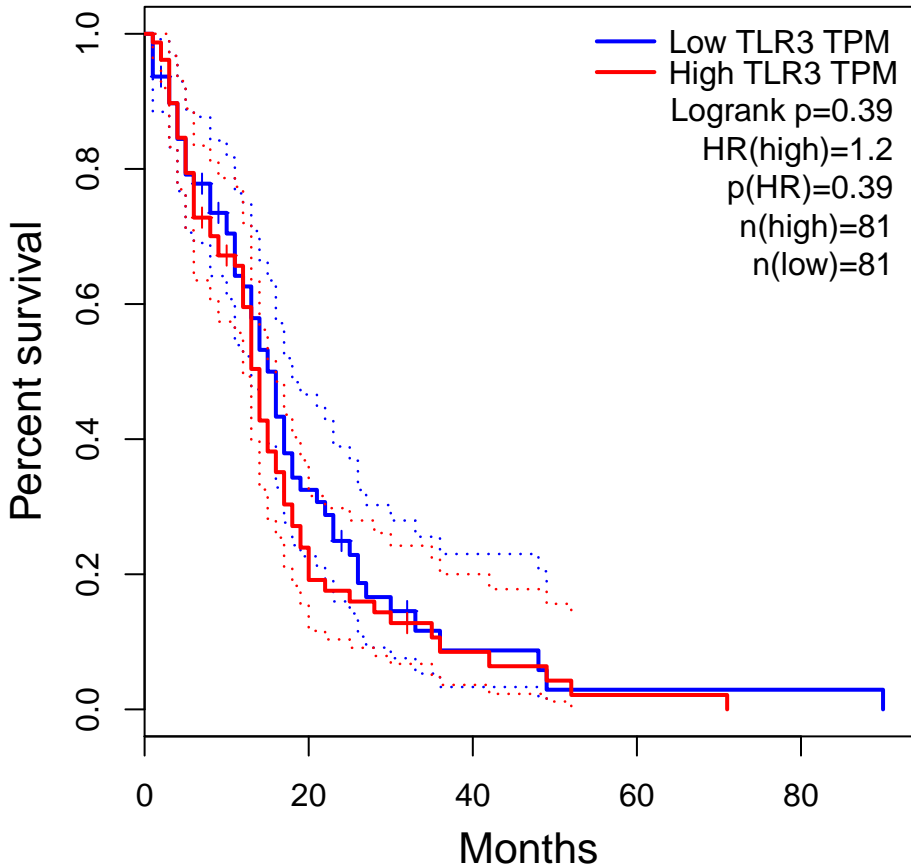

Supplement: Supplementary file 1 [file DataSheet1.ZIP › all raw data/original figures/Supplementary figure 1/O_GBM_TLR3_survival.pdf]

# Overall Survival

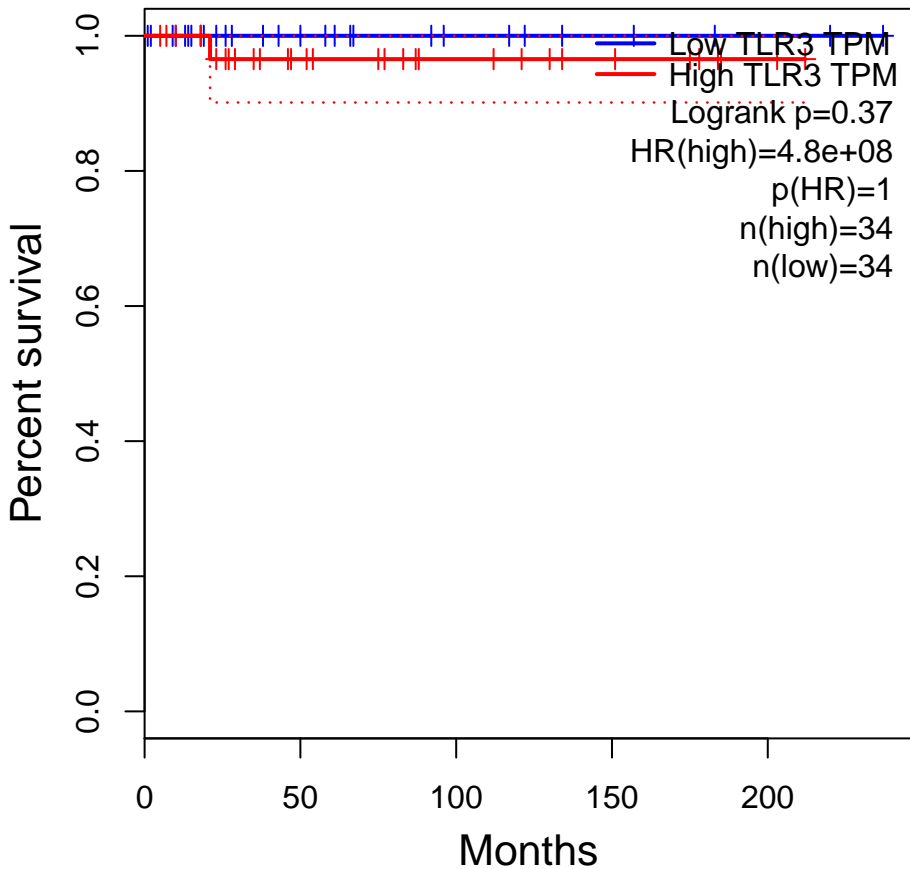

Supplement: Supplementary file 1 [file DataSheet1.ZIP › all raw data/original figures/Supplementary figure 1/P_TGCT_TLR3_survival.pdf]

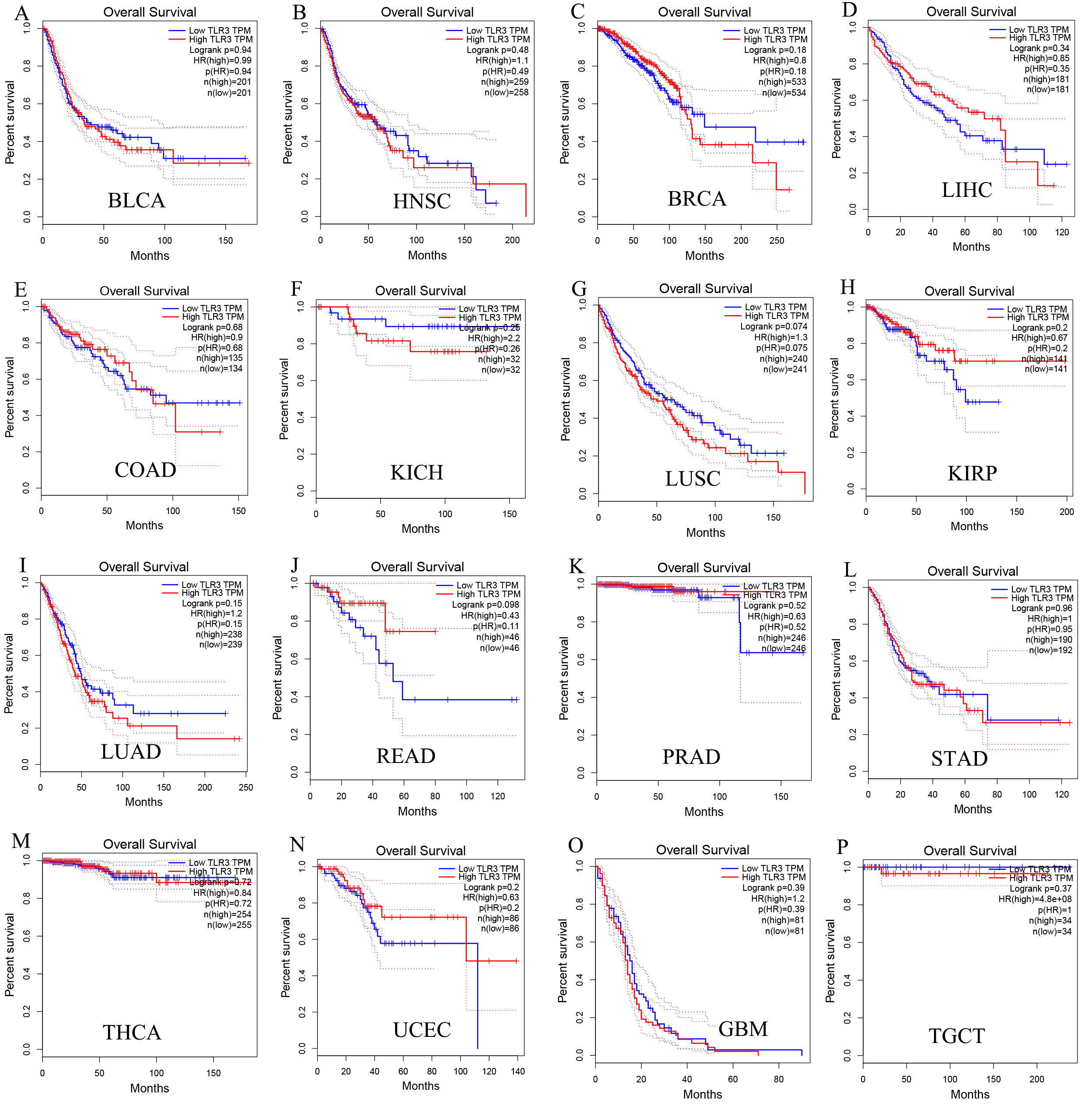

Supplement: Supplementary file 1 [file DataSheet1.ZIP › all raw data/Supplementary figure 1.jpg]
